# LncRNA‐MEG3 Regulates Muscle Mass and Metabolic Homeostasis by Facilitating SUZ12 Liquid–Liquid Phase Separation

**DOI:** 10.1002/advs.202417715

**Published:** 2025-04-26

**Authors:** Yilong Yao, Chao Yan, Haibo Huang, Shilong Wang, Jiaying Li, Yun Chen, Xiaolu Qu, Qi Bao, Lingna Xu, Yuanyuan Zhang, Danyang Fan, Xia He, Yanwen Liu, Yongsheng Zhang, Yalan Yang, Zhonglin Tang

**Affiliations:** ^1^ Shenzhen Branch Guangdong Laboratory of Lingnan Modern Agriculture Key Laboratory of Livestock and Poultry Multi‐omics of MARA Agricultural Genomics Institute at Shenzhen Chinese Academy of Agricultural Sciences Shenzhen 518124 China; ^2^ Kunpeng Institute of Modern Agriculture at Foshan Chinese Academy of Agricultural Sciences Foshan 528226 China; ^3^ Key Laboratory of Livestock and Poultry Multi‐Omics of MARA Agricultural Genomics Institute at Shenzhen Chinese Academy of Agricultural Sciences Shenzhen 518124 China; ^4^ Beijing Tongren Hospital Capital Medical University Beijing 100730 China; ^5^ Key Laboratory of Agricultural Animal Genetics Breeding and Reproduction of Ministry of Education and Key Lab of Swine Genetics and Breeding of Ministry of Agriculture and Rural Affairs Huazhong Agricultural University Wuhan 430070 China; ^6^ School of Animal Science and Technology Foshan University Foshan 528225 China

**Keywords:** fat infiltration, lncRNA‐MEG3, muscle atrophy, oxidative muscle fibers, SUZ12 LLPS

## Abstract

Skeletal muscle plays a crucial role in maintaining motor function and metabolic homeostasis, with its loss or atrophy leading to significant health consequences. Long non‐coding RNAs (lncRNAs) have emerged as key regulators in muscle biology; however, their precise roles in muscle function and pathology remain to be fully elucidated. This study demonstrates that lncRNA maternally expressed gene 3 (MEG3) is preferentially expressed in slow‐twitch muscle fibers and dynamically regulated during muscle development, aging, and in the context of Duchenne muscular dystrophy (DMD). Using both loss‐ and gain‐of‐function mice models, this study shows that lncRNA‐MEG3 is critical for preserving muscle mass and function. Its depletion leads to muscle atrophy, mitochondrial dysfunction, and impaired regenerative capacity, while overexpression enhances muscle mass, increases oxidative muscle fiber content, and improves endurance. Notably, lncRNA‐MEG3 overexpression in MDX mice significantly alleviates muscle wasting and adipose tissue infiltration. Mechanistically, this study uncovers a novel interaction between lncRNA‐MEG3 and the polycomb repressive complex 2 (PRC2), where lncRNA‐MEG3 binds to SUZ12 polycomb repressive complex 2 subunit (Suz12), stabilizes PRC2, facilitates SUZ12 liquid–liquid phase separation (LLPS), and regulates the epigenetic modulation of four and a half lim domains 3 (Fhl3) and ring finger protein 128 (Rnf128). These findings not only highlight the crucial role of lncRNA‐MEG3 in muscle homeostasis but also provide new insights into lncRNA‐based therapeutic strategies for muscle‐related diseases.

## Graphical abstract

This study has identified lncRNA‐MEG3 as a key regulator of muscle mass, promoting slow‐twitch muscle fibers and preventing muscle atrophy. By stabilizing the SUZ12/PRC2 complex through liquid–liquid phase separation (LLPS), lncRNA‐MEG3 influences mitochondrial function and lipid metabolism. These findings highlight its potential as a therapeutic target for muscle‐wasting diseases like Duchenne muscular dystrophy (DMD).

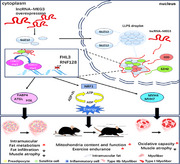

## 1 Introduction

Skeletal muscle is a crucial organ for maintaining vital physiological functions, including movement, posture maintenance, and energy metabolism.^[^ ^1^ ^]^ As a primary metabolic organ, skeletal muscle supports systemic metabolic balance by regulating energy homeostasis. Muscle loss and atrophy precipitate functional impairment, degenerative remodeling, and the pathogenesis of muscle‐related disorders. Fat infiltration and muscle fiber type transition, such as the shift from type I to type IIb fibers, are hallmark features of muscle degeneration.^[^ ^2^ ^]^ These changes not only impair muscle function but also exacerbate metabolic disorders, contributing to the development and progression of related diseases.^[^ ^3^ ^]^ Additionally, mitochondrial dysfunction, closely linked to energy metabolism and oxidative stress, serves as a key driver of muscle atrophy.^[^ ^4^ ^]^ Therefore, a deeper understanding of the factors regulating skeletal muscle homeostasis and muscle atrophy, along with their molecular mechanisms, is of critical importance for preserving muscle mass and for the early diagnosis and treatment of muscle diseases.

LncRNA, defined as transcripts longer than 200 nucleotides with no protein‐coding potential, have emerged as critical regulators of various biological processes, including myogenesis and muscle‐related diseases.^[^ ^5^, ^6^ ^]^ For instance, linc‐muscle differentiation 1 and lnc‐mitochondrial genome are highly expressed in skeletal muscle and regulate muscle cell differentiation, with their altered expression being linked to muscle atrophy and hypertrophy.^[^ ^7^, ^8^ ^]^ LncRNA exerts their regulatory functions through various mechanisms. Unlike mRNAs, certain lncRNAs exert their regulatory functions by interacting with multiple proteins to form intricate regulatory networks that modulate gene expression.^[^ ^9^, ^10^ ^]^ For example, X inactive specific transcript binds to spen family transcriptional repressor through its repetitive ring structure to silence gene expression.^[^ ^11^ ^]^ Additionally, some lncRNA serve as scaffolds for membrane less subcellular structures or nuclear bodies (NBs).^[^ ^9^ ^]^ These specialized structures often contain proteins with low‐complexity domains or intrinsically disordered region (IDR), which drive droplet formation via LLPS. Notably, lncRNA can accelerate LLPS in vitro by capturing IDR‐containing proteins or altering their conformations.^[^ ^12^ ^]^ However, there have been no reports on lncRNA regulating skeletal muscle size, growth, metabolism, and diseases through LLPS.

LncRNA‐MEG3, encoded by the imprinted Dlk1‐Dio3 locus, is widely recognized for its tumor‐suppressive functions and is frequently downregulated in diverse malignancies, including breast, liver, colorectal, cervical, gastric, and ovarian cancers.^[^ ^13^ ^]^ Beyond its roles in oncogenesis, emerging studies have highlighted its biological significance in various tissues, including skeletal muscle, as well as its involvement in developmental processes.^[^ ^14^, ^15^, ^16^ ^]^ Nearly a decade ago, initial investigations identified lncRNA‐MEG3 as highly expressed in adult skeletal muscle, with functions predominantly associated with the regulation of muscle cell proliferation, differentiation, and regeneration.^[^ ^17^ ^]^ Our previous work demonstrated that lncRNA‐MEG3 is conserved across species, including mice, pigs, and humans, serves as a marker for porcine meat production traits, and regulates myogenesis through the miR‐133a‐3p/proline rich transmembrane protein 2 axis.^[^ ^18^, ^19^ ^]^ Furthermore, recent findings revealed that lncRNA‐MEG3 knockdown in obese mice significantly reduced Akt phosphorylation levels in skeletal muscle, leading to impaired insulin sensitivity.^[^ ^20^, ^21^ ^]^ These findings suggest that lncRNA‐MEG3 plays pivotal roles in muscle physiology, extending beyond myogenesis to encompass metabolic programming and potential involvement in muscle‐related diseases. However, its broader regulatory functions and underlying mechanisms remain largely unexplored, warranting further investigation.

In this study, we demonstrated that lncRNA‐MEG3 is crucial for regulating muscle size and mass. Using loss‐of‐function mice models in skeletal muscle, we showed that lncRNA‐MEG3 depletion led to muscle atrophy, increased fat deposition in muscle tissue, impaired skeletal muscle regeneration, and reduced mitochondrial ATP production. Transgenic overexpression of lncRNA‐MEG3 in skeletal muscle promoted the conversion of muscle fibers to an oxidative phenotype, enhanced muscle mass and strength, and served as a novel regulator of skeletal muscle fat infiltration. By integrating RNA sequencing (RNA‐seq), chromatin immunoprecipitation sequencing (CUT&tag), and in vitro/in vivo LLPS experiments, we revealed that lncRNA‐MEG3 directly interacts with SUZ12 to regulate its LLPS, maintaining histone h3 lysine 27 tri‐methylation (H3K27me3) modifications of key muscle‐specific genes, such as Fhl3 and Rnf128. These findings not only highlight lncRNA‐MEG3 as a key regulator of muscle size, quality, and metabolic remodeling but also suggest its potential as a therapeutic target for muscle atrophy and fat infiltration‐related diseases.

## 2 Results

### 2.1 Temporal and Tissue‐Specific Expression of LncRNA‐MEG3 in Skeletal Muscle Development and Disease

**Figure 1:**
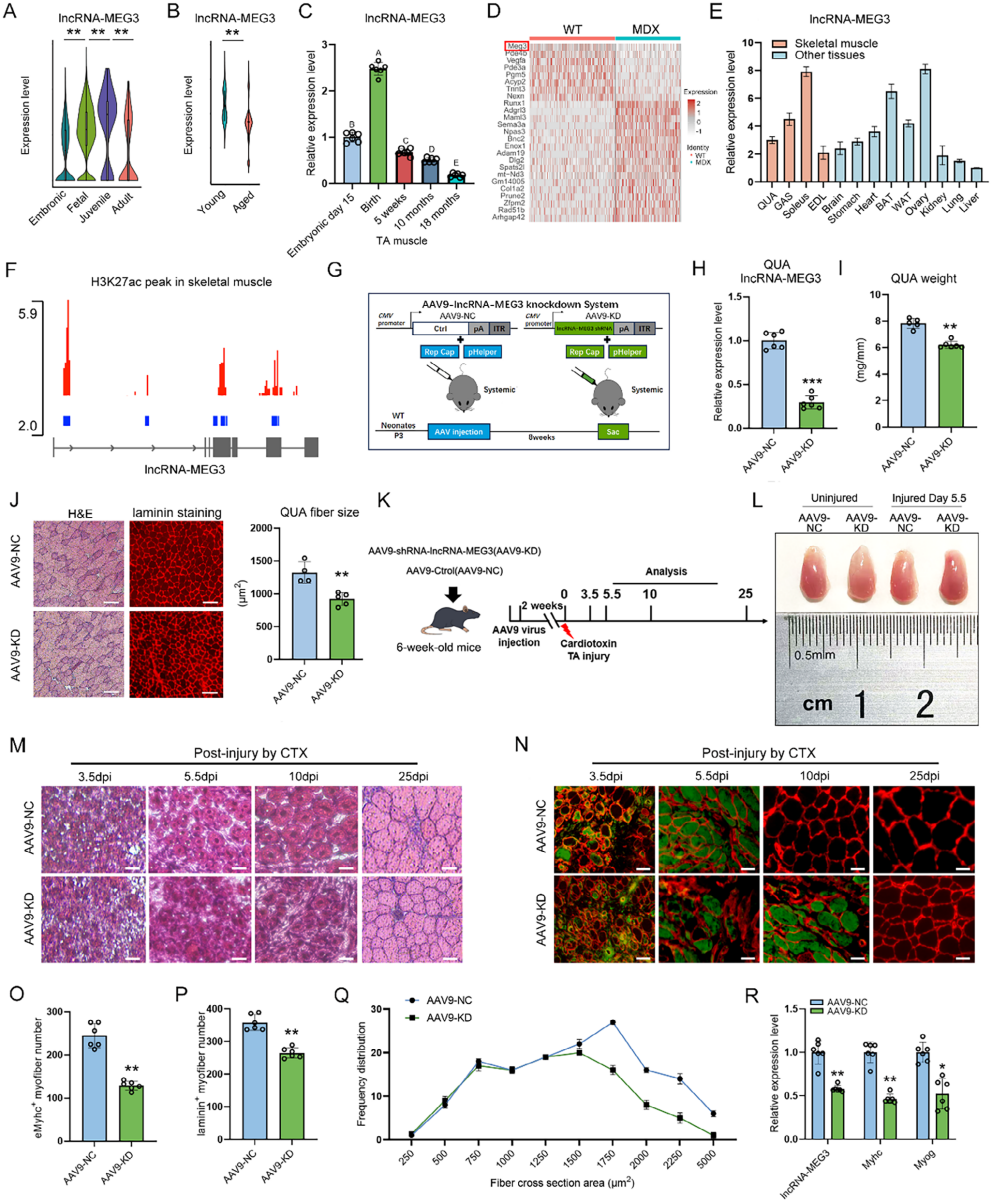
LncRNA‐MEG3 controls the development of skeletal muscle mass and regeneration. A) Violin plots depicting the expression levels of lncRNA‐MEG3 across the MuSCs cluster in human skeletal muscle at different developmental stages (embryonic, fetal, juvenile, and adult) (*n* = 3). B) Violin plots showing the expression levels of lncRNA‐MEG3 in the differentiation cluster of MuSCs from young and aged mice (*n* = 3). C) qRT‐PCR analysis of lncRNA‐MEG3 expression at different skeletal muscle developmental stages in mice (*n* = 6). (A, B, C, D, and E indicate a highly significant difference). D) Heat map illustrating differentially expressed genes in skeletal muscle tissue from wild‐type (WT) and MDX mice. E) qRT‐PCR showing lncRNA‐MEG3 expression across various tissues (Quadriceps femoris (QUA), Gastrocnemius (GAS), Soleus (SOL), Extensor digitorum longus (EDL), Brain, Stomach, Heart, Brown adipose tissue (BAT), White adipose tissue (WAT), Ovary, Kidney, Lung and Liver) from 6‐week‐old mice (*n* = 3). F) GTEx database analysis shows H3K27ac marks in muscle tissue along the lncRNA‐MEG3 locus, indicating the presence of an enhancer. G) Schematic representation of knockdown strategy using AAV9‐NC or AAV9‐lncRNA‐MEG3 shRNA (AAV9‐KD) injections into neonatal WT mice. Experimental timeline created in Adobe Illustrator. H) qRT‐PCR analysis of lncRNA‐MEG3 expression in muscle tissue from AAV9‐NC or AAV9‐KD injected mice (*n* = 6). I) QUA muscle weight in 8 weeks post‐AAV9‐NC (*n* = 5) or AAV9‐KD (*n* = 6) injections mice. J) QUA myofiber size in 8 weeks post‐AAV9‐NC (*n* = 4) or AAV9‐KD (*n* = 5) injections mice. Scale bar = 100 µm. K) Schematic representation of the experimental design for AAV9 injections and analysis timeline. Adult mice received AAV9 knockdown lncRNA‐MEG3 (AAV9‐KD) or a control vector (AAV9‐NC) followed by cardiotoxin (CTX)‐induced injury on mice TA muscle. Analysis was performed at various time points post‐injury (3.5, 5.5, 10‐, and 25‐days post‐injury (dpi)). L) Representative images of injured and uninjured TA muscles from AAV9‐NC and AAV9‐KD mice (*n* = 6). Scale bar = 0.5 cm. Representative H&E staining M) and immunofluorescence (IF) staining N) of TA muscle cross‐sections (*n* = 3). Scale bar = 50 µm. O) Quantification of eMyHC^+^ myofibers in TA muscle cross‐sections at 5.5 dpi (*n* = 6). P) Quantification of laminin^+^ myofibers in TA muscle cross‐sections at 10 dpi (*n* = 6). Q) Distribution of fiber sizes at 10 dpi from AAV9‐NC and AAV9‐KD mice TA muscle after CTX injury (*n* = 4). R) qRT‐PCR showing the lncRNA‐MEG3, Myhc and Myog expression at 10 dpi after knockdown lncRNA‐MEG3 in TA muscle (*n* = 6). Data are mean ± SD; *p*‐values were calculated using Student's *t*‐test. ^*^ *p* < 0.05, ^**^ *p* < 0.01 and ^***^ *p* < 0.001.

To elucidate the role of lncRNA‐MEG3 in skeletal muscle, we first analyzed the publicly available single‐cell RNA sequencing dataset (GSE147457),^[^ ^22^ ^]^ (GSM4732632)^[^ ^23^ ^]^ and Human Muscle Ageing Cell Atlas database (https://db.cngb.org/cdcp/hlma/rnaseq/).^[^ ^24^ ^]^ This dataset includes data from human skeletal muscle development, aging, and the mouse DMD model (MDX). Our analysis revealed that lncRNA‐MEG3 expression gradually increased during embryonic development and juvenile stages, but declined with aging from puberty to old age in muscle stem cells (MuSCs) (**Figure** **1**A,B; Figure S1A,B and Table S1, Supporting Information). In mice muscle at various developmental stages (embryonic day 15, birth, 5 weeks, 10 months, and 18 months), we observed a similar trend with lncRNA‐MEG3 expression first rising and then decreasing over time (Figure 1C). Additionally, we found that lncRNA‐MEG3 expression in MuSCs from the MDX mouse model was lower, compared with wild‐type (WT) controls (Figure 1D; Figure S2A, Supporting Information). Importantly, lncRNA‐MEG3 was highly expressed in slow‐twitch muscle fibers (Figure 1E). In human tissues from the Genotype‐Tissue Expression (GTEx) project, we identified a potential enhancer within the lncRNA‐MEG3 locus in muscle tissue (Figure 1F). Collectively, these findings establish lncRNA‐MEG3 as a highly enriched non coding RNA regulator, with potentially significant roles in skeletal muscle identity and disease.

### 2.2 LncRNA‐MEG3 is Essential for Muscle Mass Maintenance and Regeneration

To investigate the functional role of lncRNA‐MEG3, we examined its influence on skeletal muscle growth during postnatal development by extensively inhibiting lncRNA‐MEG3 expression. This was achieved through the intraperitoneal injection of adenoviral‐associated viral (AAV) vectors carrying shRNA targeting lncRNA‐MEG3 (AAV9‐KD) or a control vector (AAV9‐NC) into postnatal day 3 mice (Figure 1G). After 8 weeks of AAV delivery, we confirmed efficient knockdown of lncRNA‐MEG3 in skeletal muscles (Figure 1H; Figure S3A,B, Supporting Information). Notably, the reduction of lncRNA‐MEG3 levels resulted in a significant decrease in muscle weight (Figure 1I; Figure S3C,D, Supporting Information), with further validation of atrophic changes at the myofiber level (Figure 1J). While muscle atrophy is known to exhibit fiber‐type‐specific responses, systemic delivery of AAV‐shRNA targeting lncRNA‐MEG3 led to a decrease in the cross‐sectional area (CSA) of muscle fibers across all tested muscle groups, suggesting that lncRNA‐MEG3 knockdown has a broad, detrimental effect on muscle mass regulation (Figure S3E,F, Supporting Information). Additionally, lncRNA‐MEG3 knockdown upregulated the expression of well‐established muscle atrophy markers, Atrogin‐1 and Murf1, at both the mRNA and protein levels (Figure S3G,H, Supporting Information). Next, we explored whether lncRNA‐MEG3 could induce spontaneous muscle mass reduction in the absence of external atrophic stimuli. For this, AAV9‐NC and AAV9‐KD viruses were injected into the tibialis anterior (TA) muscle of 5‐month‐old mice (Figure S3I, Supporting Information). After 8 weeks of AAV‐mediated delivery, efficient knockdown of lncRNA‐MEG3 was confirmed in the TA muscle (Figure S3J, Supporting Information). Lower expression of lncRNA‐MEG3 in these muscles was associated with a trend toward reduced TA muscle weight (Figure S3K, Supporting Information) and a significant decrease in muscle fiber size (Figure S3L,M, Supporting Information). Furthermore, we investigated the impact of lncRNA‐MEG3 knockdown on muscle mass using a mice model of cardiotoxin (CTX) induced muscle injury (Figure 1K). At 5.5 days post‐injury (dpi), lncRNA‐MEG3 knockdown significantly impaired muscle recovery compared to WT controls (Figure 1L). Immunohistochemical analysis further confirmed a substantial accumulation of inflammatory myofibers in the muscles of lncRNA‐MEG3 knockdown mice (Figure S3N,O, Supporting Information). Notably, AAV9 injection for 2 weeks did not significantly affect muscle size or weight, ensuring that the observed effects were specifically attributed to lncRNA‐MEG3 depletion (Figure S3P, Supporting Information). Histological analysis using hematoxylin and eosin (H&E) staining further revealed a smaller regenerative area in lncRNA‐MEG3 knockdown muscles at 3.5, 5.5‐, and 10‐ dpi, with impaired muscle repair (Figure 1M). IF staining further confirmed that lncRNA‐MEG3 knockdown compromised muscle regeneration, as evidenced by a decrease in the number of newly regenerated (eMyHC^+^) and mature (laminin^+^) muscle fibers at both early (5.5 dpi) and late (10 dpi) stages of recovery (Figure 1N–P). At 25 dpi, the distribution of the CSA in muscle fibers in the lncRNA‐MEG3 knockdown group significantly decreased, accompanied by a reduction in the expression of differentiation marker genes (Myog and Myhc) (Figure 1Q,R). Together, these findings establish that lncRNA‐MEG3 is a crucial regulator of muscle mass.

### 2.3 LncRNA‐MEG3 Drives Skeletal Muscle Mass and Function Through Modulation of Muscle Fiber Composition and Lipid Deposition

**Figure 2:**
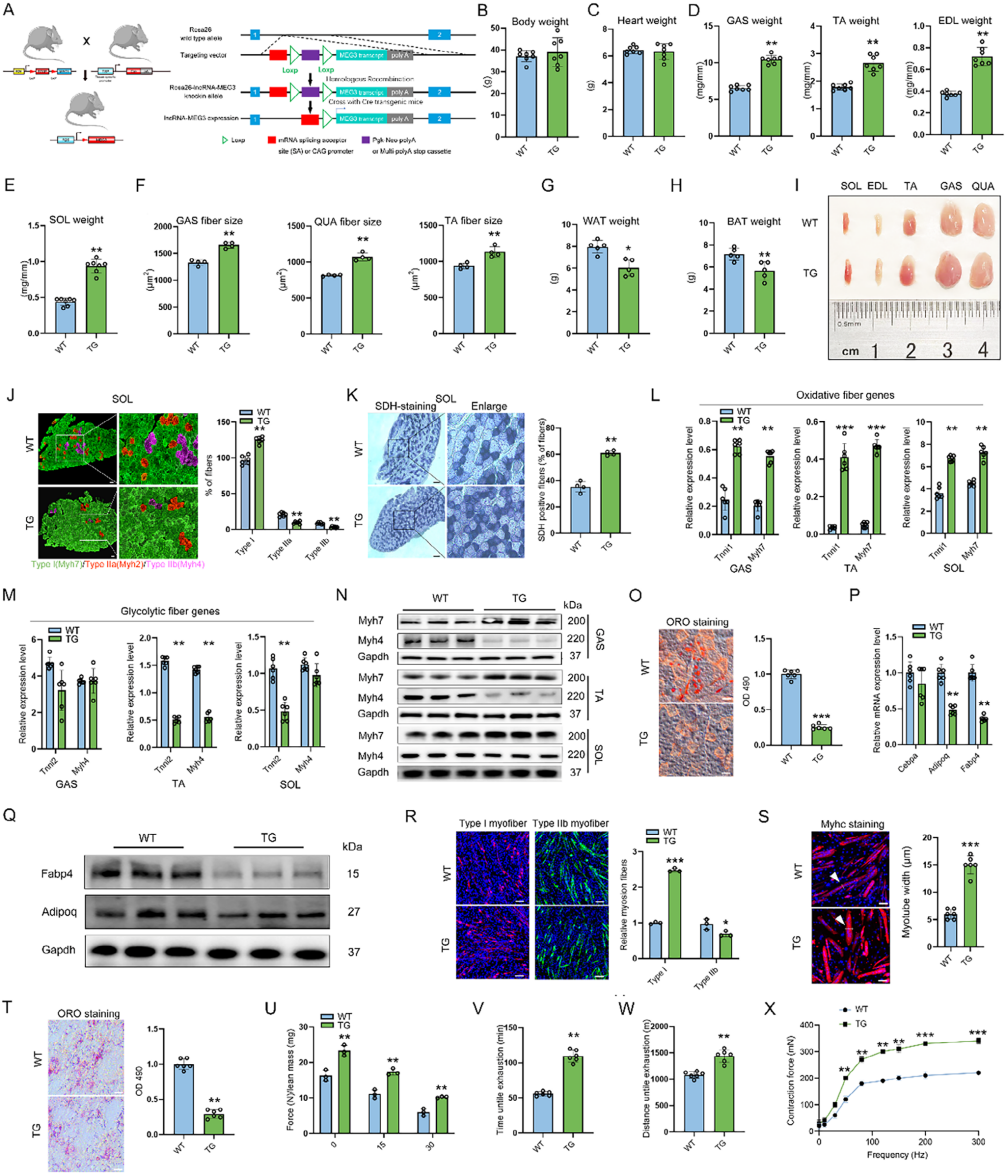
LncRNA‐MEG3 TG mice maintain muscle mass and promote an increase in slow‐twitch muscle fibers. A) Schematic illustrating the experimental design for TG mice with lncRNA‐MEG3 overexpression, generated through Cre‐LoxP‐mediated homologous recombination at the Rosa26 locus to induce lncRNA‐MEG3 expression. B) Body weight, C) Heart weight, D) GAS weight, TA weight, EDL weight, and E) SOL weight in wild‐type (WT) and lncRNA‐MEG3 TG mice (*n* = 7). All weights were normalized to tibia length (TL) to account for differences in body size WT and TG. F) Fiber size of GAS, TA, and QUA muscles in WT and lncRNA‐MEG3 TG mice (*n* = 4). G,H) WAT (G) and BAT weight (H) in WT and lncRNA‐MEG3 TG mice (*n* = 5). I) Representative images of skeletal muscle (SOL, GAS, EDL, QUA, and TA) from 6‐month‐old WT and TG mice (*n* = 3). Scale bar = 0.5 cm. J) Representative Myh7, Myh4, and Myh2 immunostaining in SOL muscles from WT and lncRNA‐MEG3 TG mice, with quantification of staining intensity using ImageJ (*n* = 3). Quantification is shown (right panel). Scale bar = 200 µm. K) Representative SDH staining of SOL muscle sections from WT and lncRNA‐MEG3 TG mice, with quantification of SDH‐positive fibers using ImageJ (*n* = 4). Scale bar = 200 µm. L–N) qRT‐PCR (*n* = 6) and Western blot (*n* = 3) analyses showing the expression of oxidative and glycolytic genes relative to marker genes in GAS, TA, and SOL muscles from WT and lncRNA‐MEG3 TG mice. O) Representative Oil Red O (ORO) staining of lipid droplets in GAS muscle tissue from WT and lncRNA‐MEG3 TG mice (*n* = 6). Scale bar = 50 µm. P,Q) qRT‐PCR (*n* = 6) and Western blot (*n* = 3) analysis of adipogenesis‐related gene expression in GAS muscle tissue from WT and lncRNA‐MEG3 TG mice. R) Representative Myh7 and Myh4 immunostaining in primary myotubes derived from WT and lncRNA‐MEG3 TG mice (*n* = 3). Scale bar = 100 µm. S) Representative Myhc staining and quantification of myotube width in primary myotubes from WT and lncRNA‐MEG3 TG mice (*n* = 6). Scale bar = 50 µm. T) Representative ORO staining of lipid droplets in primary myotubes from WT and lncRNA‐MEG3 TG mice (*n* = 6). Scale bar = 50 µm. U–W) Forelimb grip strength measurements (*n* = 3) (U) and treadmill endurance performance, including running time (V) and distance (W) in WT and lncRNA‐MEG3 TG mice (*n* = 6). Forelimb grip strength was measured at 15 min intervals and standardized to lean body weight. (X) The contraction force was determined by triggering contraction using incremental stimulation frequencies (1 ms pulses at 0–300 Hz for 500 ms at 100 V) (*n* = 3). Data are mean ± SD; *p*‐values were calculated using Student's *t*‐test. ^*^ *p* < 0.05, ^**^ *p* < 0.01 and ^***^ *p* < 0.001.

To assess whether lncRNA‐MEG3 is sufficient to drive muscle mass, we utilized the Cre‐LoxP system for the in vivo overexpression of lncRNA‐MEG3 (**Figure** **2**A). While there was no significant impact on body or heart weight (Figure 2B,C), chronic overexpression of lncRNA‐MEG3 resulted in an increase in the mass of all skeletal muscles analyzed in 8‐month‐old mice (Figure 2D,E; Figure S3Q, Supporting Information). Quantification of muscle fibers CSA in the TA, GAS, and QUA confirmed the observed increase in muscle mass (Figure 2F). Moreover, we found that compared with WT mice, the mass of both white and brown adipose tissue was reduced in lncRNA‐MEG3 transgenic (TG) mice (Figure 2G,H). The muscle phenotype driven by lncRNA‐MEG3 was progressive, as a mild phenotype was observed in 6‐month‐old mice (Figure S4A–H, Supporting Information), with no significant atrophy detected following 5 weeks of overexpression (Figure S4I–P, Supporting Information). Notably, in aging mice, overexpression of lncRNA‐MEG3 not only increased skeletal muscle mass but also led to an increase in overall body weight (Figure S4Q–W, Supporting Information). However, the effect on adipose tissue was no longer evident (Figure S4X, Supporting Information). We hypothesize that in younger mice, lncRNA‐MEG3 may influence systemic metabolism, while the age‐related decline in metabolic capacity may account for the diminished impact on fat deposition in older animals. So, we further investigated the impact of lncRNA‐MEG3 on different muscle fiber types and muscle lipid deposition. Upon visual inspection, each of the different types of muscles from the lncRNA‐MEG3 mice was redder in color compared with WT mice (Figure 2I). To assess muscle fiber types, we performed immunofluorescent staining for myosin heavy chain (Myhc) isoforms in the SOL muscles. The results showed that the overexpression of lncRNA‐MEG3 led to an increased density of oxidative fibers and a reduced number of glycolytic fibers, in contrast to that observed in the WT mice (Figure 2J). When muscle sections were immune‐stained for succinate dehydrogenase (SDH), a marker of mitochondrial activity, less than 38% of fibers in the muscle of WT mice were SDH‐positive, whereas over 60% of myofibers in lncRNA‐MEG3 mice exhibited SDH positivity (Figure 2K). Similar changes were observed in the GAS and TA muscles (Figure S5A,B, Supporting Information). Slow fiber‐specific genes were significantly upregulated in the TG mice at both mRNA and protein levels, while fast fiber‐specific genes were downregulated (Figure 2L–N). Oil Red O (ORO) staining of the GAS muscle cross‐sections indicated a trend toward reduced lipid droplet numbers (Figure 2O). Additionally, the expression of adipogenesis marker genes (Adipoq and Fabp4) was reduced at both the mRNA and protein levels in lncRNA‐MEG3 TG mice (Figure 2P,Q). Importantly, lncRNA‐MEG3 TG mice exhibited accelerated muscle regeneration and an increased proportion of slow muscle fibers following CTX‐induced damage to the TA muscle (Figure S6A–O, Supporting Information). Additionally, lncRNA‐MEG3 was positively correlated with Myh7 (*R* = 0.52, *P* = 0.00071), but had no correlation with Myh4 (*R* = 0.17, *P* = 0.53) during human skeletal muscle development (Figure S6P,Q, Supporting Information). Further analysis of primary myoblasts isolated from lncRNA‐MEG3 TG mice revealed that lncRNA‐MEG3 overexpression resulted in significantly increased type I fibers, enlarged myotube sizes, and reduced adipogenesis in myotubes (Figure 2R–T). To determine the functional consequences of the observed muscle atrophy after 8 months of lncRNA‐MEG3 deficiency, we performed grip strength measurements, which demonstrated that lncRNA‐MEG3 overexpression enhanced muscle strength (Figure 2U). Additionally, lncRNA‐MEG3 TG mice showed improved endurance in treadmill tests, as evidenced by increased running time and running distance (Figure 2V,W). Furthermore, we observed an increase in muscle contraction force in lncRNA‐MEG3 TG mice, compared with the WT control (Figure 2X). In summary, these data suggest that lncRNA‐MEG3 is essential for promoting the accumulation of slow muscle fibers, maintaining muscle mass, and preserving muscle function over time.

### 2.4 Elevated Expression of LncRNA‐MEG3 Alleviates Muscle Atrophy Induced by Fat Infiltration

**Figure 3:**
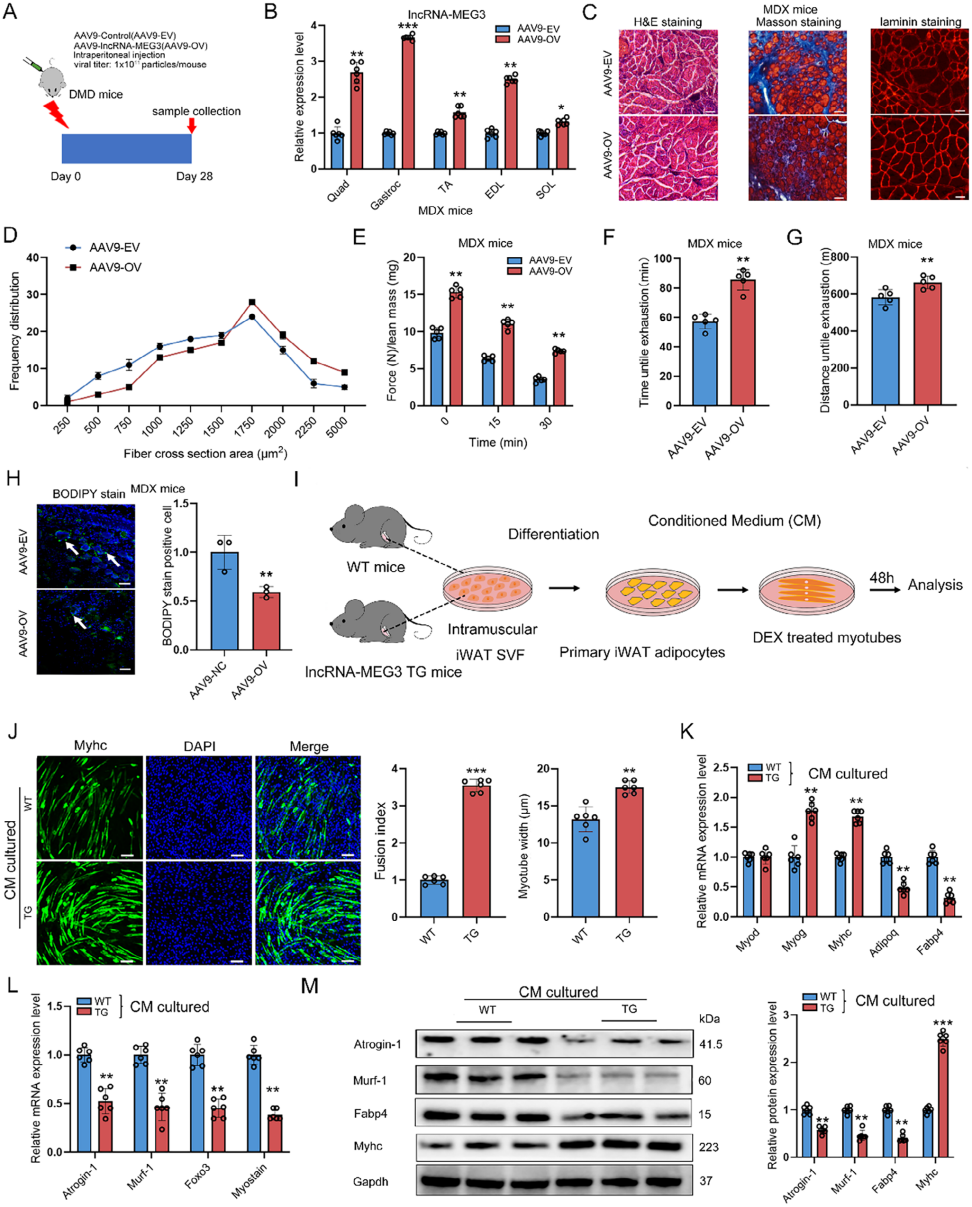
LncRNA‐MEG3 regulates muscle atrophy induced by fat infiltration. A) Schematic of the experimental design using AAV9‐based overexpression (AAV9‐OV) in MDX mice. B) Expression of lncRNA‐MEG3 in QUA, GAS, TA, EDL, and SOL muscles after AAV9‐OV injection in MDX mice (*n* = 6). C) Representative H&E, Masson, and Laminin staining of muscle cross‐sections showing structural changes following AAV9‐OV in MDX mice (*n* = 6). scale bar = 50 µm. D) Distribution of myofiber sizes in AAV9‐EV and AAV9‐OV MDX mice (*n* = 6). E–G) Forelimb grip strength treadmill endurance performance in MDX mice treated with AAV9‐EV or AAV9‐OV (*n* = 5). (H) Representative BODIPY staining of lipid droplets in muscle tissues and quantification of lipid number (*n* = 3). scale bar = 50 µm. I) Schematic illustration of the co‐culture system of beige adipocytes and myotubes. J) Representative images of Myhc staining and myotube width in DEX‐treated C2C12 myotubes cultured with or without a conditioned medium (CM) from differentiated primary beige adipocytes of WT and lncRNA‐MEG3 TG mice (*n* = 6). Scale bar = 100 µm. K–M) qRT‐PCR and Western blot showing the expression of adipogenesis, muscle atrophy, and differentiation marker genes in DEX‐treated C2C12 myotubes cultured with or without CM from differentiated primary beige adipocytes of WT and lncRNA‐MEG3 TG mice (*n* = 6). CM: Conditioned medium, DEX: Dexamethasone. Data are mean ± SD; *p*‐values were calculated using Student's *t*‐test. ^*^ *p* < 0.05, ^**^ *p* < 0.01 and ^***^ *p* < 0.001.

Given that the overexpression of lncRNA‐MEG3 in adult mice resulted in increased muscle mass, we sought to investigate whether modulation of lncRNA‐MEG3 levels could influence the pathophysiology of muscle‐wasting diseases. In a model of DMD, we observed significant muscle fiber atrophy and intramuscular fat infiltration (Figure S7A–D, Supporting Information). Grip strength and endurance were significantly reduced in MDX mice compared with WT (Figure S7E–G, Supporting Information). Moreover, lncRNA‐MEG3 expression was markedly decreased, while the expression of muscle atrophy markers Atrogin‐1 and Murf‐1 was significantly elevated in the TA, SOL, and GAS muscles of MDX mice (Figure S7H, Supporting Information). Similarly, after 48 h of fasting in C57BL/6 mice, which resulted in reduced muscle mass and fiber size, lncRNA‐MEG3 expression decreased in atrophied muscle tissue (Figure S7I–K, Supporting Information). These data underscore a close relationship between lncRNA‐MEG3 expression and muscle atrophy. To explore the therapeutic potential of lncRNA‐MEG3 in muscle wasting, we administered an intraperitoneal injection of the AAV9 virus to induce lncRNA‐MEG3 overexpression in MDX mice (**Figure** **3**A). The overexpression of lncRNA‐MEG3 significantly reduced the number of centrally nucleated fibers, and muscle fibrosis and increased the CSA of muscle fibers (Figure 3B–D). Additionally, lncRNA‐MEG3 overexpression notably improved grip strength and endurance (Figure 3E–G). In primary myoblasts derived from lncRNA‐MEG3‐overexpressing MDX mice, lncRNA‐MEG3 maintains calcium homeostasis by upregulating the expression of the sarco/endoplasmic reticulum Ca^2^⁺‐ATPase (SERCA) gene, further demonstrating its critical role in regulating muscle strength and function in MDX mice (Figure S8A,B, Supporting Information). Importantly, lncRNA‐MEG3 overexpression also reduced lipid accumulation in the muscles of MDX mice (Figure 3H), suggesting its potential therapeutic application in mitigating muscle atrophy associated with intramuscular fat deposition. To confirm the underlying mechanism, we isolated stromal vascular fraction (SVF) cells from the GAS muscles of WT and lncRNA‐MEG3 TG mice and differentiated them into mature adipocytes (Figure 3I). Conditioned media (CM) from these cells was then applied to dexamethasone (DEX)‐induced C2C12 myotubes, a model of muscle atrophy, for morphological and molecular analysis (Figure 3I). As shown in Figure 3J–M, CM from lncRNA‐MEG3 TG mice significantly enhanced myotube size, differentiation capacity, and reduced the expression of muscle atrophy‐related mRNA and protein markers compared with CM from WT cells. These findings suggest that lncRNA‐MEG3 mitigates muscle wasting by preventing fat infiltration. Additionally, it is well‐established that fat infiltration can trigger inflammatory responses and oxidative stress.^[^ ^25^ ^]^ In primary myoblasts isolated from lncRNA‐MEG3‐overexpressing MDX mice, the expression of antioxidant enzymes Sod1 and Cat was significantly upregulated, while the levels of pro‐inflammatory cytokines IL‐6 and IL‐1β were notably decreased (Figure S8B, Supporting Information). Moreover, the production of reactive oxygen species and IL‐6 was significantly reduced after lncRNA‐MEG3 overexpression in MDX mice (Figure S8C,D, Supporting Information). Collectively, these results indicate that lncRNA‐MEG3 attenuates fat infiltration‐induced oxidative stress and inflammatory responses, highlighting its therapeutic potential in treating muscle degenerative diseases such as DMD.

### 2.5 LncRNA‐MEG3 Regulates Mitochondrial Content and Lipid Metabolism in Skeletal Muscle

**Figure 4:**
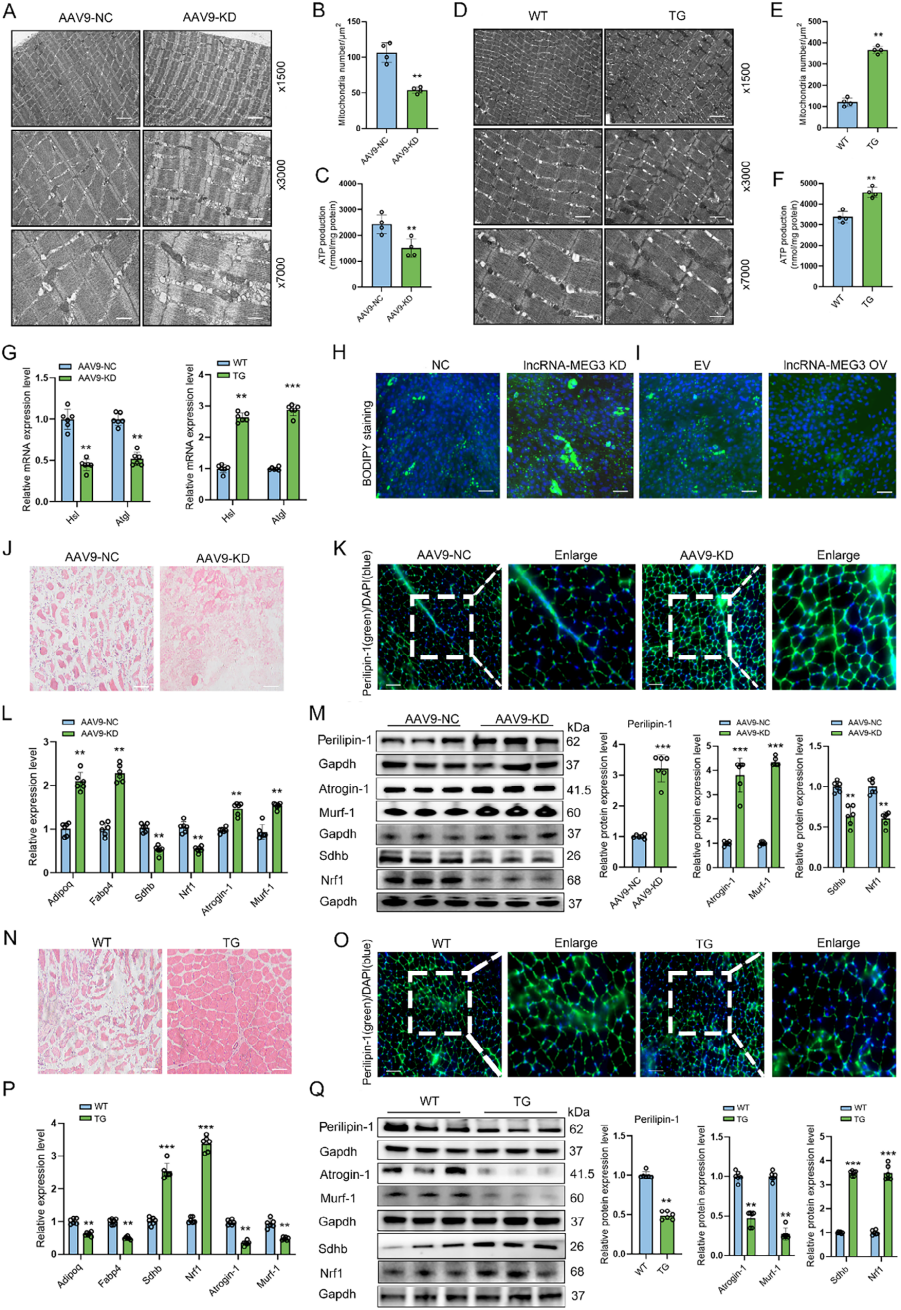
LncRNA‐MEG3 regulates mitochondrial biogenesis impairment induced by fat infiltration. A) Representative transmission electron microscopy (TEM) images of mitochondrial ultrastructure in skeletal muscles from AAV9‐NC and AAV9‐KD mice (*n* = 4). B) Quantification of mitochondrial density per µm^2^ in skeletal muscles in AAV9‐NC and AAV9‐KD mice (*n* = 4). C) ATP production levels in AAV9‐NC and AAV9‐KD mice GAS muscle tissue (*n* = 4). D) Representative transmission electron microscopy (TEM) images of mitochondrial ultrastructure in GAS muscle tissue from WT and lncRNA‐MEG3 TG mice (*n* = 4). E) Quantification of mitochondrial density per µm^2^ in skeletal muscles in WT and lncRNA‐MEG3 TG mice (*n* = 4). F) ATP production levels in skeletal muscles in WT and TG mice (*n* = 4). G) qRT‐PCR showing the expression of lipid metabolism marker genes in muscles with lncRNA‐MEG3 knockdown or overexpression (*n* = 6). H,I) Representative BODIPY staining in C2C12 myotubes with lncRNA‐MEG3 knockdown or overexpression (*n* = 3). Scale bar = 100 µm. J) H&E staining of TA muscle at the 14 dpi after GLY injury with lncRNA‐MEG3 knockdown (*n* = 3). Scale bar = 100 µm. K) IF analysis of Perilipin‐1 (green) and DAPI (blue) at the 14 dpi after GLY injury in muscles with lncRNA‐MEG3 knockdown (*n* = 3). Scale bar = 100 µm. L,M) qRT‐PCR and Western blot analysis of muscle atrophy, lipid deposition, and mitochondrial biogenesis marker genes in TA muscles at the 14 dpi after GLY injury with lncRNA‐MEG3 knockdown (*n* = 6). N) H&E staining of TA muscle at the 14 dpi after GLY injury in WT and lncRNA‐MEG3 TG mice (*n* = 3). Scale bar = 100 µm. O) IF analysis of Perilipin‐1 (green) and DAPI (blue) at the 14 dpi after GLY injury in lncRNA‐MEG3 TG mice (*n* = 3). Scale bar = 100 µm. P,Q) qRT‐PCR and Western blot analysis of muscle atrophy, lipid deposition, and mitochondrial biogenesis marker genes in TA muscle at the 14 dpi after GLY injury in WT and lncRNA‐MEG3 TG mice (*n* = 6). Data are mean ± SD; *p*‐values were calculated using Student's *t*‐test. ^*^ *p* < 0.05, ^**^ *p* < 0.01 and ^***^ *p* < 0.001.

Electron microscopy analysis of GAS muscle from AAV9‐KD mice revealed a significant reduction in mitochondrial density, accompanied by notable disruption of mitochondrial cristae, compared with WT controls (**Figure** **4**A,B). These findings correlate well with the decreased mitochondrial DNA content and suppressed expression of genes related to mitochondrial biogenesis and oxidative phosphorylation in GAS muscle from lncRNA‐MEG3 KD mice (Figure S9A–C, Supporting Information). To determine whether the decrease in mitochondrial numbers in lncRNA‐MEG3 KD mice was associated with impaired respiratory function, we performed an O2K system analysis. The results showed that lncRNA‐MEG3 KD suppressed ATP production and mitochondrial oxygen consumption rate (OCR) in GAS muscle fibers (Figure S9D–F, Supporting Information). Mitochondrial ATP synthesis relies on the electron transport chain and oxidative phosphorylation.^[^ ^26^ ^]^ After stimulation with carbonyl cyanide 4‐(trifluoromethoxy) phenylhydrazone (FCCP), both basal respirations, following oligomycin inhibition of ATP synthesis, and maximal respiration, were significantly lower in GAS muscle fibers from lncRNA‐MEG3 KD mice compared with control (Figure S9E,F, Supporting Information). Similarly, total ATP content was reduced in the GAS muscle of lncRNA‐MEG3 KD mice (Figure 4C), whereas TG mice exhibited the opposite trend (Figure 4D–F; Figure S9G–L, Supporting Information), indicating that lncRNA‐MEG3 plays a crucial role in maintaining mitochondrial function. Furthermore, in isolated intramuscular adipocytes, we observed that the knockdown of lncRNA‐MEG3 suppressed the expression of key lipolysis markers (Hsl and Atgl), while overexpression promoted their expression (Figure 4G). Similarly, in C2C12 myoblasts, lncRNA‐MEG3 knockdown enhanced adipogenesis, whereas its overexpression inhibited this process (Figure 4H,I). These findings indicate that lncRNA‐MEG3 modulates lipid metabolism, leading to altered fat deposition in skeletal muscle. To explore whether the effects of lncRNA‐MEG3 knockdown on lipid accumulation were linked to mitochondrial dysfunction, we employed a glucocorticoid‐induced fat infiltration model in both lncRNA‐MEG3 KD and TG mice. After 14 days of treatment, lncRNA‐MEG3 KD mice showed significant fat infiltration and impaired muscle regeneration in the TA muscle (Figure 4J). Correspondingly, lncRNA‐MEG3 knockdown led to elevated levels of Perilipin‐1 protein (Figure 4K), along with the upregulation of mature adipocyte markers (Adipoq, Fabp4), and muscle atrophy markers, while muscle differentiation and mitochondrial biogenesis markers were suppressed (Figure 4L,M). In contrast, lncRNA‐MEG3 TG mice exhibited reduced lipid accumulation, muscle atrophy markers, and enhanced expression of relevant mitochondrial and muscle differentiation genes (Figure 4N–Q). These results suggest that the loss of lncRNA‐MEG3 promotes muscle atrophy induced by lipid infiltration, likely through the disruption of mitochondrial biogenesis and lipid metabolism.

### 2.6 LncRNA‐MEG3 Stabilizes the PRC2 Complex by Interacting with SUZ12 to Regulate Muscle Phenotype and Mitochondrial Function

**Figure 5:**
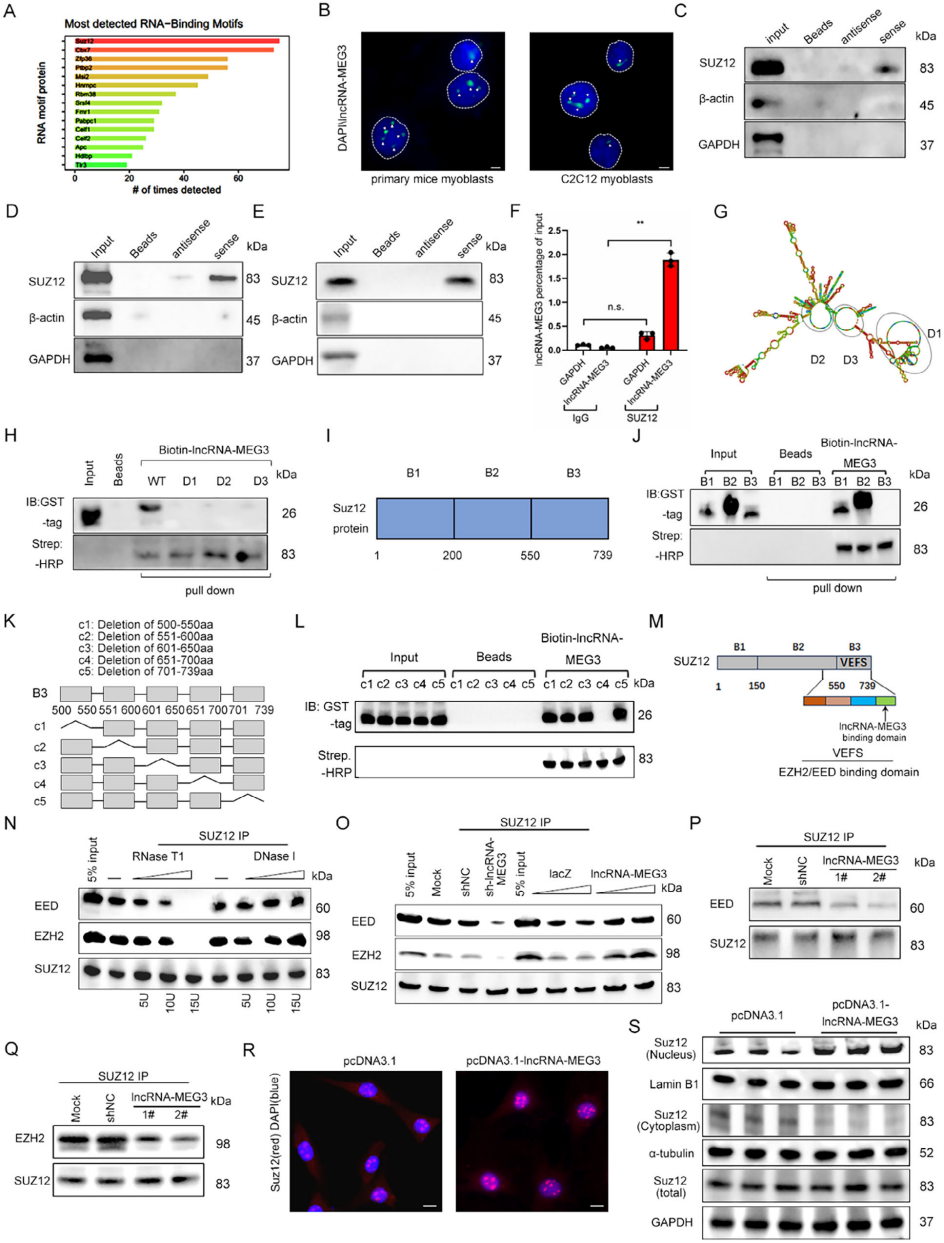
Characterization of the interaction between lncRNA‐MEG3 and SUZ12. A) RNA‐binding motif enrichment analysis showing the most frequently detected RNA‐binding proteins associated with lncRNA‐MEG3 by catRAPID (http://s.tartaglialab.com/page/catrapid_group). B) Fluorescent in situ hybridization (FISH) showing lncRNA‐MEG3 localization (green) in primary mouse myoblasts and C2C12 myoblasts. Nuclei are stained with DAPI (blue) (*n* = 3). Scale bar = 20 µm. C–E) RNA pull‐down assay of SUZ12, β‐actin (negative control), and GAPDH (negative control) using antisense and sense probes for lncRNA‐MEG3 in C2C12 myoblast lysates (*n* = 3). Input represents total lysates. F) Quantification of the lncRNA‐MEG3 interaction with SUZ12 normalized to input level (*n* = 3). G) Predicted secondary structure of lncRNA‐MEG3 highlighting structural domains (D1, D2, and D3). H) RNA pull‐down assay was performed to analyze the binding of lncRNA‐MEG3 WT, deletion expression vectors (D1, D2, and D3), and SUZ12 protein (*n* = 3). I,J) Domain mapping of SUZ12 using truncated fragments (B1, B2, and B3) in pull‐down assays, identifying the lncRNA‐MEG3 interaction domain in SUZ12 (*n* = 3). K) Schematic of the deletion constructs in the B3 domain, with the deleted regions located at the following amino acid positions: c1 (500‐550 aa deletion), c2 (551–600 aa deletion), c3 (601–650 aa deletion), c4 (651–700 aa deletion), and c5 (701–739 aa deletion). L) RNA pull‐down assay showing the interaction between biotinylated lncRNA‐MEG3 and the B3 domain deletion variants (*n* = 3). M) Schematic representation of SUZ12 domains, indicating the lncRNA‐MEG3 binding region and the EZH2/EED interaction region. N) Co‐IP was performed to analyze the interaction between SUZ12 and EZH2/EED after C2C12 myoblasts were treated with RNase T1, DNase I, and lncRNA‐MEG3 shRNA (*n* = 3). O) Co‐IP was performed to analyze the interaction between SUZ12 and EZH2/EED after overexpression of lncRNA‐MEG3 in C2C12 myoblasts (*n* = 3). P,Q) Co‐IP was performed to analyze the interaction between SUZ12 and EZH2/EED in C2C12 myoblasts with knockdown of lncRNA‐MEG3 (*n* = 3). R) IF staining of SUZ12 (red) in control and lncRNA‐MEG3‐overexpressing C2C12 cells showing enhanced nuclear localization. DAPI stains nuclei (blue) (*n* = 3). Scale bar = 20 µm. S) Western blot showing the expression of Suz12 in the nucleus, cytoplasm, and whole cell lysates of C2C12 myoblasts after lncRNA‐MEG3 overexpression (*n* = 3). Lamin B1 was used as a marker for the nuclear protein, α‐Tubulin as a marker for the cytoplasmic protein, and Gapdh as a marker for the total protein. Data are mean ± SD; *p*‐values were calculated using Student's *t*‐test. ^**^ *p* < 0.01.

To investigate the molecular mechanism underlying the role of lncRNA‐MEG3, we employed the catRAPID (http://s.tartaglialab.com/page/catrapid_group) algorithm to predict potential proteins interacting with lncRNA‐MEG3. SUZ12 emerged as the highest‐ranking predicted protein, and we confirmed that lncRNA‐MEG3 is predominantly localized in the nucleus (**Figure** **5**A,B). RNA pull‐down assays in mice primary myoblasts lysates revealed a direct interaction between lncRNA‐MEG3 and SUZ12 (Figure 5C). Notably, similar interactions were observed with lncRNA‐MEG3 from both pigs and humans, suggesting a conserved mechanism across species (Figure 5D,E). RNA immunoprecipitation (RIP) experiments in C2C12 myoblasts further validated the endogenous interaction between lncRNA‐MEG3 and SUZ12 (Figure 5F). To understand the structural basis for this interaction, we predicted the secondary structure of lncRNA‐MEG3, which revealed the presence of three distinct loops (Figure 5G). To identify the specific RNA domains involved in the interaction with SUZ12, we generated a series of lncRNA‐MEG3 mutants by deleting different loop regions. In vitro, RNA‐protein binding assays demonstrated that only the full‐length lncRNA‐MEG3 was able to bind SUZ12, while all mutant versions failed (Figure 5H). Next, we investigated the protein domains of SUZ12 required for its binding to lncRNA‐MEG3 by generating three truncated SUZ12 mutants (B1, B2, and B3) (Figure 5I). The B3 fragment (amino acids 550–739) was identified as the core region essential for binding to lncRNA‐MEG3 (Figure 5J). Further truncation studies narrowed the critical binding domain to the 651–700 amino acid region (Figure 5K,L), which is known to interact with other PRC2 components such as EZH2 and EED (Figure 5M).^[^ ^27^ ^]^ These findings suggest that lncRNA‐MEG3 may modulate the stability of the PRC2 complex, thereby influencing gene regulation. To test this hypothesis, we examined the effects of RNase treatment and lncRNA‐MEG3 knockdown on the interaction between SUZ12, EED, and EZH2. Both treatments led to a loss of these interactions, which could be restored by reintroducing lncRNA‐MEG3 (Figure 5N–Q). Furthermore, the overexpression of lncRNA‐MEG3 promoted the nuclear localization of SUZ12 and facilitated the formation of more PRC2 complexes within the cell (Figure 5R,S). Notably, we found that the knockdown of Suz12 resulted in a marked reduction in type I muscle fibers, increased lipid droplet accumulation, and impaired mitochondrial function (Figure S10A–D, Supporting Information). In addition, Suz12 knockdown rescued the skeletal muscle phenotype induced by lncRNA‐MEG3 overexpression (Figure S10E–H, Supporting Information). Moreover, neither the knockdown nor the overexpression of lncRNA‐MEG3 affected the expression of Suz12 at the total mRNA and protein levels, and Suz12 did not alter lncRNA‐MEG3 expression (Figure 5S; Figure S10I–L, Supporting Information). Together, these results demonstrate that lncRNA‐MEG3 regulates muscle mass, lipid metabolism, and mitochondrial function by stabilizing the PRC2 complex through its interaction with SUZ12.

### 2.7 LncRNA‐MEG3/SUZ12 Maintains H3K27me3 Modification and Regulates Fhl3 and Rnf128 Expression

**Figure 6:**
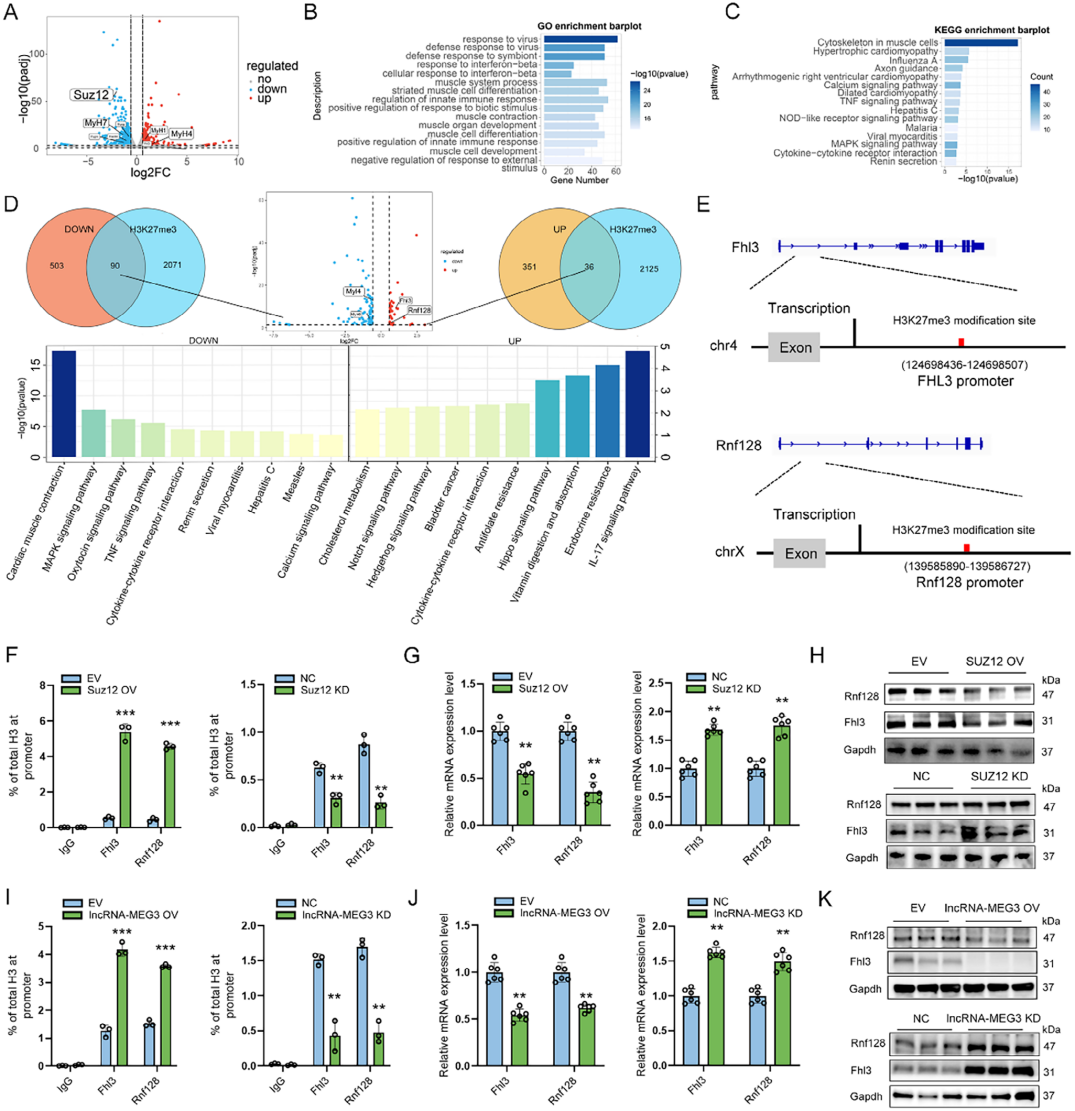
LncRNA‐MEG3 interacts with SUZ12 to regulate the H3K27me3 modification of the Fhl3 and Rnf128 promoter. A) Volcano plot showing differentially expressed genes upon SUZ12 knockdown. B) Gene Ontology (GO) enrichment analysis of DEGs, highlighting biological processes significantly affected by SUZ12 modulation. C) KEGG pathway enrichment analysis of DEGs, showing pathways related to muscle development and immune responses. D) Venn diagrams showing the overlap between DEGs (downregulated and upregulated) and H3K27me3 target genes, with bar plots displaying enriched pathways for overlapping genes. E) Schematic diagrams of Fhl3 and Rnf128 loci, illustrating H3K27me3 modification sites at their promoters. F) ChIP‐qPCR showing the enrichment of H3K27me3 at the Fhl3 promoter in Suz12 knockdown (KD) and overexpression (OV) myoblasts compared to controls (*n* = 3). G,H) qRT‐PCR (*n* = 6) (G) and Western blot (*n* = 3) (H) showing the expression levels of Fhl3 in Suz12 OV and KD myoblast. I) ChIP‐qPCR showing the enrichment of H3K27me3 at the Rnf128 promoter in lncRNA‐MEG3 knockdown (KD) and overexpression (OV) myoblasts compared to controls (*n* = 3). J,K) qRT‐PCR (*n* = 6) (J) and Western blot (*n* = 3) (K) showing the expression levels of Rnf128 in lncRNA‐MEG3 OV and KD myoblast. Data are mean ± SD; P‐values were calculated using Student's *t*‐test. ^*^ *p* < 0.05, ^**^ *p* < 0.01 and ^***^ *p* < 0.001.

To investigate the impact of lncRNA‐MEG3 on downstream target genes through SUZ12, we performed RNA sequencing and H3K27me3 CUT&Tag combined analysis. RNA‐seq analysis revealed that SUZ12 knockdown in myotubes resulted in the differential expression of 980 genes, including the downregulation of Myh7 and upregulation of Myh4 (**Figure** **6**A; Table S2, Supporting Information). Gene Ontology (GO) and Kyoto Encyclopedia of Genes and Genomes (KEGG) enrichment analyses identified significant pathways related to muscle fiber type conversion and energy metabolism, such as calcium signaling and MAPK signaling pathways (Figure 6B,C; Tables S3 and S4, Supporting Information). CUT&Tag analysis following SUZ12 knockdown showed alterations in H3K27me3 modifications at the 2163 genes promoter (Table S5, Supporting Information). Overlap analysis revealed that the genes with altered expression levels also overlapped with those regulated by H3K27me3 (Figure 6D; Table S6, Supporting Information). These overlapping genes were enriched in signaling pathways associated with myogenesis, muscle contraction, and skeletal muscle development, including MAPK, PI3K‐Akt, Hippo, and calcium signaling pathways (Figure 6D; Tables S7 and S8, Supporting Information). Notably, we identified Fhl3 and Rnf128 within the list of differentially enriched genes, and the H3K27me3 modification in their promoter regions is influenced by SUZ12 (Figure 6E). Previous studies have shown that Fhl3 promotes fast‐twitch fiber formation, while Rnf128 promotes fat accumulation.^[^ ^28^, ^29^ ^]^ ChIP‐qPCR, qRT‐PCR, and Western blot results indicated that knockdown of Suz12 and lncRNA‐MEG3 reduced H3K27me3 enrichment at the promoter regions of Fhl3 and Rnf128, leading to their upregulation, while Suz12 and lncRNA‐MEG3 overexpression had the opposite effect (Figure 6F–K). Moreover, Suz12 knockdown was able to rescue the effects of lncRNA‐MEG3 on the expression of both Fhl3 and Rnf128 (Figure S10M, N, Supporting Information). These results indicate that lncRNA‐MEG3 interacts with SUZ12 to regulate the expression of Fhl3 and Rnf128 by modulating H3K27me3 deposition at their promoter regions.

### 2.8 LLPS Determines the Activity of SUZ12

**Figure 7:**
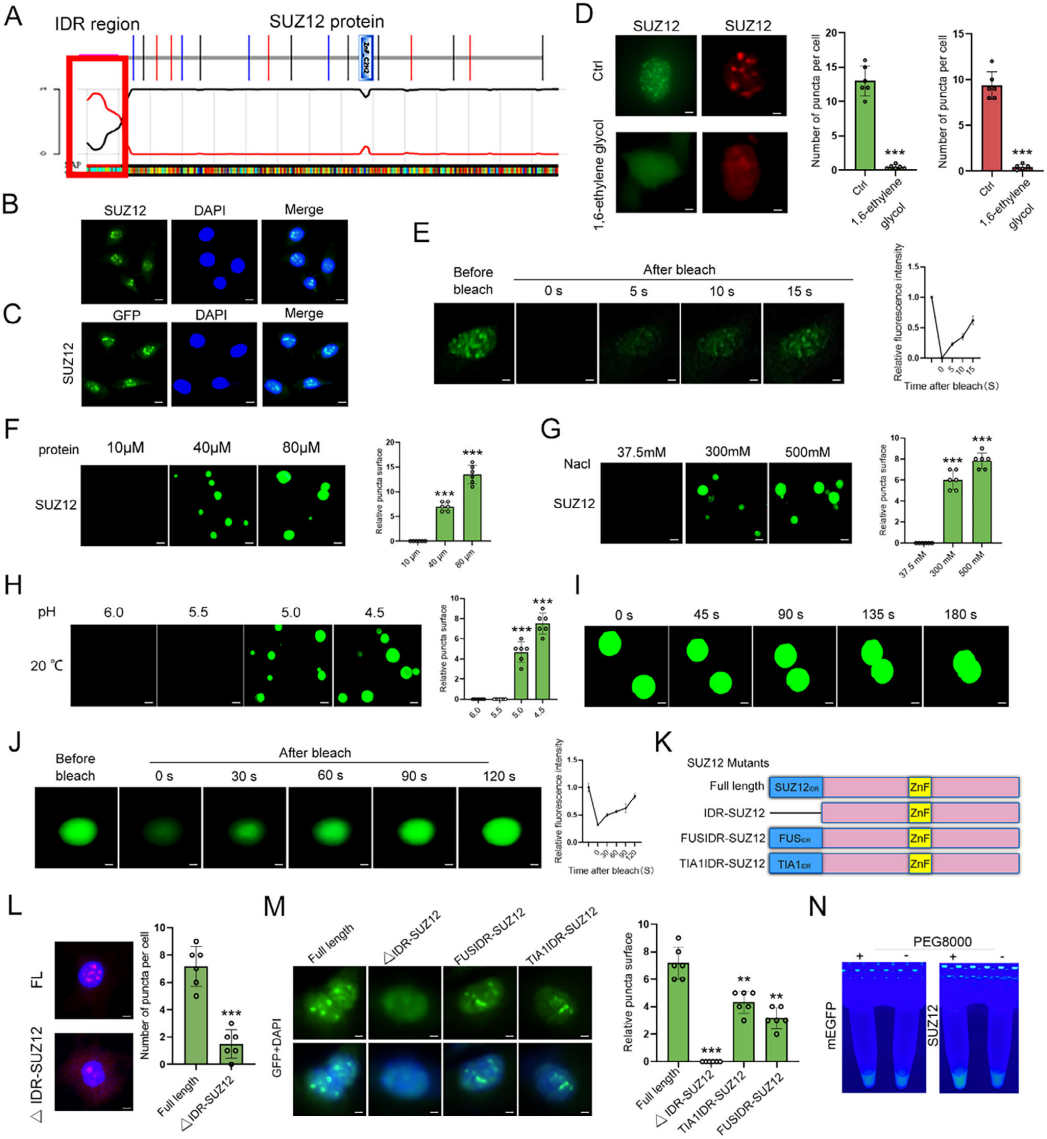
SUZ12 undergoes LLPS. A) Identification of IDR within the SUZ12 protein using bioinformatics analysis. The IDR region (red box) and zinc finger (ZnF) domains are highlighted. B) IF showing SUZ12 (green) localization in C2C12 myoblasts. Nuclei are stained with DAPI (blue) (*n* = 3). Scale bar = 20 µm. C) GFP‐tagged SUZ12 exhibits nuclear localization in C2C12 myoblasts. DAPI stains nuclei (*n* = 3). Scale bar = 20 µm. D) Immunostaining of SUZ12 in C2C12 myoblasts treated with 1,6‐ethylene glycol, showing disrupted puncta formation. Quantification of SUZ12‐positive nuclear puncta per cell is shown (right panel) (*n* = 6). Scale bar = 10 µm. E) Fluorescence recovery after photobleaching (FRAP) of SUZ12 puncta in C2C12 myoblasts, demonstrating its liquid‐like properties. Representative images before and after bleaching are shown with recovery kinetics (right panel). Scale bar = 10 µm. F) Concentration‐dependent phase separation of purified SUZ12 protein in vitro. Representative images and quantification of puncta formation at increasing protein concentrations are shown (*n* = 6). Scale bar = 10 µm. G) Salt sensitivity assay showing SUZ12 puncta disruption with increasing NaCl concentrations (300 to 500 mm) (*n* = 6). Scale bar = 10 µm. H) pH‐dependent phase separation of SUZ12, showing optimal droplet formation at pH 6.0–6.5. Quantification of puncta is shown (right panel) (*n* = 6). Scale bar = 10 µm. I) FRAP analysis of SUZ12 droplets in vitro, demonstrating rapid fluorescence recovery after photobleaching, indicative of liquid‐like dynamics. Scale bar = 10 µm. J) Time‐lapse imaging of SUZ12 droplets coalescence in vitro, showing fusion events over time. K) Schematic of SUZ12 mutants (ΔIDR‐SUZ12, FUSIDR, and TIA1IDR) used to investigate the role of IDR in LLPS. L) IF of full‐length (FL) SUZ12 and ΔIDR‐SUZ12 in C2C12 myoblasts. ΔIDR‐SUZ12 lacks nuclear puncta formation. Quantification is shown (right panel) (*n* = 6). Scale bar = 10 µm. M) Representative images of GFP‐tagged SUZ12 different vectors in C2C12 myoblasts. Quantification of the puncta number is shown in the right panel (*n* = 6). Scale bar = 10 µm. N) Turbidity assay of purified SUZ12 protein in the presence of PEG8000, confirming its ability to undergo phase separation (*n* = 6). Data are mean ± SD; *p*‐values were calculated using Student's *t*‐test. ^*^ *p* < 0.05, ^**^ *p* < 0.01 and ^***^ *p* < 0.001.

To explore the molecular mechanism underlying the influence of SUZ12 on Fhl3 and Rnf128, we analyzed the protein structure of SUZ12. Structural analysis revealed a prominent IDR at the N‐terminus of SUZ12 (**Figure** **7**A), suggesting that SUZ12 may function through LLPS.^[^ ^30^ ^]^ Both IF staining and GFP‐tagged fluorescence imaging confirmed that SUZ12 forms dynamic puncta, a hallmark feature of LLPS (Figure 7B,C). Treatment with 1,6‐ethylene glycol, a known inhibitor of phase separation, prevented the formation of these puncta (Figure 7D), further supporting the LLPS behavior of SUZ12. Photobleaching recovery experiments revealed that SUZ12‐GFP puncta exhibited fluorescence recovery, indicating the dynamic and reversible nature of the phase‐separated structures (Figure 7E). We further examined the effect of various factors on SUZ12 LLPS by manipulating protein concentration, salt ion concentration, and pH. Increasing the concentration of purified SUZ12‐GFP proteins or adjusting ionic conditions significantly enhanced both the size and number of puncta (Figure 7F–H). Moreover, following photobleaching, the purified SUZ12‐GFP proteins exhibited recovery and fusion into larger puncta, reinforcing the dynamic properties of SUZ12 phase separation (Figure 7I,J). These findings collectively demonstrate that SUZ12 exhibits LLPS properties both in vivo and in vitro. To assess the role of the IDR in SUZ12‐mediated LLPS, we constructed IDR‐replacement vectors using IDRs from FUS and TIA1 proteins to substitute for the IDR region in SUZ12‐GFP proteins (Figure 7K), resulting in FUSIDR‐SUZ12 and TIA1IDR‐SUZ12. The deletion of the IDR from SUZ12‐GFP (ΔIDR‐SUZ12) markedly reduced puncta formation, underscoring its essential role in LLPS (Figure 7L). Consistently, purified SUZ12‐GFP proteins lacking the IDR failed to form condensates, whereas the IDR‐replacement variants retained LLPS functionality (Figure 7M). Furthermore, the phase‐separation enhancer PEG8000 reinforced the necessity of the IDR in facilitating SUZ12 LLPS (Figure 7N). Collectively, these findings indicate that LLPS serves as a critical regulatory mechanism governing SUZ12 activity, with the IDR playing a pivotal role in this process.

### 2.9 LncRNA‐MEG3 Modulates SUZ12 LLPS and Epigenetic Regulation of Fhl3 and Rnf128

Subcellular fractionation through differential ultracentrifugation was employed to identify proteins involved in LLPS.^[^ ^31^ ^]^ Our analysis revealed that SUZ12 is present in the P20 fraction (pellet collected at 2000 × g), indicating that SUZ12 is part of a protein subset involved in heavy granule formation. Notably, SUZ12 levels in the P20 fraction decreased upon RNase A treatment or following the knockdown of lncRNA‐MEG3 in cells (Figure S11A,B, Supporting Information). In contrast, the overexpression of lncRNA‐MEG3 led to an increase in SUZ12 levels in the same fraction (Figure S11C, Supporting Information). Orthogonal experiments confirmed that SUZ12 underwent LLPS in a dose‐dependent manner in the presence of lncRNA‐MEG3 (Figure S11D, Supporting Information). Further co‐localization studies demonstrated that both lncRNA‐MEG3 and SUZ12 are predominantly localized in the nucleus (Figure S11E, Supporting Information), suggesting that lncRNA‐MEG3 may promote the formation of SUZ12‐containing heavy granules or liquid droplets via direct interaction. To further test this hypothesis, we performed gain and loss of the function experiment on lncRNA‐MEG3 in C2C12 myoblasts to assess its impact on the formation of SUZ12 puncta. The results showed that the overexpression of lncRNA‐MEG3 in C2C12 myoblasts significantly enhanced the formation of SUZ12 puncta, whereas the knockdown of lncRNA‐MEG3 reduced their formation (Figure S11F,G, Supporting Information). Functional assays revealed that the SUZ12 construct lacking the IDR failed to induce H3K27me3 modification at the Fhl3 and Rnf128 promoter and could not regulate their expression (Figure S11H,I, Supporting Information). Conversely, the overexpression of SUZ12 rescued the ability of lncRNA‐MEG3 knockdown to inhibit H3K27me3 modification and up‐regulation of FHL3 and Rnf128, an effect that was abolished when the SUZ12 IDR was deleted (Figure S11J,K, Supporting Information). Together, these results highlight that lncRNA‐MEG3 plays a pivotal role in promoting SUZ12 LLPS and regulating the epigenetic landscape of Fhl3 and Rnf128.

### 2.10 LncRNA‐MEG3 Regulates Muscle Mass and Atrophy by Promoting SUZ12 LLPS to Modulate the Expression of Fhl3 and Rnf128

To further elucidate the role of the lncRNA‐MEG3/SUZ12/Fhl3/Rnf128 signaling axis in regulating skeletal muscle mass and preventing atrophy, we investigated the functional roles of Fhl3 and Rnf128 in skeletal muscle. The overexpression of Fhl3 in myotubes resulted in the upregulation of Myh4 and Murf1 expression, but in the downregulation of Myh7 and Nrf1 expression, compared with control (Figure S12 A,B, Supporting Information). Additionally, Fhl3 overexpression suppressed ATP synthesis and cAMP production, with the opposite effects observed upon Fhl3 knockdown (Figure S12C–H, Supporting Information). In contrast, overexpression of Rnf128 led to an increase in the levels of muscle atrophy and adipogenesis markers, while simultaneously reducing the levels of mitochondrial biogenesis markers in myotubes (Figure S13A,B, Supporting Information). This was accompanied by a decrease in myotube size, ATP synthesis, and cAMP generation (Figure S13C–E, Supporting Information). Conversely, Rnf128 knockdown inhibited this muscle‐wasting phenotypes (Figure S13F–J, Supporting Information), highlighting the collaborative roles of Fhl3 and Rnf128 in regulating muscle mass, fiber‐type conversion, fat deposition, and muscle atrophy.

**Figure 8:**
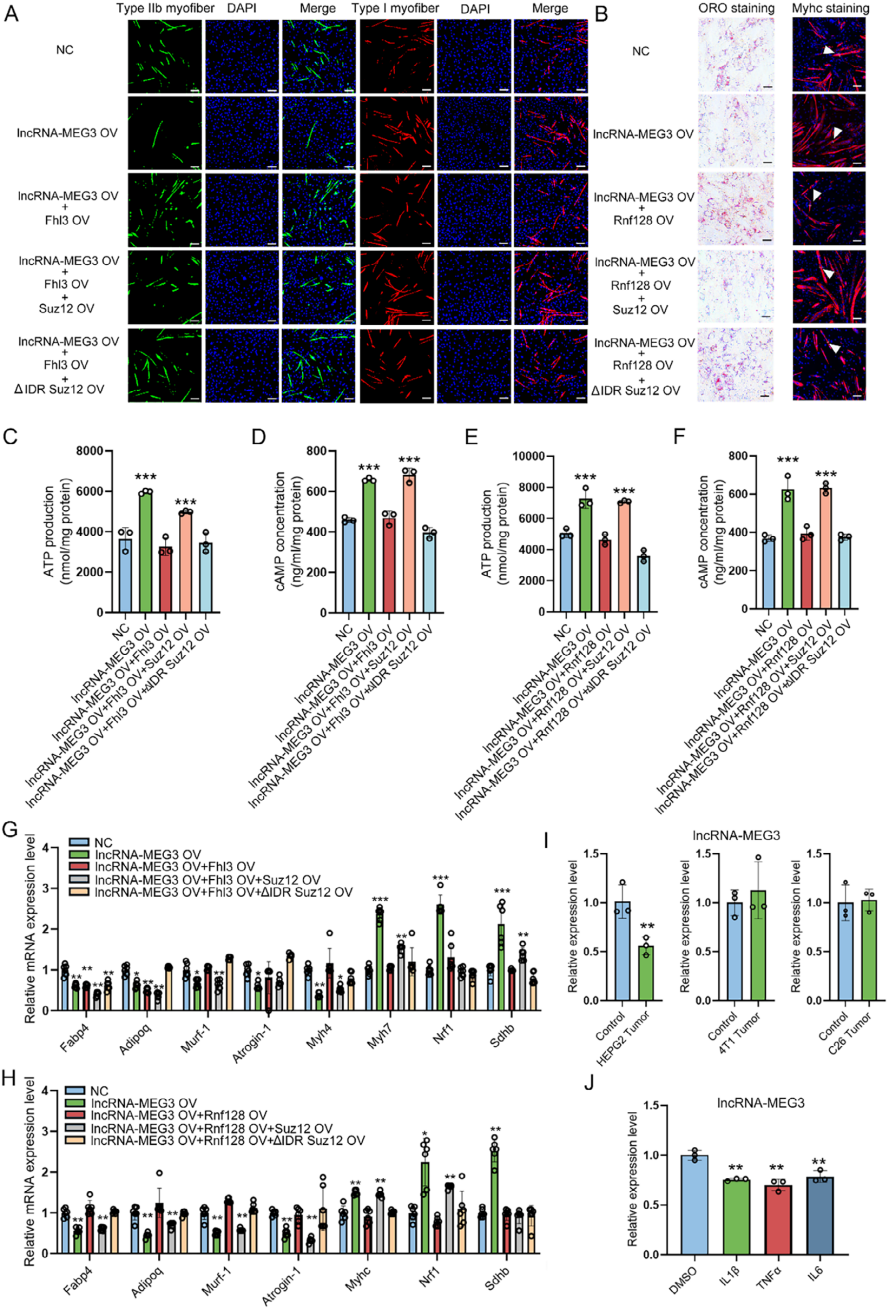
LncRNA‐MEG3 enhances muscle function via SUZ12 LLPS‐mediated regulation of Fhl3 and Suz12. A) Representative immunostaining for Myh7, Myh4 in C2C12 myoblasts after co‐transfected with NC, lncRNA‐MEG3 overexpression vector (lncRNA‐MEG3 OV), lncRNA‐MEG3 OV + Fhl3 overexpression vector (Fhl3 OV), lncRNA‐MEG3 OV + Fhl3 OV + Suz12 overexpression vector (Suz12 OV), lncRNA‐MEG3 OV + Fhl3 OV + Suz12 deleted IDR region overexpression vector (ΔIDR‐Suz12 OV) (*n* = 3). Scale bar = 100 µm. B) Representative ORO staining and immunostaining for Myhc in C2C12 myoblasts after co‐transfected with NC, lncRNA‐MEG3 OV, lncRNA‐MEG3 OV + Rnf128 overexpression vector (Rnf128 OV), lncRNA‐MEG3 OV + Rnf128 OV + Suz12 OV, lncRNA‐MEG3 OV + Rnf128 OV +ΔIDR‐Suz12 OV (*n* = 3). Scale bar = 100 µm. C,D) ATP synthesis and cAMP biogenesis detection after co‐transfected with NC, lncRNA‐MEG3 OV, lncRNA‐MEG3 OV + Fhl3 OV, lncRNA‐MEG3 OV + Fhl3 OV + Suz12 OV, lncRNA‐MEG3 OV + Fhl3 OV +ΔIDR‐Suz12 OV in C2C12 myoblasts (*n* = 3). E,F) ATP synthesis and cAMP biogenesis detection after co‐transfected with NC, lncRNA‐MEG3 OV, lncRNA‐MEG3 OV + Rnf128 OV, lncRNA‐MEG3 OV + Rnf128 OV + Suz12 OV, lncRNA‐MEG3 OV + Rnf128 OV +ΔIDR‐Suz12 OV in C2C12 myoblasts (*n* = 3). G) qRT‐PCR analysis of the expression of marker genes related to muscle atrophy, fat deposition, muscle fiber type conversion, and mitochondrial biogenesis in C2C12 myoblasts after co‐transfected with NC, lncRNA‐MEG3 OV, lncRNA‐MEG3 OV + Fhl3 OV, lncRNA‐MEG3 OV + Fhl3 OV + Suz12 OV, lncRNA‐MEG3 OV + Fhl3 OV +ΔIDR‐Suz12 OV (*n* = 6). (H) qRT‐PCR analysis of marker gene expression related to muscle atrophy, fat deposition, muscle differentiation, and mitochondrial biogenesis in C2C12 myoblasts after co‐transfected with NC, lncRNA‐MEG3 OV, lncRNA‐MEG3 OV + Rnf128 OV, lncRNA‐MEG3 OV + Rnf128 OV + Suz12 OV, lncRNA‐MEG3 OV + Rnf128 OV +ΔIDR‐Suz12 OV (*n* = 6). I) qRT‐PCR showing lncRNA‐MEG3 expression after C2C12 myoblasts treated with culture media for different cancer cell lines (*n* = 3). J) qRT‐PCR showing lncRNA‐MEG3 expression after C2C12 myoblasts treated with different cytokines (*n* = 3). Data are mean ± SD; *p*‐values were calculated using Student's *t*‐test. ^*^ *p* < 0.05, ^**^ *p* < 0.01 and ^***^ *p* < 0.001.

Furthermore, we explored the potential of rescuing the effects of lncRNA‐MEG3 on muscle phenotypes by overexpressing Fhl3 and Rnf128. We found that overexpression of these factors could partially restore muscle atrophy, muscle fiber type transition, mitochondrial biogenesis, fat deposition, and the expression of marker genes under lncRNA‐MEG3‐sufficient conditions (**Figure** **8**A–H). However, when Suz12 was overexpressed in the wild‐type vector, this rescuing effect of Fhl3 and Rnf128 on lncRNA‐MEG3 function was completely abolished (Figure 8A–H). Importantly, transfection with Suz12 lacking its IDR limited the rescue effect, suggesting that the IDR domain of SUZ12 is essential for the regulatory activity of this pathway (Figure 8A–H). These findings underscore the critical role of the lncRNA‐MEG3/SUZ12/Fhl3/Rnf128 axis in maintaining skeletal muscle mass, preventing muscle atrophy, regulating fiber‐type plasticity, and modulating fat deposition. Moreover, SUZ12‐mediated LLPS serves as a key regulatory switch for this signaling cascade.

To investigate the potential impact of cancer‐associated cachexia on muscle atrophy, we exposed differentiated C2C12 myotubes to acellular conditioned media derived from various cancer cell lines known to induce muscle wasting, including 4T1 (breast cancer), C26 (colon cancer), and HEPG2 (liver cancer). Notably, we observed a significant reduction in lncRNA‐MEG3 levels in conditioned media from HepG2 cells (Figure 8I). To further identify the signaling molecules regulating lncRNA‐MEG3 expression in muscle cells, we treated differentiated C2C12 myotubes with cytokines commonly associated with cancer cachexia. We found that pro‐inflammatory cytokines, such as interleukin IL‐6, IL‐1β, and tumor necrosis factor (TNF), significantly downregulated lncRNA‐MEG3 expression (Figure 8J). These results suggest that lncRNA‐MEG3 expression is tightly regulated by circulating cytokines and growth factors, positioning lncRNA‐MEG3 as a crucial mediator in muscle loss during cachexia and cancer‐related wasting.

## 3 Discussion

**Figure 9:**
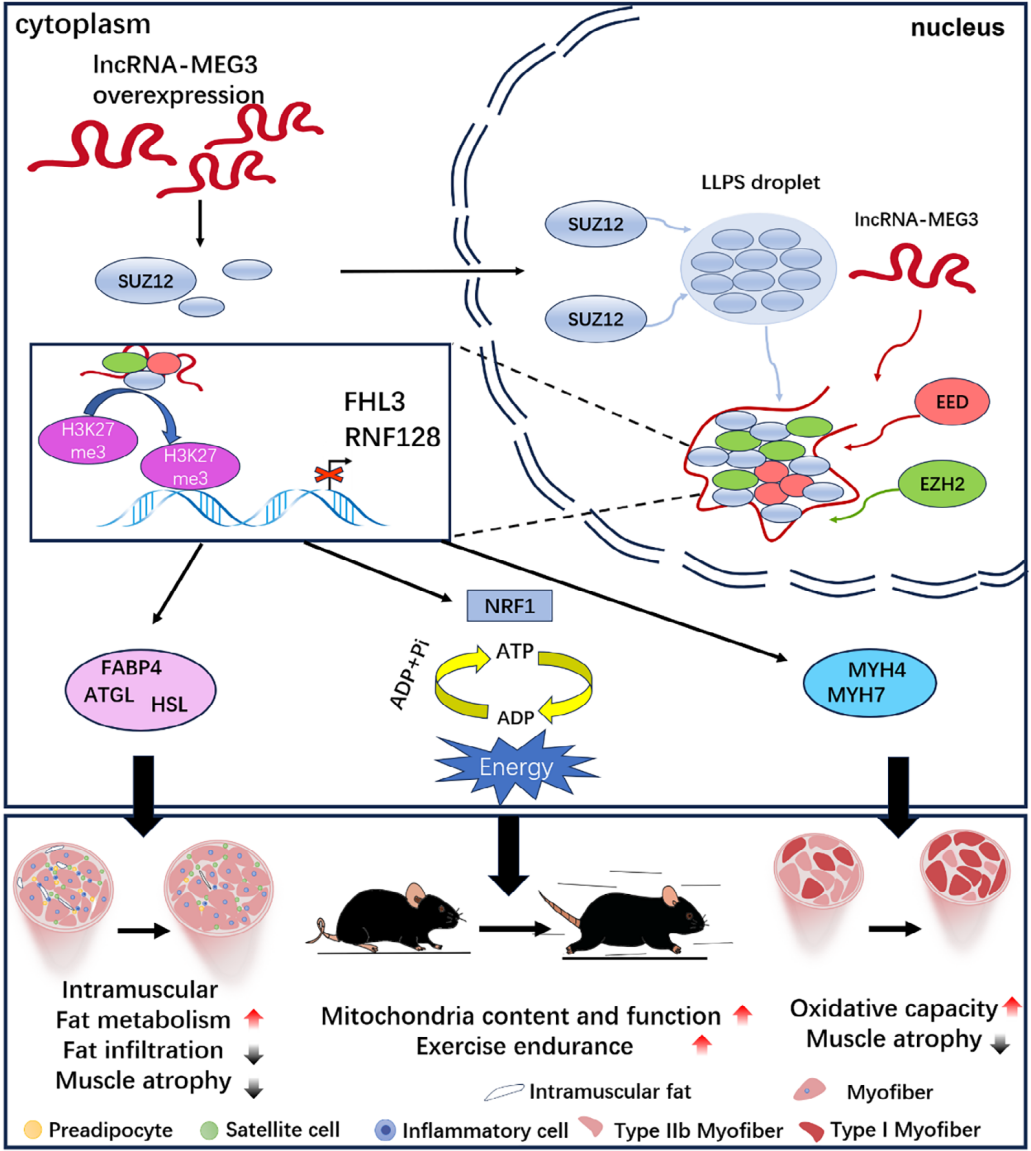
Schematic illustration of the role and mechanism of energy metabolism reprogramming. Triggered by lncRNA‐MEG3 overexpression in skeletal muscle. LncRNA‐MEG3 is specifically induced in skeletal muscle in response to normal muscle development, stimulating the translocation and accumulation of SUZ12 protein in the nucleus, where it condenses into liquid droplets through the mechanism of LLPS. lncRNA‐MEG3 acts as a scaffold, recruiting SUZ12 and increasing its local concentration, promoting its interaction with EED and EZH2. This complex is sequestered together in the droplets, forming membrane‐less subcellular compartments, which catalyze the enhancement of H3K27me3, thereby inhibiting the transcriptional activity of downstream target genes. Through this mechanism, lncRNA‐MEG3 promotes Suz12 phase separation, suppressing the expression of Fhl3 and Rnf128, and further affecting the expression of genes involved in lipid metabolism, muscle atrophy, muscle fiber type conversion, and mitochondrial biogenesis, such as Nrf1, Myh7, Myh4, Adipoq, and Fabp4. These drive enhanced skeletal muscle mass and endurance, inhibit fat infiltration, and prevent muscle atrophy.

The proper development and maintenance of skeletal muscle are pivotal for overall human health, as disruptions in muscle homeostasis are linked to various diseases, including cachexia, sarcopenia, and metabolic disorders. Many factors involved in skeletal muscle regulation also play crucial roles in the pathogenesis and treatment of these muscle‐related diseases.^[^ ^32^, ^33^ ^]^ The long non‐coding RNA (lncRNA) MEG3, first implicated in developmental defects following chromosome duplications in both mice and humans,^[^ ^34^, ^35^ ^]^ has since emerged as a critical modulator of tumors and cancers.^[^ ^36^ ^]^ Over the past decade, studies have demonstrated that the loss of lncRNA‐MEG3 disrupts myocyte differentiation across diverse animal models, including mice, cattle, and pigs.^[^ ^18^, ^19^, ^35^ ^]^ Our study extends these findings, demonstrating that lncRNA‐MEG3 not only regulates muscle mass and strength but also presents itself as a potential therapeutic target for combating muscle atrophy. To explore the underlying mechanisms of lncRNA‐MEG3, we employed a variety of in vivo models, including MDX mice and CTX‐ and GLY‐induced muscle injury models, alongside transgenic technologies and gene delivery systems to manipulate the expression of lncRNA‐MEG3 in skeletal muscle. Through integrated transcriptomic and CUT&Tag analyses, we identified a key role for lncRNA‐MEG3 in regulating mitochondrial function and lipid metabolism. Specifically, we found that lncRNA‐MEG3 promotes the LLPS of SUZ12, a core component of the Polycomb Repressive Complex 2 (PRC2). This interaction stabilizes the PRC2 complex, allowing it to catalyze H3K27me3 histone modifications on muscle‐related genes such as Fhl3 and Rnf128. In turn, these epigenetic changes govern the expression of downstream targets critical for muscle function, including genes (Atrogin‐1, Murf‐1, Adipoq, Fabp4, Perilipin‐1, Nrf1, Sdhb, Myhc, Myh4, and Myh7) involved in mitochondrial biogenesis, lipid metabolism, fiber‐type conversion and muscle atrophy (**Figure** **9**). Skeletal muscle atrophy is a common pathological feature in a wide range of diseases, including aging, cancer‐associated cachexia, and diabetes.^[^ ^37^, ^38^, ^39^ ^]^ In our study, we identified cytokines (IL1β, TNFα, and IL6) that suppress lncRNA‐MEG3 expression in cancer cell culture media, suggesting that dysregulated lncRNA‐MEG3 expression could contribute to muscle loss in these pathological conditions. The molecular pathways through which lncRNA‐MEG3 maintains skeletal muscle growth and quality provide important insights into its potential as a target for therapeutic intervention.

One of the most intriguing findings of our study is the role of lncRNA‐MEG3 in regulating adipogenesis within skeletal muscle. Using single‐cell RNA sequencing, we previously observed that lncRNA‐MEG3 knockdown leads to an increase in fibro‐adipogenic progenitor (FAP) cells, underscoring its involvement in adipogenesis and pathological fat deposition.^[^ ^19^ ^]^ Muscle injury and disease often lead to the differentiation of mesenchymal stem cells into adipogenic precursors, contributing to pathological intramuscular fat accumulation.^[^ ^40^ ^]^ In this study, we demonstrate that lncRNA‐MEG3 suppresses this adipogenic shift, preserving skeletal muscle mass and function. Overexpression of lncRNA‐MEG3 in both primary muscle‐derived adipocytes and injury models partially restored muscle fiber morphology, reduced fat infiltration, and improved myotube diameter and endurance. This suggests that lncRNA‐MEG3 plays a key role in mitigating fat deposition, a hallmark of muscle atrophy in various pathological conditions. Muscle atrophy is frequently associated with a shift in muscle fiber composition. In diseases such as cancer, sepsis, and aging, fast glycolytic fibers (type II) are particularly susceptible to atrophy, whereas slow oxidative fibers (type I) are more resistant.^[^ ^41^, ^42^ ^]^ Our study revealed that lncRNA‐MEG3 preferentially induces atrophy in glycolytic muscle fibers and promotes a transition towards slow myosin heavy chain (MHC) isoforms. This shift is accompanied by enhanced mitochondrial content and improved fatigue resistance in mice, which aligns with existing evidence that slow fibers contain higher mitochondrial densities and are more endurance‐oriented.^[^ ^43^ ^]^ These findings further underscore the role of lncRNA‐MEG3 in regulating both muscle fiber‐type plasticity and mitochondrial function.

A major insight from our work is the identification of SUZ12 as a key regulatory protein in the lncRNA‐MEG3 signaling axis. Our data show that the full‐length lncRNA‐MEG3 is essential for the LLPS of SUZ12, facilitating its interaction with other PRC2 components, including EZH2 and EED.^[^ ^27^, ^44^ ^]^ This RNA‐dependent interaction plays a critical role in stabilizing the PRC2 complex, ensuring the proper modification of H3K27me3 at target gene promoters.^[^ ^45^ ^]^ By modulating these epigenetic changes, lncRNA‐MEG3 governs the expression of Fhl3 and Rnf128. Fhl3 is a gene that promotes the formation of fast‐twitch muscle fibers, while Rnf128 facilitates fat accumulation.^[^ ^28^, ^29^ ^]^ Herein, we also found that Fhl3 and Rnf128 jointly regulate the expression of mitochondrial biogenesis genes, ATP and cAMP formation, and muscle atrophy. These results establish the lncRNA‐MEG3/SUZ12/Fhl3/Rnf128 axis as a key regulatory pathway in muscle mass maintenance and atrophy.

LLPS is essential for the dynamic assembly of biomolecules and the formation of membrane‐less organelles that regulate molecular interactions and organelle function.^[^ ^46^ ^]^ Proteins with IDRs drive LLPS because of their flexible, chain‐like structures, which allow multivalent, low‐affinity interactions that promote biomolecular condensate formation.^[^ ^46^, ^47^, ^48^ ^]^ Some proteins and transcription factors enriched in IDR and low‐complexity domains, such as PSPC1, TRA, and Rho, influence gene expression and biological processes through LLPS.^[^ ^49^, ^50^, ^51^ ^]^ Our study provides the first evidence of LLPS in regulating muscle mass, lipid deposition, and atrophy. We reveal that SUZ12, through its proline‐rich IDR, drives LLPS, a process that we show is essential for its function in skeletal muscle. The deletion of the IDR region of SUZ12 impairs its ability to undergo LLPS, disrupting the H3K27me3 modifications at the promoters of Fhl3 and Rnf128 and, consequently, reducing their expression. Notably, LLPS in the skeletal muscle has rarely been reported, with factors such as FUS and TDP‐43 being linked to amyotrophic lateral sclerosis (ALS), a neurodegenerative disease affecting muscle function.^[^ ^52^, ^53^ ^]^ Our findings suggest that similar LLPS mechanisms may also be at play in muscle cells, with a directly impact on muscle health and disease. These novel insights into the molecular mechanisms of phase separation in skeletal muscle provide a compelling avenue for future research into LLPS‐related therapeutic strategies.

The role of RNA in LLPS has gained increasing attention, particularly in the context of lncRNAs, which have been shown to modulate the formation and dynamics of phase‐separated compartments.^[^ ^54^ ^]^ For instance, the lncRNA GIRGL drives CAPRIN1‐mediated LLPS, inhibiting the translation of glutamyl‐tRNA synthetase‐1.^[^ ^55^ ^]^ LncRNA MNX1‐AS1 enhances IGF2BP1 phase separation, promoting the MNX1‐AS1/IGF2BP1/c‐Myc signaling axis and facilitating cell cycle progression and proliferation in lung cancer cells.^[^ ^56^ ^]^ SNHG9 regulates the phase separation of the Hippo signaling node kinase LATS1, modulating YAP oncogenic signaling and tumorigenesis.^[^ ^57^ ^]^ In our study, we demonstrate that lncRNA‐MEG3 promotes the LLPS of SUZ12, facilitating the formation of stable RNA‐protein condensates, which are essential for maintaining the stability of the PRC2 complex and its epigenetic regulatory functions. This work not only highlights the importance of RNA‐mediated LLPS in muscle biology but also suggests that targeting lncRNA‐MEG3 or its interactions with SUZ12 may offer novel therapeutic strategies for muscle‐related diseases.

## 4 Conclusion

This study identified lncRNA‐MEG3 as a pivotal regulator of skeletal muscle homeostasis, driving the expansion of slow/oxidative muscle fibers and influencing muscle mass regulation, regeneration, and disease pathogenesis. Our findings establish that lncRNA‐MEG3 is essential for maintaining muscle function, with its overexpression enhancing muscle strength, endurance, and regeneration, while its depletion leads to widespread muscle atrophy and mitochondrial dysfunction. Mechanistically, we revealed that lncRNA‐MEG3 exerts its effects by stabilizing the SUZ12‐containing PRC2 complex, thereby modulating key signaling pathways involved in muscle fiber type specification, mitochondrial biogenesis, and lipid metabolism. Notably, lncRNA‐MEG3 regulates SUZ12 LLPS, influencing the expression of downstream muscle‐specific genes such as Fhl3 and Rnf128. These findings not only shed new light on the molecular mechanisms governing muscle fiber remodeling but also highlight the therapeutic potential of lncRNA‐MEG3 for combating muscle‐wasting conditions, such as Duchenne muscular dystrophy. In conclusion, lncRNA‐MEG3 emerges as a powerful and novel target, offering promising avenues for the development of targeted therapies to address muscle degenerative diseases.

## 5 Experimental Section

### Ethical Declaration

All animals used in the present study were handled in compliance with the guidelines (AGIS2020.04.17) provided by the Biomedical Research Ethics Committee of the Agricultural Genomics Institute at Shenzhen, Chinese Academy of Agricultural Sciences.

### Animal Models and Experimental Procedures

Female C57BL/6J mice aged 6 weeks, 8 weeks, 5 months, 6 months, 8 months, and 18 months were used in this study. Mice were housed at 72° Fahrenheit under a 12‐h light/12‐h dark cycle and maintained on a standard chow diet. Mice had ad libitum access to food and water. Transgenic lncRNA‐MEG3 (TG) mice were obtained from the Beijing Institute of Animal Science and Veterinary Medicine (2 Yuanmingyuan West Road, Beijing, China). Non‐transgenic mice used in the study were the non‐transgenic offspring of transgenic parental mice.

The Adeno‐associated viral (AAV‐9) vectors produced by HanBio Biotechnology were used to package the transcript (ENSG00000235116) of lncRNA‐MEG3 and shRNA for overexpression and knockdown experiments. Neonatal mice were intraperitoneally injected with 1 × 10^12^ viral genome particles to induce systemic interference, followed by analysis after 8 weeks. At 5 weeks of age, the mice were injected with 1 × 10^12^ viral genome particles into the TA muscle for local muscle‐specific interference and overexpression, and analysis was performed 8 weeks later. shRNA sequence was listed in Table S9 (Supporting Information).

MDX model (Duchenne muscular dystrophy (MDX, hE8‐30) mice were procured from CYAGEN Biosciences (Guangzhou, China). For lncRNA‐MEG3 overexpression in MDX, 8‐week‐old DMD male mice were intraperitoneally injected with AAV9 vectors carrying either a scrambled control or a lncRNA‐MEG3 overexpression vector (viral titer: 1 × 10¹¹ particles/mouse; HanBio Biotechnology, Beijing, China)

### Regeneration Model

For the muscle regeneration experiment, 6‐week‐old mice with AAV9‐mediated skeletal muscle‐specific knockdown of lncRNA‐MEG3 and lncRNA‐MEG3 TG mice were injected with 100 µL of cardiotoxin (CTX; 10 mm solution, Sigma–Aldrich) into the TA muscle to induce muscle injury. TA muscles were collected at 3.5, 5.5, 10, and 25 days post‐CTX treatment for histological (H&E staining), IF, and statistical analyses. All mice were euthanized by cervical dislocation, with measures taken to minimize animal discomfort throughout the experimental procedures.

### GLY Injection Model

Fifty microliters of 50% v/v GLY (Sigma, USA) were injected into the left TA muscle of six 8‐week‐old lncRNA‐MEG3 TG mice and an equal number of age‐matched WT mice. Mice were allowed to recover for 14 days post‐injection (dpi) before being euthanized, and muscles were collected for analysis.

### Muscle Strength and Treadmill Performance Testing

Limb Grip Strength: Limb grip strength was measured using the grip‐test meter system (XR501, XinRuan, Shanghai, China). Mice were allowed to grip a metal grid using their front paws while being gently pulled backward until their hind limbs released the grid. Each mouse performed four trials, and the average grip strength was calculated for each individual. The investigator conducting the test was blinded to the treatment groups.

Treadmill Endurance Testing: Treadmill testing was performed using an eight‐lane treadmill system (ZH‐PT/5S, Zhenghua, Anhui, China). Mice were acclimated to treadmill running at a speed of 6 m min^−1^ for 5 consecutive days before undergoing a 4‐week chronic treadmill exercise protocol. On the final day, an exhaustive exercise test was conducted on a treadmill inclined at a fixed 10° slope. The protocol involved progressive running, starting at 10 m min^−1^ for 15 min, followed by sequential increases to 13, 16, 19 , and 22 m min^−1^, each for 3 min. The mice then ran at 25 m min^−1^ until exhaustion. Exhaustion was defined as the inability to move off an electric grid (35 V) located at the treadmill's end for a continuous duration of 5 s. Running time and total distance covered by each mouse were recorded. To minimize diurnal variability, all tests were conducted at the same time of the day. Throughout the experiments, animals were consistently handled and tested by the same investigators, who were blinded to group assignments to ensure unbiased data collection.

### ATP Quantification

High‐resolution respirometry was performed using an Oxygraph‐2k unit (Oroboros Instruments, Innsbruck). The instrument was set to 37 °C with a stirring speed of 750 rpm and a data averaging interval of 2 s (Datlab 8.1, Oroboros Instruments). Skeletal muscle (5 mg) was isolated from mice, cut into small pieces, and homogenized with a glass tissue grinder in 400 µL of mitochondrial respiration solution on ice. Tissue homogenate was used for the analysis of mitochondrial respiratory function. After 5 min equilibration period, 2 µm Magnesium Green (MgG) was first titrated to examine the mitochondrial ATP production.

### Mitochondrial Oxygen Consumption Analysis in Skeletal Muscle Fibers

Mitochondrial oxygen consumption in GAS muscle fibers isolated from lncRNA‐MEG3 knockdown and overexpression mice was measured using an Oxygraph‐2k units (Oroboros Instruments, Innsbruck) at 25 °C. Muscle fibers were treated with either 0.05% dimethylformamide (vehicle) or 300 µm menadione for 15 min while rotating on a MACSMIX rotator (Miltenyi Biotec). After centrifugation (100 × g), cells were washed with Krebs‐Henseleit buffer, incubated for the designated period, and then resuspended in MiR05 medium (2.5 × 10⁵ cells mL^−1^) for oxygen consumption analysis. The assessment included: 1) basal oxygen consumption; 2) proton leak (Leak) after oligomycin (2.5 µm) addition; 3) maximal mitochondrial capacity after FCCP titration (final concentration 0.1 µm); 4) Complex I (CI)‐linked respiration following glutamate, malate, and ADP addition, and CI/CII‐dependent respiration after succinate. To distinguish CI‐ and CII‐mediated respiration, rotenone (0.5 µm) was used. Coupling efficiency and proton leak were analyzed post‐oligomycin treatment. Finally, non‐mitochondrial oxygen consumption was measured after antimycin A (2.5 µm) addition for correction.

### In Vivo Muscle Contraction Force Measurement

The contractile force of the TA muscle was assessed using the Aurora Scientific 1300A 3‐in‐1 whole animal system, in accordance with previously described protocols.^[^ ^58^, ^59^ ^]^ Mice were anesthetized through intraperitoneal injection of 3‐bromo‐2‐fluorobenzyl alcohol (31.2 g kg^−1^ body weight). After anesthesia, the TA muscle was carefully isolated from tendon to tendon. The distal tendon was secured with surgical suture, looped, and attached to the lever arm hook for force measurements. To determine the maximum isometric tetanic force, the TA muscle was electrically stimulated at varying frequencies (pulse width: 2 ms; frequency range: 1–300 Hz; duration: 500 ms; voltage: 100 V; inter‐stimulation interval: 1 min). During the experiment, the muscle was continuously perfused with pre‐warmed (37 °C) phosphate‐buffered saline (PBS) to maintain physiological conditions.

### Sectioning and Staining

Skeletal muscle tissues were immediately immersed in a 30% sucrose solution and embedded in isopentane pre‐cooled with liquid nitrogen. The embedded tissues were sectioned into 10 µm thick slices using cryosections (Leica CM1950, Leica, Germany) for subsequent staining procedures. Hematoxylin and Eosin (H&E) Staining: tissue sections were stained with hematoxylin for 20 min to visualize nuclei, followed by differentiation and bluing. The cytoplasm was then counterstained with eosin for 5 min. To conduct SDH Staining: cryosections (10 µm) were incubated at 37 °C for 30 min in a solution containing 0.2 m sodium phosphate buffer (pH 7.6), 0.6 mm nitro blue tetrazolium, and 50 mm sodium succinate (Sigma–Aldrich, St. Louis, MO, USA). After incubation, sections were rinsed with distilled water and mounted using an aqueous mounting medium. For sections IF: muscle sections were incubated overnight at 4 °C with primary antibodies specific to embryonic Myhc (MF‐20, 1:50 dilution, DSHB), Laminin (ab11575, 1:500 dilution, Abcam), MyHC1 (Myh7) (BA‐D5, 1:100 dilution, DSH), MyHC2b (Myh4) (BF‐F3, 1:50 dilution, DSHB), MyHC2a (Myh2) (SC‐71, 1:100 dilution, DSHB) and Perilipin‐1 (ab317260, 1:1000 dilution, Abcam). After washing, secondary antibodies (Goat anti‐Mouse IgG2b Cross‐Adsorbed Secondary Antibody, Alexa Fluor 488, A‐21141, 1:100 dilution, Goat anti‐Mouse IgG1 Cross‐Adsorbed Secondary Antibody, Alexa Fluor 594, A‐21125, 1:100 dilution, and Goat anti‐Mouse IgM (Heavy chain) Secondary Antibody, Alexa Fluor 647, A‐21238, 1:100 dilution, Thermo Fisher Scientific, Waltham, MA, USA) were incubated for 1 h at 37 °C. Images were acquired using a Nikon A1HD25 microscope (Nikon, Japan). To assess lipid content, muscle sections were stained with ORO for 30 min at room temperature. For fluorescent visualization of lipid droplets, sections were stained with BODIPY dye (BODIPY 493/503, MCE, USA), which emits green fluorescence. Imaging: All stained sections were imaged using a Nikon A1HD25 Confocal Microscope (Nikon, Japan). The setup enabled high‐resolution visualization for detailed morphometric and histological analyses.

### Transmission Electron Microscopy (TEM) and Image Analysis

For TEM analysis, GAS muscle samples were immediately fixed overnight in 2.5% glutaraldehyde after collection. The sample was infiltrated with a mixture of epichlorohydrin and Epon 812 resin (EMS, Hatfield, PA, USA) for observation and imaging under the Hitachi Bio TEM (Tokyo, Japan) electron microscope facility of Wuhan Sevier Biotechnology Co., Ltd. Each slice captured at least five views of mitochondrial populations between muscle fibers and beneath the muscle membrane. ImageJ was used to calculate the number of mitochondria.

### Single‐Cell RNA Sequencing (scRNA‐seq) Analysis

We obtained publicly available single‐cell RNA sequencing (scRNA‐seq) datasets from human skeletal muscle atlas (GSE147457),^[^ ^22^ ^]^ spatiotemporal transcriptomic maps of whole mouse embryos (https://cells.ucsc.edu/mouse‐limb/10x/200120_10x.h5ad),^[^ ^60^ ^]^ human muscle aging cell atlas database (https://db.cngb.org/cdcp/hlma/rnaseq/)^[^ ^24^ ^]^ and DMD model (GSM4732632).^[^ ^23^ ^]^ ScRNA‐seq data were processed and analyzed following standard workflows. Low‐quality cells with mitochondrial gene content >15% or gene counts <200 or >8000 were filtered out, and doublet removal was performed using DoubletFinder to ensure data purity. Gene expression data were normalized using the log‐normalization method implemented in Seurat (v5.1.0), where UMI counts were log‐transformed and scaled to 10 000 to mitigate sequencing depth and batch effects. The top 2000 highly variable genes were identified and standardized using z‐score normalization to ensure comparability across different cell types. Principal component analysis (PCA) was performed to capture major biological variations, retaining the top 15 principal components (PCs) for downstream analysis. Batch effect correction was conducted using Harmony (v1.2.1) based on the PCA results. The corrected embeddings were then subjected to dimensionality reduction using uniform manifold approximation and projection (UMAP) for visualization. Louvain clustering was performed using the FindClusters function in Seurat (resolution = 1) to define distinct cell populations. Cell populations were then annotated based on established marker genes to ensure biological relevance and accuracy.^[^ ^22^, ^23^, ^60^ ^]^ Finally, key marker gene expression patterns were visualized using UMAP and violin plots, providing an intuitive representation of the transcriptional profiles across distinct cell types.

### Primers and Oligonucleotides

Primers were designed using Primer Premier5. All primer sequences are provided in Table S10 (Supporting Information). The Biotin‐lncRNA‐MEG3, siRNAs of lncRNA‐MEG3 and Suz12, and siRNA NC were obtained from GenePharma (Shanghai, China). All siRNA sequences are provided in Table S11 (Supporting Information).

### Plasmid Construction

The full‐length lncRNA‐MEG3, the loop structure mutants of lncRNA‐MEG3 (designated as lncRNA‐MEG3 D1, D2, and D3, collectively referred to as lncRNA‐MEG3 ΔSuz12, which lack Suz12 binding sites), the Suz12 CDS region and Suz12 CDS mutants for analyzing LLPS (ΔIDR‐Suz12, FUSIDR‐Suz12, TIA1IDR‐Suz12, and ΔlncRNA‐MEG3‐Suz12), Fhl3 CDS and Rnf128 CDS were cloned into the pcDNA3.1‐GFP/RFP vector for functional analyses. Expression vector with a deleted CDS region of Suz12 (B1, B2, B3, c1, c2, c3, c4, and c5) were cloned into the pcDNA3.1‐GST‐tag vector for binding analyses. All plasmids were purchased from GeneCreate (Wuhan, China).

### Isolating Primary Myoblasts

Primary myoblasts were isolated from the hindlimb skeletal muscle of male mice. Briefly, the skeletal muscle was minced and digested in type II collagenase (Gibco, Gaithersburg, MD, USA) for 30 min, then filtered through a mesh to remove debris. The cells were subsequently cultured in RPMI‐1640 medium. When the primary myoblasts reached 90% confluence, differentiation was induced with Dulbecco's Modified Eagle Medium (DMEM) containing 5% horse serum (Gibco, Gaithersburg, MD, USA).

### Cell Culture and Transfection

HEK293T cell (iCell‐h237), C2C12 myoblasts (iCell‐m013), porcine myoblasts (iCell‐0017a) and HEPG2 (iCell‐h092), 4T1 (iCell‐m067) and C26 (iCell‐m092) were obtained from iCell Bioscience Inc (Shanghai, China). HEK293T cell, C2C12, and HEPG2,4T1 and C26 were cultured in DMEM (Gibco) supplemented with 10% fetal bovine serum (Gibco) and 1% penicillin/streptomycin (PS, Gibco, Grand Island, NY, USA). Porcine and mice primary myoblasts were cultured in RPMI‐1640 medium supplemented with 10% fetal bovine serum (Gibco, Grand Island, NY, USA) and 1% penicillin/streptomycin (Gibco, Grand Island, NY, USA). All cells were cultured in an incubator at 37 °C with 2% CO_2_. To induce adipogenic differentiation, C2C12 myoblasts were treated with adipogenic DM containing 10% FBS, 1 µM dexamethasone (50‐02‐2, Sigma, USA), 0.5 mm 1‐methyl‐3‐isobutylmethyl‐xanthine (28822‐58‐4, Sigma, USA), and 10 µg ml^−1^ insulin (11061‐68‐0, Sigma, USA). Typically, when the cells reached 50–60% confluence, they were transfected using Lipofectamine 3000 according to the instructions of the manufacturer, and the medium was replaced with a complete medium 6 h after transfection.

When C2C12 myoblasts reached 80–85% confluence in a 6‐well plate, they were treated with cytokines (IL‐1β (PA1000‐4827, IPODIX, China), TNFα (PA1000‐4468, IPODIX, China), and IL‐6 (PA1000‐4864, IPODIX, China)) for 48 h, followed by the analysis of lncRNA‐MEG3 expression. After 3 days of induction in C2C12 myotube, the cells were treated with dexamethasone (Sigma, D‐085, USA) and then switched to adipocyte induction medium containing SVF extracted from the corresponding WT and lncRNA‐MEG3 mice muscles. Analyses were performed after 3 days of induction.

### Adipogenic Differentiation

For adipogenic differentiation, intramuscular preadipocytes were cultured with an induction medium (IM) containing DMEM, 10% fetal bovine serum (FBS), 2.85 mm insulin, 0.3 mm dexamethasone (DEXA), and 0.63 mm 3‐isobutyl‐methylxanthine (IBMX) for 4 days when the cells reached confluence. Subsequently, the cells were switched to a differentiation medium (DM) consisting of DMEM, 10% FBS, and 200 nm insulin for 2 more days until the adipocytes matured. To avoid the impact of cell density on adipogenic differentiation, differentiation was induced when the cells reached 90% confluence.

### ATP Assay

The intracellular ATP concentration was measured using an ATP assay (MAK473, Sigma, USA) kit following the manufacturer's protocol. Briefly, muscle tissues or C2C12 myoblasts were lysed with lysis buffer, then centrifuged at 14 400 g for 5 min at 4 °C. The supernatant was collected for ATP measurement, and protein concentration was determined using a BCA protein assay kit. Next, 20 µl of the supernatant was mixed with 100 µl of ATP assay solution, incubated for 5 min at room temperature, and mixed immediately, and luminescence was measured using a dual‐luciferase reporter detection system (Promega, Madison, WI). ATP concentration was calculated from the standard curve and expressed as nanomoles per microgram of protein.

### cAMP Quantification

The total cellular cAMP content was quantified using the cAMP Direct Enzyme Immunoassay Kit (Enzo Life Sciences). Briefly, the supernatants were removed, and muscle tissues or C2C12 myoblasts were treated with 0.1 m HCl for 10 min at room temperature. The cells were scraped off the plate surface using a cell scraper and centrifuged at ≥600 × g to pelletize cellular debris. The supernatant was then assayed immediately according to the manufacturer's instructions.

### Bioinformatic Analysis

The lncRNA‐MEG3 binding proteins were predicted using catRAPID (http://s.tartaglialab.com/page/catrapid_group). The SUZ12 IDR region was predicted by PLAAC (http://plaac.wi.mit.edu/) and PhaSePred (http://lab.phasep.pro/).

### RNA‐Seq Analysis

The RNA‐seq libraries were sequenced on an Illumina NovaSeq 6000 platform (Novogene, Tianjin, China) using a paired‐end read length of 150 bp. Raw sequencing reads were subjected to quality control using Fastp to remove low‐quality bases and adapter sequences using default settings. Clean reads were then aligned to the reference genome GRCm38 using HISAT2, generating SAM files. The alignment quality score (MAPQ) was set to ≥ 20 to ensure reliable mapping. Subsequently, SAM files were converted to BAM format, sorted, and indexed using SAMtools (v1.21), with the alignment rate exceeding 80% to ensure data quality. Transcript assembly was performed using StringTie (v2.2.3) on the aligned reads, and gene and transcript expression levels were estimated using the gencode.vM25.annotation.gtf annotation file. Expression levels were normalized using TPM (Transcripts Per Million). Data normalization was performed using the DESeq2 package (v1.42.0), where raw counts were normalized by size factors, followed by log2 transformation to stabilize the variance across different expression levels. Principal component analysis (PCA) was used to assess the quality of the data, and the top 2000 most variable genes were selected for PCA to identify potential outliers. Differential expression analysis was conducted using DESeq2 (v1.42.0), with genes having an adjusted *p*‐value < 0.05 and a log2 fold change > 0.58 considered as significantly differentially expressed (DEGs). To gain insights into the biological processes and pathways associated with the DEGs, KEGG (Kyoto Encyclopedia of Genes and Genomes) pathway enrichment analysis was performed using ClusterProfiler (v4.10.0) in R (v4.4.2).

### Extraction of Total RNA and qRT‐PCR

Total RNA was extracted from cells and muscle tissues using TRIzol Reagent (Invitrogen, Shanghai, China). cDNA was synthesized using the HiScript IV All‐in‐One Ultra RT SuperMix for qPCR (R433‐01, Vazyme, Nanjing, China) according to the manufacturer's instructions. qRT‐PCR was performed on a QuantStudio 3 Real‐Time PCR System (Thermo Fisher Scientific) using Taq Pro Universal SYBR qPCR Master Mix (Q712‐02, Vazyme, Nanjing, China) following the manufacturer's protocol. The reaction conditions were 95 °C for 10 s, followed by 40 cycles of 5 s at 95 °C and 30 s at 60 °C. Relative gene expression levels were analyzed using the 2^−△△CT^ method. Mouse GAPDH and U6 were used as endogenous reference genes for normalization. The primers are listed in Table S10 (Supporting Information).

### Western Blot Analysis

Protein samples from muscle tissues and cells were extracted using protein lysis buffer (Thermo Fisher, Waltham, MA, USA), which contained 50 mm Tris (pH 7.5), 150 mm NaCl, 0.5% Nonidet P40, and protease and phosphatase inhibitors. The protein lysates were separated by SDS‐PAGE, transferred to a polyvinylidene fluoride (PVDF) membrane, and immunoblotted with primary antibodies against MyH7 (1:1000, Sigma M8421, Germany), MyH4 (1:1000, Sigma MABT847, Germany), SDHB (1:1000, CST #92649, USA), NRF1 (1:1000, CST #46743, USA), Perilipin‐1 (1:1000, abcam ab317260 USA), FABP4 (1:1000, abcam ab308110, USA), Atrogin‐1 (1:1000, abcam ab168372, USA), Murf‐1 (1:1000, abcam ab183094, USA), Adipoq (1:1000, abcam ab181281, USA), EED (1:1000, CST E4L6E, USA), EZH2 (1:1000, CST D2C9, USA), SUZ12 (1:1000, CST D39F6, USA), LAMP1 (1:1000, Sigma D2D11, Germany), Lamin B1 (1:1000, Sigma SAB2101352, Germany), Fhl3 (1:1000, Affinity DF8826, China), H3K27me3 (1:1000, abcam ab6002, USA), β‐actin (1:1000, CST A5441, USA), Rnf128 (1:1000, abcam ab137088, USA), GST‐Tag (1:1000, Affinity T0007, China), α‐tubulin (1:1000, Affinity AF4651, China) and GAPDH (1:1000, CST 14C10, USA). Membranes were washed for 30 min, incubated with horseradish peroxidase‐conjugated secondary antibodies (GB23303, GB23301, Servicebio, China) for 1 h at room temperature, and washed again for 30 min. The membranes were then incubated in Detection Solution (SuperSignal West Pico PLUS, ThermoFisher, USA) for 1 min at room temperature and exposed to a fluorescent chemiluminescence imaging system (ChemiScope 6100Touch, Clinx, China).

### Immunofluorescence Staining

Treated cells were fixed with 4% paraformaldehyde, then permeabilized with 0.5% Triton for 20 min, blocked with goat serum for 1 h, and then incubated with anti‐MyH7 (1:1000, Sigma M8421, Germany) and anti‐MyH4 (1:1000, Sigma M1570, Germany) for 2 h. Subsequently, cells were incubated with a goat anti‐mice secondary antibody (GB25303, GB21301, Servicebio, China). Finally, DAPI (1:1000, Invitrogen D3571, Shanghai, China) was added, and the cells were observed using a Nikon A1HD25 microscope (Nikon).

### Fluorescence In Situ Hybridization

RNA‐FISH experiments were performed following the manufacturer's protocol for the FISH Kit (G‐fan, Shanghai, China). FISH experiments according were performed to the manufacturer's instructions included with the RNA FISH Kit, which was obtained from GEFAN (Shanghai, China). Briefly, the cells were incubated with the RNA probes in a hybridization buffer for 48 h at 68 °C. The cells were washed three times with SSC buffer, stained with DAPI (1:1000, Invitrogen D3571, Shanghai, China) for 10 min at room temperature, and then examined using a confocal microscope (Nikon A1HD25). The probes were listed in Table S12 (Supporting Information)

### Oil Red O and BODIPY Staining

Oil red O (ORO) staining was performed as described.^[^ ^61^ ^]^ Briefly, fixed cells were incubated in ORO dye solution for 30 min. Staining was observed under a phase‐contrast microscope or in the far‐red spectrum under a confocal microscope (Nikon A1HD25). For BODIPY staining, intracellular lipid droplets were stained with 0.5 nm BODIPY FL (Invitrogen) for 10 min. Following staining, the cells were fixed with 4% paraformaldehyde and examined under a fluorescence microscope for analysis.

### Measurement of Intracellular Free Calcium

Intracellular Ca^2^⁺ levels were quantified by flow cytometry using the calcium‐sensitive fluorescent dye Fluo‐3 AM (S1056, Beyotime, China). Primary myoblasts from treated MDX and WT mice were seeded in 24‐well plates and incubated with 4 µm Fluo‐3 AM at 37 °C for 30 min in the absence of light. Following incubation, the cells were collected, washed twice with Hank's Balanced Salt Solution (HBSS), and analyzed with a FACSCalibur flow cytometer (BD Biosciences, San Jose, CA, USA). The mean fluorescence intensity (MFI) was recorded at 488 nm excitation to assess intracellular calcium levels.

### Detection of Reactive Oxygen Species

Intracellular reactive oxygen species (ROS) were measured using a ROS detection kit (S0035S, Beyotime, China). Treated MDX and WT primary myoblasts were seeded in 12‐well plates and incubated with 10 µm 2′,7′‐dichlorofluorescein diacetate (DCFH‐DA) at 37 °C for 30 min. After incubation, the cells were washed three times with PBS containing 2% FBS to remove excess dye. ROS levels were then assessed by measuring the fluorescence intensity using a FACSCalibur flow cytometer (BD Biosciences, San Jose, CA, USA).

### Enzyme‐Linked Immunosorbent Assay (ELISA)

To measure IL‐6 secretion, primary myoblasts isolated from treated MDX and WT mice were seeded in 24‐well plates and cultured for 24 h. After the incubation period, culture supernatants were collected, and IL‐6 concentrations were determined using an ELISA kit (PI326, Beyotime, China) according to the manufacturer's instructions. The optical density (OD) at 450 nm was measured using a microplate reader, and IL‐6 levels were calculated by comparing the OD values with a standard curve generated using recombinant IL‐6 of known concentrations.

### RNA‐Chromatin Immunoprecipitation (RIP)

RNA immunoprecipitation (RIP) assays were conducted using the RNA Immunoprecipitation Kit (BersinBi, Guangzhou, China) according to the manufacturer's instructions. Briefly, C2C12 myoblasts at 80–90% confluency in 15 cm dish were scraped off, then lysed in complete RIP lysis buffer, after which 100 µL of whole‐cell extract was incubated with RIP buffer containing protein A/G magnetic beads (PB101, Vazyme, China) conjugated with anti‐ SUZ12 (1:1000, CST D39F6, USA). Samples were incubated with Proteinase K (20mg/ml) with shaking to digest the protein, followed by the isolation of immunoprecipitated RNA. Furthermore, purified RNA was subjected to qRT‐PCR analysis to demonstrate the presence of the binding targets using respective primers. The primer sequences are listed in Table S10 (Supporting Information).

### RNA Pull‐Down Assay

Whole‐cell lysates were prepared from treated cells using RNA pull‐down lysis buffer (25 mm Tris‐HCl, pH 7.4; 150 mm KCl; 5 mm EDTA; 5 mm MgCl₂; 1% NP‐40; 0.5 mm DTT) supplemented with protease and RNase inhibitors. The lysates were clarified by centrifugation at 12000 × *g* for 10 min at 4 °C. Biotin‐labeled lncRNA‐MEG3 probes (0.1 µg µL^−1^) were added to the lysates, and the mixture was incubated sequentially at 37 °C for 15 min, room temperature for 15 min, and then at 4 °C for 6 h, and finally to overnight with gentle rotation. Streptavidin‐coated magnetic beads (Thermo Fisher Scientific) were pre‐washed and added to the reaction mix, followed by incubation at 4 °C for 1 h with continuous mixing. The beads were washed five times using RNA pull‐down wash buffer (25 mm Tris‐HCl, pH 7.4; 150 mm KCl; 5 mm EDTA; 5 mm MgCl₂; 0.5% NP‐40), with each wash lasting 10 min. After the final wash, bound proteins were eluted by boiling the beads in SDS sample buffer at 95 °C for 5 min. The eluates were analyzed by western blotting or mass spectrometry to identify proteins interacting with lncRNA‐MEG3. All steps were performed under RNase‐free conditions, and experimental replicates were included to ensure the reliability of the results.

### CUT&Tag Analysis

The CUT&Tag libraries were sequenced on an Illumina NovaSeq 6000 platform (Novogene, Tianjin, China) using a paired‐end read length of 150 bp. Raw sequencing reads were processed with Fastp (default settings) to remove adapter sequences and low‐quality bases, and any reads shorter than 50 bp were excluded. The cleaned reads were then aligned to the GRCm38 reference genome using Bowtie2 (v2.5.4), ensuring a minimum mapping quality score of MAPQ ≥ 30, while retaining only uniquely mapped reads for further analysis. Peak calling was performed using MACS2 (v2.2.9.1), with a q‐value threshold of 0.01 to identify significant peaks. Differential peak analysis was performed using the DiffBind (v3.10.0) R package, incorporating DESeq2 for robust statistical analysis of differential binding events across conditions. For data visualization, aligned reads were converted to bigWig format using deepTools (v3.5.5) for IGV (Integrative Genomics Viewer) visualization. Additionally, heatmaps and metaplots of signal intensity across peaks were generated using deepTools to compare signal enrichment between different conditions. The identified peaks were annotated using ChIPseeker (v1.40.0) with the TxDb. Mmusculus. UCSC.mm10. known Gene annotation package to determine their genomic features and associated genes.

### Chromatin Immunoprecipitation (ChIP) qRT‐PCR Assay

Cells or tissues were fixed with 1% formaldehyde for 10 min at room temperature to crosslink protein‐DNA interactions, and the reaction was quenched by adding 125 mm glycine for 5 min. After washing with cold PBS, the cells or tissues were lysed in a lysis buffer containing protease inhibitors, and the nuclear fraction was collected. Chromatin was sheared to 200–1000 bp fragments using sonication, and the fragment size was verified by agarose gel electrophoresis. Equal amounts of chromatin were incubated overnight with H3K27me3 (ab6002, Abcam, USA) at 4 °C, with normal IgG (ab190475, Abcam, USA) as a negative control and input chromatin as a positive control. Pre‐washed protein A/G magnetic beads (PB101, Vazyme, China) were added, and the mixture was incubated at 4 °C for 1–2 h. The beads were washed sequentially with low‐salt and high‐salt wash buffers to remove nonspecific binding, and chromatin‐protein complexes were eluted using an elution buffer. Crosslinking was reversed by incubating the eluted samples with Proteinase K (20mg/ml) at 65 °C for 4–6 h or overnight. DNA was purified using a DNA extraction kit (G1N70, Sigma, Germany) and subjected to quantitative PCR (qPCR) analysis using primers specific to the target gene promoter region. Enrichment levels were calculated relative to input chromatin and normalized to the IgG control. The primers are listed in Table S13 (Supporting Information).

### Co‐Immunoprecipitation (Co‐IP)

Co‐IP was performed according to the instructions of the Magnetic Co‐Immunoprecipitation (Co‐IP) Kit (Bes3011(S), BersinBio, China). In brief, C2C12 myoblasts were harvested by washing with PBS, and then lysed in lysis buffer containing protease inhibitors, incubating on ice for 10–20 min. The lysate was centrifuged to remove cell debris, and the supernatant was collected and incubated with Protein A/G agarose beads for 1–2 h. Specific antibodies were then added and incubated at 4 °C for 2–4 h or overnight, followed by further incubation with Protein A/G agarose beads for an additional 2 h. The beads were washed 3–5 times, and proteins were eluted using SDS‐PAGE loading buffer, followed by Western blot analysis.

### Protein Expression and Purification

SUZ12 protein expression was induced by the BL21 receptor state. Next, 1 µL SUZ12 protein expression plasmid was transformed into 10 µL BL21 receptive state. The transformed cells were plated on a kanamycin‐resistant culture plate, and monoclonal colonies were selected and inoculated into 5 mL of LB liquid medium containing kanamycin. The culture was incubated overnight at 37 °C in a shaking incubator. Next, 5 mL bacterial solution was inoculated into 400 mL LB liquid medium containing kanamycin and placed in a 37 °C constant temperature shaker at 200 rpm for 3 h. Next, 100 µL of 1 m IPTG was added to induce protein expression, followed by incubation in a shaker at 16 °C for 20 h. The protein of SUZ12 was then purified using the Tianren Protein purification kit HisPur Ni NTA kit (no.SA004K).

### In Vitro Phase Separation Assay

In vitro phase separation assay was performed in a storage buffer with indicated protein concentrations, and PEG8000 was also added to a final concentration of 10% (w/v). Phase separation assay was carried out on glass‐bottomed dishes (NEST), sealed with optically clear adhesive film to prevent evaporation and observed under a Nikon A1HD25 confocal microscope equipped with 60 × oil immersion objectives. The phase separation assay was performed in a physiological LLPS buffer (20 mm Tris‐HCl, pH 7.5, 15 mm NaCl, 130 mm KCl, 5 mm KH2PO4, 1.5 mm MgCl2, and 1 mg mL^−1^ BSA).

### SUZ12 Protein LLPS Detection

In the salt concentration gradient assay, the protein was incubated with buffers containing 37.5, 300, and 500 mm NaCl at 20 °C for 30 min, and droplet formation was observed using Nikon A1HD25 confocal microscopy. The relative droplet area was quantified using ImageJ software. In the pH gradient assay, the protein was diluted in buffers with pH values ranging from 6.0 to 4.5 and incubated at 20 °C for 30 min, followed by Nikon A1HD25 confocal imaging and quantitative analysis. Finally, in the temperature gradient assay, the protein was incubated at different temperatures (4, 20, and 37 °C) in a buffer containing 150 mm NaCl and pH 7.4, with phase separation monitored by Nikon A1HD25 confocal microscopy.

### Fluorescence Redistribution After Photobleaching (FRAP)

FRAP experiments were performed on a Nikon A1HD25 confocal microscope with a 60 × oil immersion objective. For the in vitro experiments, droplets were photobleached with 50% laser power for 0.5 s using 488 nm lasers. Time‐series images were acquired every 3 s after bleaching for 5 min. For the in vivo experiments, FRAP assays were carried out on a Nikon A1HD25 confocal microscope at 37 °C in a live‐cell imaging chamber. Droplets were bleached with a 488‐nm laser pulse (50% intensity, 0.5 s). The recovery from photobleaching was recorded for the indicated time. Analysis of the recovery curves was carried out using ImageJ software.

### Statistical Analysis

For the wet‐lab experiments, statistical analysis was performed using GraphPad Prism 8 (GraphPad Software, San Diego, CA, USA). Data are presented as mean ± standard deviation (SD), and each statistical analysis included at least three biological replicates (n). A two‐tailed Student's *t*‐test was used to assess the statistical significance between the two groups, with tests showing *p* < 0.05 considered statistically significant. Significance levels are indicated as follows: ns (not significant), ^*^ *p* < 0.05, ^**^ *p* < 0.01, ^***^ *p* < 0.001.

## Conflict of Interest

The authors declare no conflict of interest.

## Author Contributions

Y.Y., C.Y., H.H., and S.W. contributed equally to this work. Z.L.T and Y.L.Y. conceived of and oversaw the project. Y.L.Y., S.LW., Y.S.Z., D.Y.F., X.H., and Y.W.L performed the experiments, and H.B.H., C.Y., and Y.L. analyzed the data. X.H. generated the mice. Y.L.Y. and C.Y. wrote the manuscript. J.Y.L., Y.C., and Y.Y.Z. conducted a muscle plasmid electroporation experiment. Z.L.T. and Y.L.Y. provided resources and funding for the investigation.

## Supporting information



Supporting Information

Supporting Information

## Data Availability

The raw sequence data reported in this paper have been deposited into CNGB Sequence Archive (CNSA) (https://db.cngb.org/cnsa/) of China National GeneBank DataBase (CNGBdb) with accession numbers CNP0006569.

## References

[1] S. Mathes , A. Fahrner , U. Ghoshdastider , H. A. Rüdiger , M. Leunig , C. Wolfrum , J. Krützfeldt , Proc. Natl. Acad. Sci. USA 2021, 118, 2021013118. 10.1073/pnas.2021013118 PMC8449320 34493647

[2] N. K. Biltz , K. H. Collins , K. C. Shen , K. Schwartz , C. A. Harris , G. A. Meyer , J. Physiol. 2020, 598, 2669. 32358797 10.1113/JP279595 PMC8767374

[3] M. E. Rosa‐Caldwell , S. Lim , W. A. Haynie , J. L. Brown , J. W. Deaver , F. M. Da Silva , L. T. Jansen , D. E. Lee , M. P. Wiggs , T. A. Washington , N. P. Greene , J. Cachexia Sarcopenia Muscle 2021, 12, 717. 33675163 10.1002/jcsm.12693 PMC8200438

[4] X. Chen , Y. Ji , R. Liu , X. Zhu , K. Wang , X. Yang , B. Liu , Z. Gao , Y. Huang , Y. Shen , H. Liu , H. Sun , J. Transl. Med. 2023, 21, 503. 37495991 10.1186/s12967-023-04369-z PMC10373380

[5] A. Fatica , I. Bozzoni , Nat. Rev. Genet. 2014, 15, 7. 24296535 10.1038/nrg3606

[6] R. Choudhari , M. J. Sedano , A. L. Harrison , R. Subramani , K. Y. Lin , E. I. Ramos , R. Lakshmanaswamy , S. S. Gadad , Adv. Clin. Chem. 2020, 95, 105. 32122521 10.1016/bs.acc.2019.08.003

[7] M. Cesana , D. Cacchiarelli , I. Legnini , T. Santini , O. Sthandier , M. Chinappi , A. Tramontano , I. Bozzoni , Cell 2011, 147, 358. 22000014 10.1016/j.cell.2011.09.028 PMC3234495

[8] M. Zhu , J. Liu , J. Xiao , L. Yang , M. Cai , H. Shen , X. Chen , Y. Ma , S. Hu , Z. Wang , A. Hong , Y. Li , Y. Sun , X. Wang , Nat. Commun. 2017, 8, 14718. 28281528 10.1038/ncomms14718 PMC5353601

[9] T. Chujo , T. Yamazaki , T. Hirose , Biochim. Biophys. Acta. 2016, 1859, 139. 26021608 10.1016/j.bbagrm.2015.05.007

[10] J. M. Engreitz , N. Ollikainen , M. Guttman , Nat. Rev. Mol. Cell Biol. 2016, 17, 756. 27780979 10.1038/nrm.2016.126

[11] A. Wutz , T. P. Rasmussen , R. Jaenisch , Nat. Genet. 2002, 30, 167. 11780141 10.1038/ng820

[12] S. F. Banani , H. O. Lee , A. A. Hyman , M. K. Rosen , Nat. Rev. Mol. Cell Biol. 2017, 18, 285. 28225081 10.1038/nrm.2017.7 PMC7434221

[13] A. Al‐Rugeebah , M. Alanazi , N. R. Parine , Pathol. Oncol. Res. 2019, 25, 859. 30793226 10.1007/s12253-019-00614-3

[14] R. Yang , Y. Liu , Y. Cheng , C. Wang , J. Song , G. Lu , T. Feng , S. Wang , X. Sun , J. Meng , L. Hao , Front. Genet. 2021, 12, 607910. 33692824 10.3389/fgene.2021.607910 PMC7937967

[15] T. L. Dill , A. Carroll , A. Pinheiro , J. Gao , F. J. Naya , Development 2021, 148, dev194027. 33298462 10.1242/dev.194027

[16] Q. Liu , M. Li , S. Xie , C. Tian , J. Li , Y. Wang , X. Li , C. Li , Epigenetics 2023, 18, 2237789. 37506369 10.1080/15592294.2023.2237789 PMC10392761

[17] C. Charlier , K. Segers , D. Wagenaar , L. Karim , S. Berghmans , O. Jaillon , T. Shay , J. Weissenbach , N. Cockett , G. Gyapay , M. Georges , Genome Res. 2001, 11, 850. 11337479 10.1101/gr.172701 PMC311092

[18] X. Yu , Z. Wang , H. Sun , Y. Yang , K. Li , Z. Tang , Anim. Genet. 2018, 49, 571. 30294799 10.1111/age.12712

[19] Y. Yao , Z. Wang , Y. Chen , L. Liu , L. Wang , G. Yi , Y. Yang , D. Wang , K. Li , Z. Tang , Genes Dis. 2023, 10, 359. 37223503 10.1016/j.gendis.2022.04.012 PMC10201586

[20] X. Cheng , M. S. Shihabudeen Haider Ali , M. Moran , M. P. Viana , S. L. Schlichte , M. C. Zimmerman , O. Khalimonchuk , M. W. Feinberg , X. Sun , Redox Biol. 2021, 40, 101863. 33508742 10.1016/j.redox.2021.101863 PMC7844131

[21] V. Kameswaran , M. L. Golson , M. Ramos‐Rodríguez , K. Ou , Y. J. Wang , J. Zhang , L. Pasquali , K. H. Kaestner , Diabetes 2018, 67, 1807. 30084829 10.2337/db17-0682 PMC6110314

[22] H. Xi , J. Langerman , S. Sabri , P. Chien , C. S. Young , S. Younesi , M. Hicks , K. Gonzalez , W. Fujiwara , J. Marzi , S. Liebscher , M. Spencer , B. Van Handel , D. Evseenko , K. Schenke‐Layland , K. Plath , A. D. Pyle , Cell Stem Cell 2020, 27, 158. 32396864 10.1016/j.stem.2020.04.017 PMC7367475

[23] F. Chemello , Z. Wang , H. Li , J. R. McAnally , N. Liu , R. Bassel‐Duby , E. N. Olson , Proc. Natl. Acad. Sci. USA 2020, 117, 29691. 33148801 10.1073/pnas.2018391117 PMC7703557

[24] Y. Lai , I. Ramírez‐Pardo , J. Isern , J. An , E. Perdiguero , A. L. Serrano , J. Li , E. García‐Domínguez , J. Segalés , P. Guo , V. Lukesova , E. Andrés , J. Zuo , Y. Yuan , C. Liu , J. Viña , J. Doménech‐Fernández , M. C. Gómez‐Cabrera , Y. Song , L. Liu , X. Xu , P. Muñoz‐Cánoves , M. A. Esteban , Nature 2024, 629, 154. 38649488 10.1038/s41586-024-07348-6 PMC11062927

[25] K. Z. Zhang , J. W. Li , J. S. Xu , Z. K. Shen , Y. S. Lin , C. Zhao , X. Lu , Y. F. Rui , W. Gao , J. Cachexia Sarcopenia Muscle 2024, 15, 1601. 39031684 10.1002/jcsm.13518 PMC11294031

[26] C. Mao , G. Lei , A. Horbath , M. Wang , Z. Lu , Y. Yan , X. Liu , L. Kondiparthi , X. Chen , J. Cheng , Q. Li , Z. Xu , L. Zhuang , B. Fang , J. R. Marszalek , M. V. Poyurovsky , K. Olszewski , B. Gan , Mol. Cell 2024, 84, 1964. 38759628 10.1016/j.molcel.2024.04.009 PMC11104512

[27] L. Gong , X. Liu , L. Jiao , X. Yang , A. Lemoff , X. Liu , Nat. Commun. 2022, 13, 6781. 36351927 10.1038/s41467-022-34431-1 PMC9645763

[28] W. Bai , Y. Zhang , J. Ma , M. Du , H. Xu , J. Wang , L. Zhang , W. Li , Y. Hou , X. Liu , X. Zhang , Y. Peng , J. Li , X. Zhan , W. Jiang , S. Liu , X. Liu , Q. Li , Y. Miao , M. Sui , Y. Yang , S. Zhang , Z. Xu , B. Zuo , Cell. Mol. Life Sci. 2023, 80, 27. 36602641 10.1007/s00018-022-04680-w PMC11073127

[29] N. Darci‐Maher , M. Alvarez , U. T. Arasu , I. Selvarajan , S. H. T. Lee , D. Z. Pan , Z. Miao , S. S. Das , D. Kaminska , T. Örd , J. N. Benhammou , M. Wabitsch , J. R. Pisegna , V. Männistö , K. H. Pietiläinen , M. Laakso , J. S. Sinsheimer , M. U. Kaikkonen , J. Pihlajamäki , P. Pajukanta , EBioMedicine 2023, 92, 104620. 37224770 10.1016/j.ebiom.2023.104620 PMC10277924

[30] J. H. Ahn , E. S. Davis , T. A. Daugird , S. Zhao , I. Y. Quiroga , H. Uryu , J. Li , A. J. Storey , Y. H. Tsai , D. P. Keeley , S. G. Mackintosh , R. D. Edmondson , S. D. Byrum , L. Cai , A. J. Tackett , D. Zheng , W. R. Legant , D. H. Phanstiel , G. G. Wang , Nature 2021, 595, 591. 34163069 10.1038/s41586-021-03662-5 PMC8647409

[31] M. Du , Z. J. Chen , Science 2018, 361, 704. 29976794 10.1126/science.aat1022 PMC9417938

[32] K. Ramachandran , C. R. Futtner , M. A. Sommars , M. Quattrocelli , Y. Omura , E. Fruzyna , J. C. Wang , N. J. Waldeck , M. D. Senagolage , C. G. Telles , A. R. Demonbreun , E. Prendergast , N. Lai , D. Arango , I. R. Bederman , E. M. McNally , G. D. Barish , Nat. Metab. 2024, 6, 304. 38337096 10.1038/s42255-024-00983-3 PMC10949880

[33] J. Qiu , Y. Guo , X. Guo , Z. Liu , Z. Li , J. Zhang , Y. Cao , J. Li , S. Yu , S. Xu , J. Chen , D. Wang , J. Yu , M. Guo , W. Zhou , S. Wang , Y. Wang , X. Ma , C. Xie , L. Xu , Adv. Sci. 2024, 12, 2411015.

[34] N. Miyoshi , H. Wagatsuma , S. Wakana , T. Shiroishi , M. Nomura , K. Aisaka , T. Kohda , M. A. Surani , T. Kaneko‐Ishino , F. Ishino , Genes Cells 2000, 5, 211. 10759892 10.1046/j.1365-2443.2000.00320.x

[35] S. Kobayashi , H. Wagatsuma , R. Ono , H. Ichikawa , M. Yamazaki , H. Tashiro , K. Aisaka , N. Miyoshi , T. Kohda , A. Ogura , M. Ohki , T. Kaneko‐Ishino , F. Ishino , Genes Cells 2000, 5, 1029. 11168589 10.1046/j.1365-2443.2000.00390.x

[36] J. Xu , X. Wang , C. Zhu , K. Wang , Front. Cell Dev. Biol. 2022, 10, 997633. 36544907 10.3389/fcell.2022.997633 PMC9760833

[37] M. Naruse , S. Trappe , T. A. Trappe , J. Appl. Physiol. 2023, 134, 900. 36825643 10.1152/japplphysiol.00768.2022 PMC10069966

[38] S. F. Schmidt , M. Rohm , S. Herzig , M. Berriel Diaz , Trends Cancer 2018, 4, 849. 30470306 10.1016/j.trecan.2018.10.001

[39] J. Song , J. Liu , C. Cui , H. Hu , N. Zang , M. Yang , J. Yang , Y. Zou , J. Li , L. Wang , Q. He , X. Guo , R. Zhao , F. Yan , F. Liu , X. Hou , Z. Sun , L. Chen , J. Cachexia Sarcopenia Muscle 2023, 14, 915. 36708027 10.1002/jcsm.13177 PMC10067482

[40] A. Reggio , M. Rosina , A. Palma , A. Cerquone Perpetuini , L. L. Petrilli , C. Gargioli , C. Fuoco , E. Micarelli , G. Giuliani , M. Cerretani , A. Bresciani , F. Sacco , L. Castagnoli , G. Cesareni , Cell Death Differ. 2020, 27, 2921. 32382110 10.1038/s41418-020-0551-y PMC7492278

[41] Y. Wang , J. E. Pessin , Curr. Opin. Clin. Nutr. Metab. Care 2013, 16, 243. 23493017 10.1097/MCO.0b013e328360272d PMC4327989

[42] S. Ciciliot , A. C. Rossi , K. A. Dyar , B. Blaauw , S. Schiaffino , Int. J. Biochem. Cell Biol. 2013, 45, 2191. 23702032 10.1016/j.biocel.2013.05.016

[43] S. Schiaffino , C. Reggiani , Physiol. Rev. 2011, 91, 1447. 22013216 10.1152/physrev.00031.2010

[44] S. Cyrus , D. Burkardt , D. D. Weaver , W. T. Gibson , Med. Genet. 2019, 181, 519. 10.1002/ajmg.c.31754 31724824

[45] Y. Long , T. Hwang , A. R. Gooding , K. J. Goodrich , J. L. Rinn , T. R. Cech , Nat. Genet. 2020, 52, 931. 32632336 10.1038/s41588-020-0662-x PMC10353856

[46] Y. Shin , C. P. Brangwynne , Science 2017, 357, aaf4382. 10.1126/science.aaf4382 28935776

[47] S. Boeynaems , S. Alberti , N. L. Fawzi , T. Mittag , M. Polymenidou , F. Rousseau , J. Schymkowitz , J. Shorter , B. Wolozin , L. Van Den Bosch , P. Tompa , M. Fuxreiter , Trends Cell Biol. 2018, 28, 420. 29602697 10.1016/j.tcb.2018.02.004 PMC6034118

[48] S. Basu , S. D. Mackowiak , H. Niskanen , D. Knezevic , V. Asimi , S. Grosswendt , H. Geertsema , S. Ali , I. Jerković , H. Ewers , S. Mundlos , A. Meissner , D. M. Ibrahim , D. Hnisz , Cell 2020, 181, 1062. 32386547 10.1016/j.cell.2020.04.018 PMC7261253

[49] E. Krypotou , G. E. Townsend , X. Gao , S. Tachiyama , J. Liu , N. D. Pokorzynski , A. L. Goodman , E. A. Groisman , Science 2023, 379, 1149. 36927025 10.1126/science.abn7229 PMC10148683

[50] W. Shao , X. Bi , Y. Pan , B. Gao , J. Wu , Y. Yin , Z. Liu , M. Peng , W. Zhang , X. Jiang , W. Ren , Y. Xu , Z. Wu , K. Wang , G. Zhan , J. Y. Lu , X. Han , T. Li , J. Wang , G. Li , H. Deng , B. Li , X. Shen , Nat. Chem. Biol. 2022, 18, 70. 34916619 10.1038/s41589-021-00904-5

[51] S. Zhu , J. Gu , J. Yao , Y. Li , Z. Zhang , W. Xia , Z. Wang , X. Gui , L. Li , D. Li , H. Zhang , C. Liu , Dev. Cell 2022, 57, 583. 35231447 10.1016/j.devcel.2022.02.005

[52] A. Patel , H. O. Lee , L. Jawerth , S. Maharana , M. Jahnel , M. Y. Hein , S. Stoynov , J. Mahamid , S. Saha , T. M. Franzmann , A. Pozniakovski , I. Poser , N. Maghelli , L. A. Royer , M. Weigert , E. W. Myers , S. Grill , D. Drechsel , A. A. Hyman , S. Alberti , Cell 2015, 162, 1066. 26317470 10.1016/j.cell.2015.07.047

[53] H. Sun , B. Yang , Q. Li , X. Zhu , E. Song , C. Liu , Y. Song , G. Jiang , Nat. Nanotechnol. 2024, 19, 1354. 38849544 10.1038/s41565-024-01683-5

[54] K. Somasundaram , B. Gupta , N. Jain , S. Jana , Front. Genet. 2022, 13, 930792. 36035193 10.3389/fgene.2022.930792 PMC9399341

[55] R. Wang , L. Cao , R. F. Thorne , X. D. Zhang , J. Li , F. Shao , L. Zhang , M. Wu , Sci. Adv. 2021, 7, abe5708. 10.1126/sciadv.abe5708 PMC7990344 33762340

[56] Q. Zhu , C. Zhang , T. Qu , X. Lu , X. He , W. Li , D. Yin , L. Han , R. Guo , E. Zhang , Cancer Res. 2022, 82, 4340. 36214649 10.1158/0008-5472.CAN-22-1289

[57] R. H. Li , T. Tian , Q. W. Ge , X. Y. He , C. Y. Shi , J. H. Li , Z. Zhang , F. Z. Liu , L. J. Sang , Z. Z. Yang , Y. Z. Liu , Y. Xiong , Q. Yan , X. Li , H. Q. Ju , J. Liu , L. J. Wang , J. Z. Shao , W. Wang , T. Zhou , A. Lin , Cell Res. 2021, 31, 1088. 34267352 10.1038/s41422-021-00530-9 PMC8486796

[58] B. D. Cosgrove , P. M. Gilbert , E. Porpiglia , F. Mourkioti , S. P. Lee , S. Y. Corbel , M. E. Llewellyn , S. L. Delp , H. M. Blau , Nat. Med. 2014, 20, 255. 24531378 10.1038/nm.3464 PMC3949152

[59] J. Xu , X. Li , W. Chen , Z. Zhang , Y. Zhou , Y. Gou , C. A. Lv , L. Jin , X. Qiu , S. Ma , Q. Q. Wu , T. Liu , L. Mi , Z. Yang , T. Yu , X. Pan , Y. Feng , P. Shan , Z. X. Meng , J. Exp. Med. 2023, 220, 20221123. 10.1084/jem.20221123 PMC10250555 37284884

[60] P. He , B. A. Williams , D. Trout , G. K. Marinov , H. Amrhein , L. Berghella , S. T. Goh , I. Plajzer‐Frick , V. Afzal , L. A. Pennacchio , D. E. Dickel , A. Visel , B. Ren , R. C. Hardison , Y. Zhang , B. J. Wold , Nature 2020, 583, 760. 32728245 10.1038/s41586-020-2536-x PMC7410830

[61] X. Chen , H. Yong , M. Chen , C. Deng , P. Wang , S. Chu , M. Li , P. Hou , J. Zheng , Z. Li , J. Bai , Cancer Res. 2023, 42, 34. 10.1186/s13046-022-02583-z PMC9875457 36694250

