# Supplementary material for: LncRNA‐MEG3 Regulates Muscle Mass and Metabolic Homeostasis by Facilitating SUZ12 Liquid–Liquid Phase Separation
Source: Adv Sci (Weinh). 2025 Apr 26;12(23):2417715. doi: 10.1002/advs.202417715 (PMC12199413; doi:10.1002/advs.202417715)
Supplement: Supplementary file 1 — Supporting Information [file ADVS-12-2417715-s002.docx]

**Supplementary Figure legends**


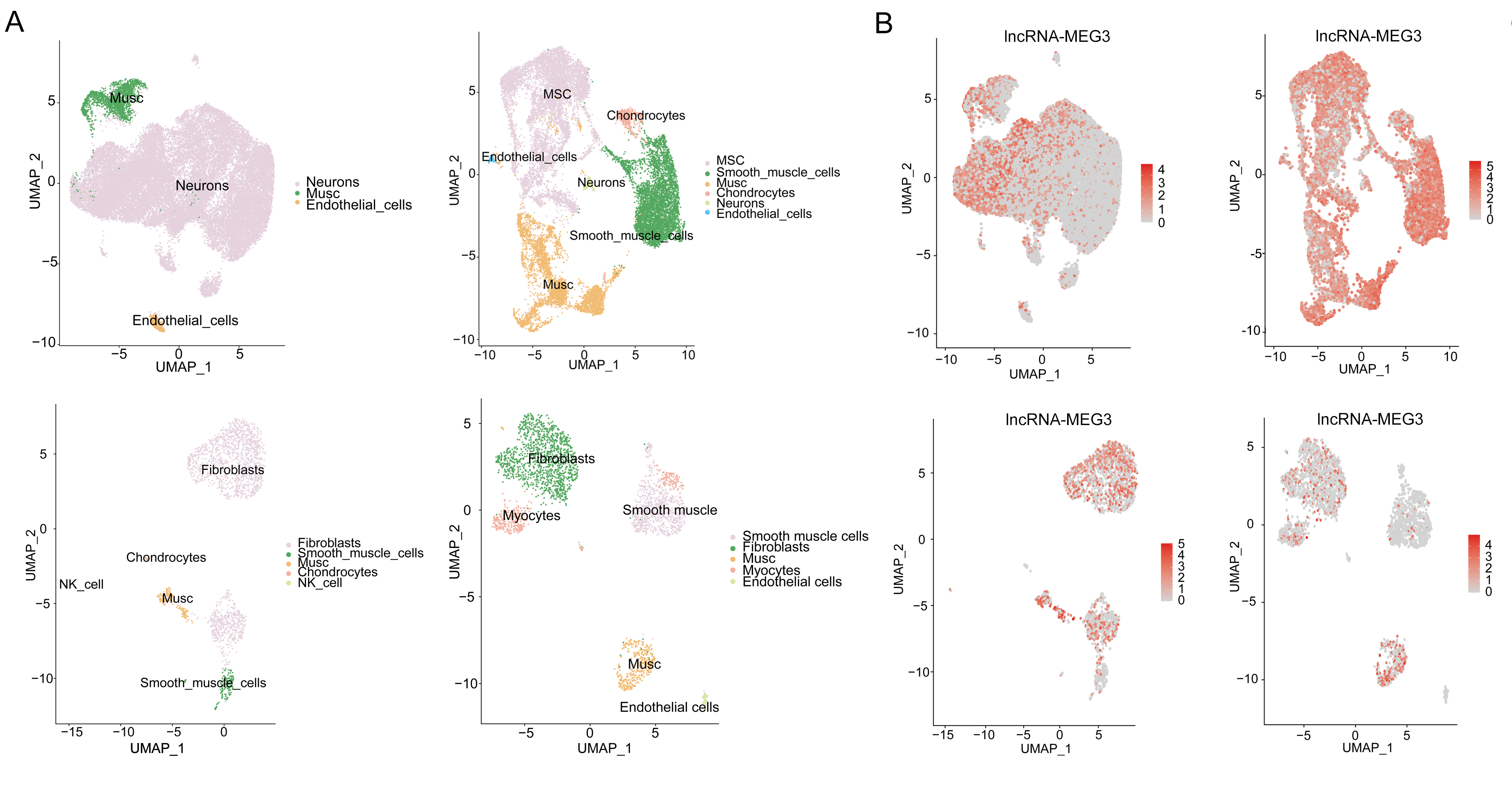


**Figure S1.** Single-cell transcriptomic analysis reveals cell-type-specific expression of lncRNA-MEG3 in skeletal muscle. (A) UMAP visualization of single-cell RNA-sequencing (scRNA-seq) data showing the distribution of different cell types and annotated cell cluster. (B) Expression distribution of lncRNA-MEG3 across cell types at the single-cell level, highlighting its cell-type-specific expression.


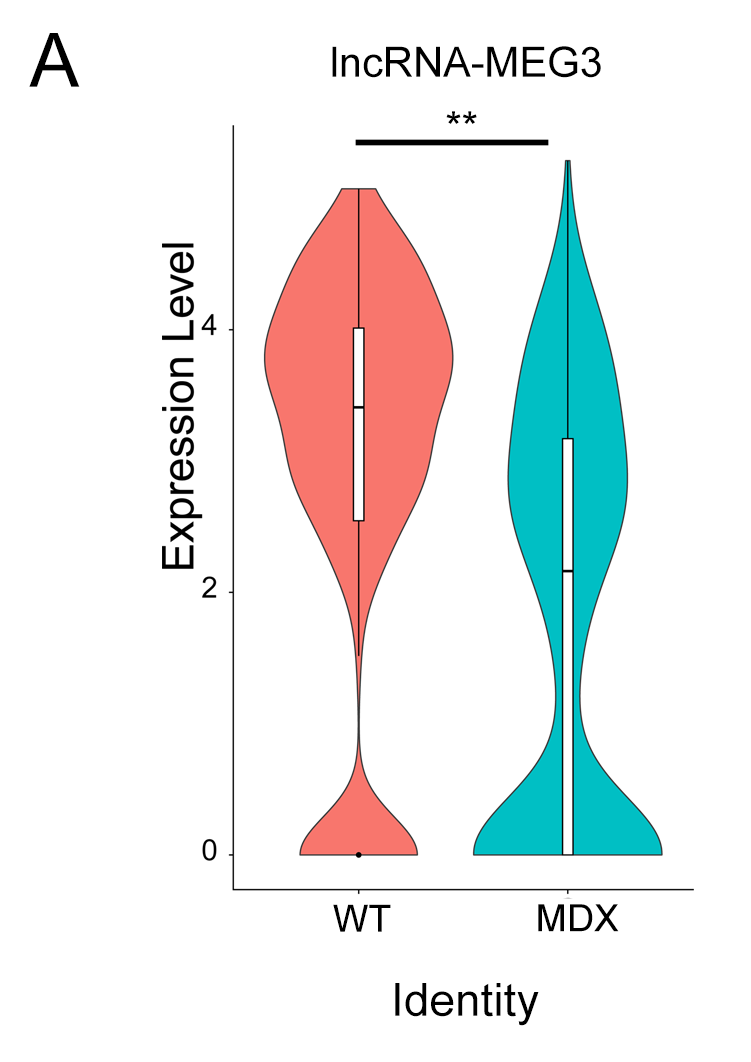


**Figure S2.** Single-cell transcriptomic analysis reveals lncRNA-MEG3 expression in MDX mice skeletal muscle. (A) Violin plots showing the expression levels of lncRNA-MEG3 in WT and MDX mice MuSCs cluster (*n* = 3). Differential expression analysis was performed using DESeq2 (v1.30.0), with a filtering threshold of p-value < 0.05 and |log2(fold change)| > 1. ***P* < 0.01.

**
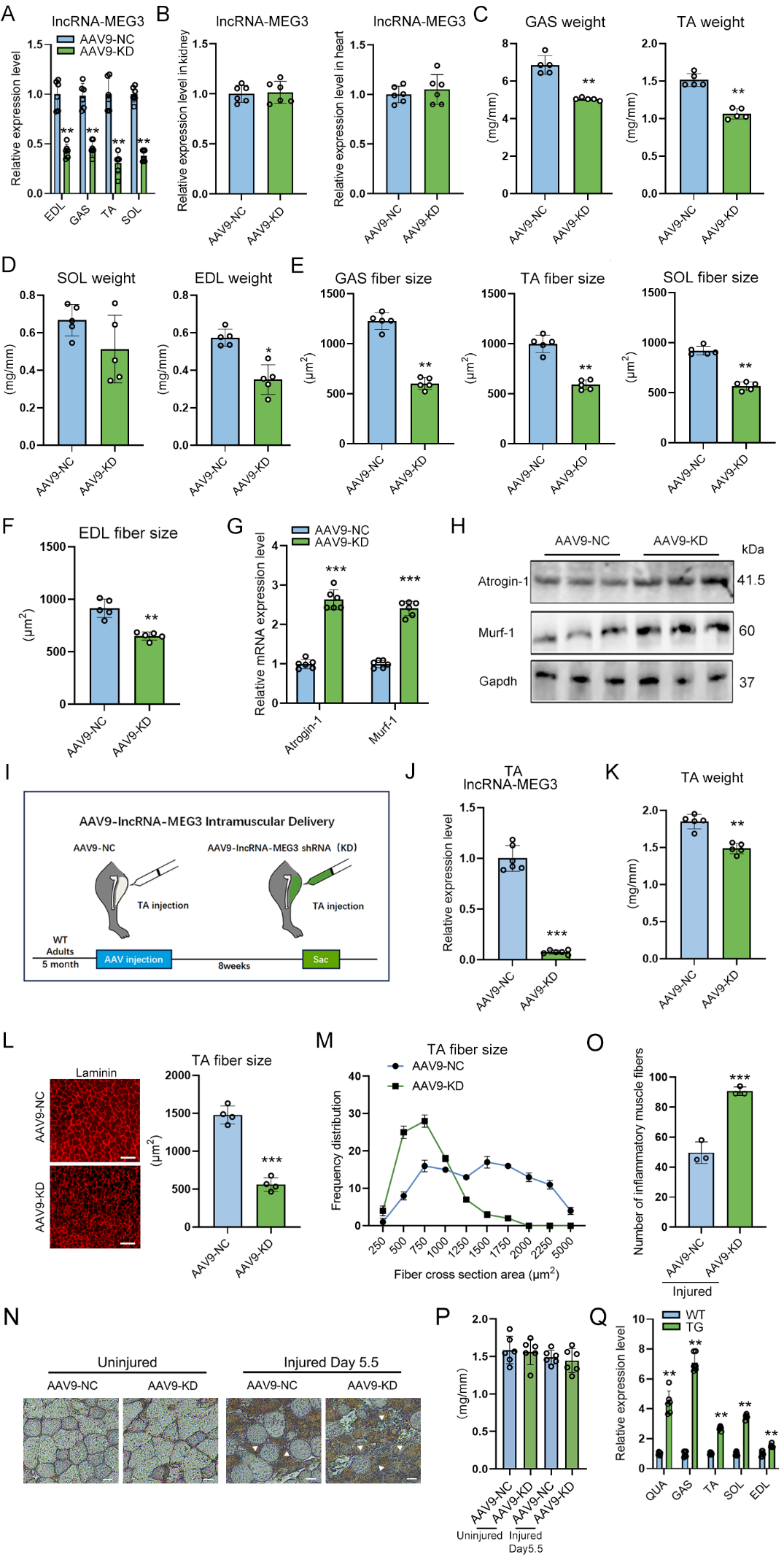
**

**Figure S3.** Impact of lncRNA-MEG3 on skeletal muscle morphology and function. (A-B) qRT-PCR analysis showing the expression of lncRNA-MEG3 in GAS, TA, EDL and SOL muscles, heart and kidney of mice treated with AAV9-NC or AAV9-KD (*n* = 6). (C, D) Muscle weights of GAS, TA, SOL, and EDL muscles normalized to tibia length in AAV9-NC and AAV9-KD mice (*n* = 5). (E, F) Cross-sectional fiber size of GAS, TA, SOL and EDL muscles in AAV9-NC and AAV9-KD mice (*n* = 5). (G, H) qRT-PCR (*n* = 6) and Western blot (*n* = 3) showing Atrogin-1 and Murf-1 expression in AAV9-NC and AAV9-KD mice. (I) Schematic of the knockdown strategy using AAV9-NC or AAV9-KD intramuscular injections into the tibialis anterior (TA) of 5-month-old WT mice. Experimental timeline created in Adobe Illustrator. (J) qRT-PCR analysis of lncRNA-MEG3 expression in TA muscles of AAV9-NC or AAV9-KD injected mice (*n* = 6). (K) TA muscle weight (*n* = 5), (L) average myofiber size, and (M) distribution of fiber sizes in AAV9-NC or AAV9-KD injected mice 8 weeks post-injection (*n* = 4). Scale bar = 100 μm. (N) Representative immunohistochemical staining showing IL-1β expression in cross-sections of injured and uninjured TA muscles from AAV9-NC and AAV9-lncRNA-MEG3 KD mice (*n* = 3). White arrows indicate inflammatory muscle fibers. Scale bar = 100 μm. (O) The number of inflammatory muscle fibers in injured TA muscles of AAV9-NC or AAV9-KD mice at 5.5 dpi (*n* = 3). (P) The weight of injured and uninjured TA muscles in AAV9-NC or AAV9-KD mice (*n* = 6). (Q) qRT-PCR showing the lncRNA-MEG3 expression in QUA, GAS, SOL, TA and EDL muscles of WT and lncRNA-MEG3 TG mice (*n* = 6). Data are mean ± SD; P-values were calculated using Student's *t*-test. **P* < 0.05, ***P* < 0.01 and ****P* < 0.001.


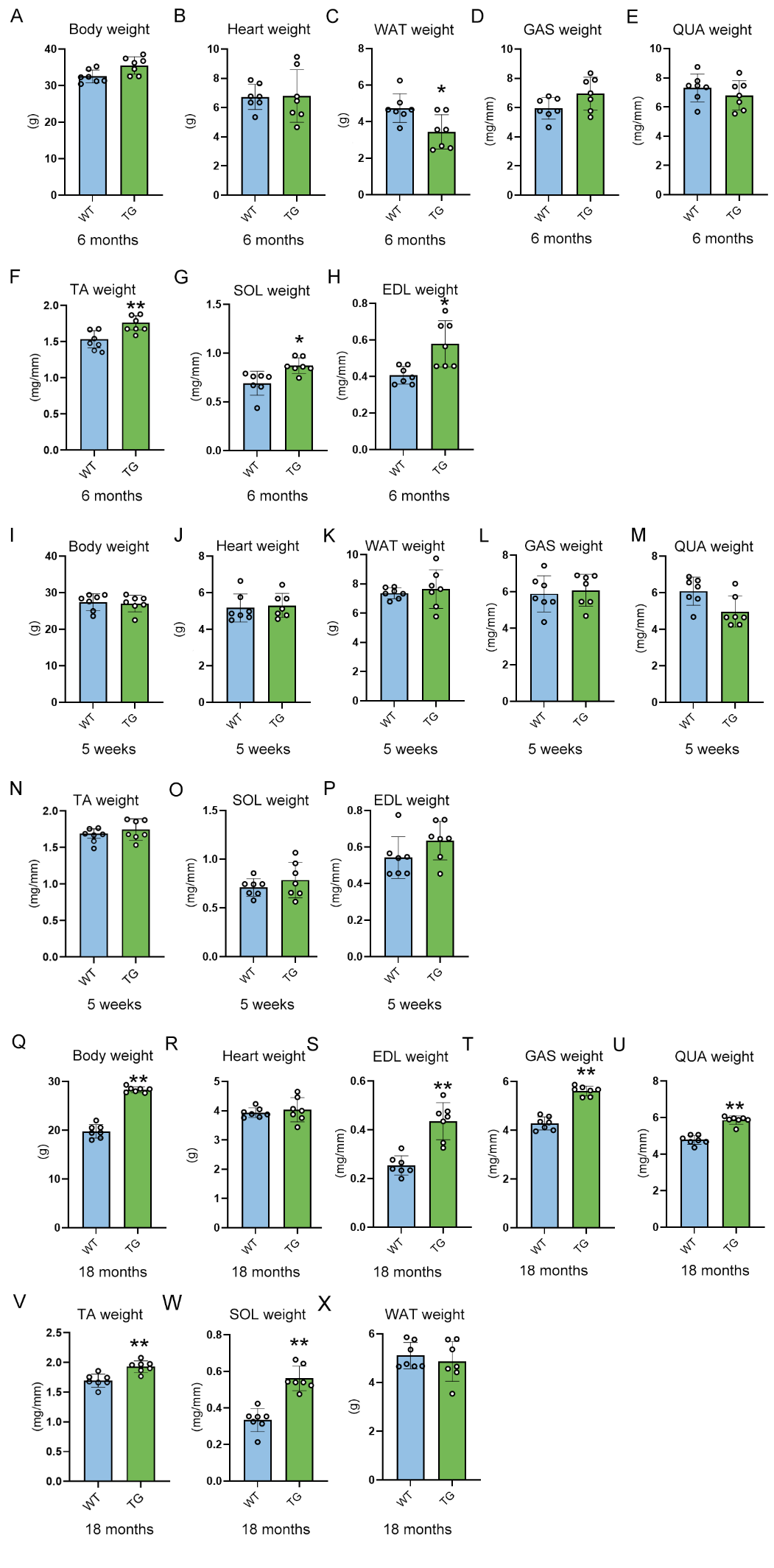


**Figure S4.** Overexpression of lncRNA-MEG3 in muscle for varying amounts of time. (A) Body, (B) heart, (C) WAT, (D) GAS, (E) QUA, (F) TA, (G) SOL and (H) ED weight relative to tibia length in lncRNA-MEG3 TG mice or wild-type controls 6 months. (I) Body, (J) heart, (K) WAT, (L) GAS, (M) QUA, (N) TA, (O) SOL and (P) EDL weight relative to tibia length in lncRNA-MEG3 TG mice or wild-type controls 5 weeks. (Q) Body, (R) heart, (S) EDL, (T) GAS, (U) QUA, (V) TA, (W) SOL and (X) WAT weight relative to tibia length in lncRNA-MEG3 TG mice or wild-type controls 18 months (*n* = 7). Data are mean ± SD; P-values were calculated using Student's *t*-test. **P* < 0.05, ***P* < 0.01.


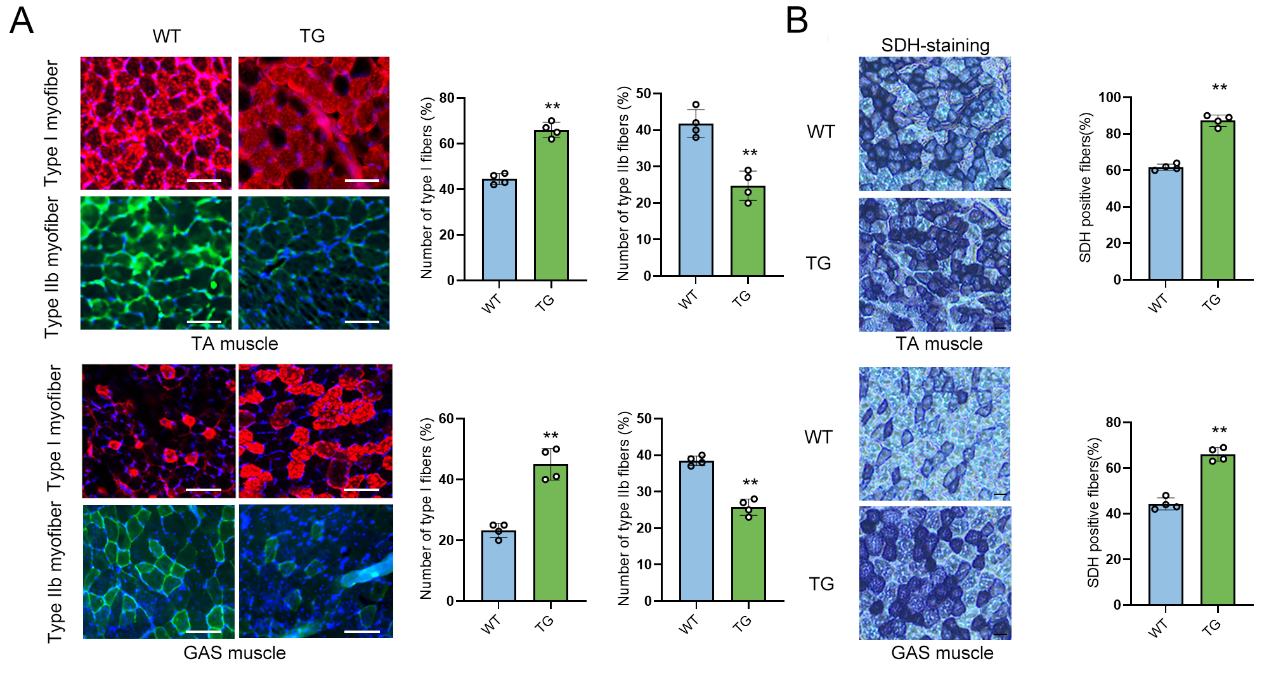


**Figure S5.** LncRNA-MEG3 TG mice regulate muscle fiber type conversion. (A) Representative immunofluorescence images of type I and type IIb myofibers in GAS and TA muscles from WT and lncRNA-MEG3 TG mice, with quantification of the percentage of each fiber type (*n* = 4). Scale bar = 50 μm. (B) Representative succinate dehydrogenase (SDH) staining in GAS and SOL muscles of WT and lncRNA-MEG3 TG mice, with quantification of SDH-positive fibers (*n* = 4). Scale bar = 50 μm. Data are mean ± SD; P-values were calculated using Student's *t*-test. **P* < 0.05, ***P* < 0.01.


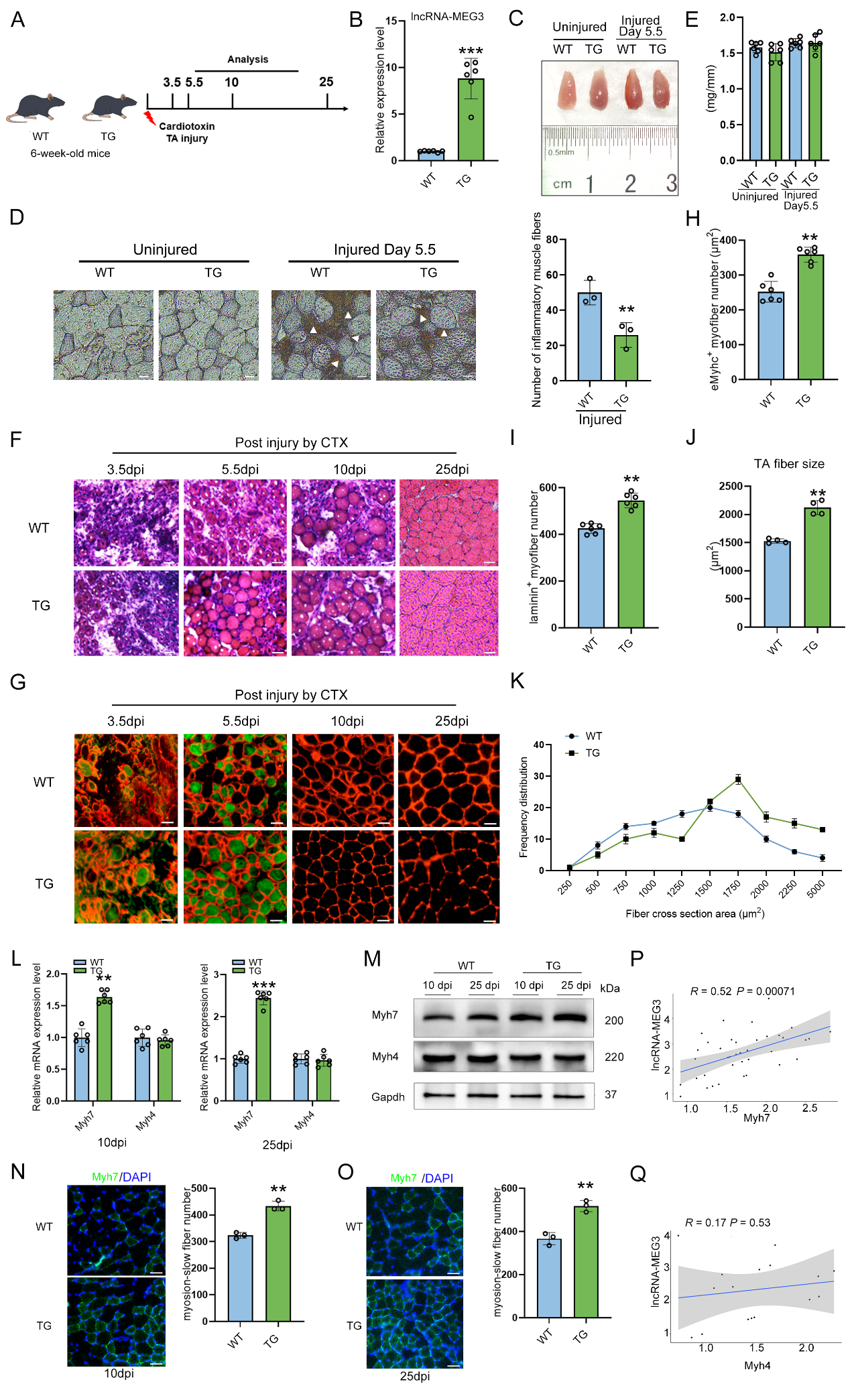


**Figure S6.** LncRNA-MEG3 overexpression promotes the slow fibers formation during skeletal muscle regeneration. (A) WT and lncRNA-MEG3 TG mice followed by cardiotoxin (CTX)-induced injury on mice TA muscle. Analysis was performed at various time points post-injury (3.5, 5.5, 10-, and 25-days post-injury (dpi)). (B) qRT-PCR showing lncRNA-MEG3 expression at 3.5 dpi (*n* = 6). (C) Representative images of injured and uninjured TA muscles from WT and TG mice (*n* = 6), Scale bar = 0.5 cm. (D) Representative immunohistochemical staining showing IL-1β expression in cross-sections of injured and uninjured TA muscles from WT and TG mice (*n* = 3). White arrows indicate inflammatory muscle fibers. Scale bar = 100 μm. The number of inflammatory muscle fibers in injured TA muscles of WT and TG mice at 5.5 dpi is shown on the right panel. (E) The weight of injured and uninjured TA muscles in WT and TG mice (*n* = 6). (F-G) Representative H&E staining (F) and immunostaining (G) of TA muscle cross-sections. Scale bars = 50 μm (*n* = 3). (H) Number of eMyHC^+^ myoﬁbers in TA muscle cross-sections at 5.5 dpi (*n* = 6). (I) Number of laminin ^+^ myoﬁbers in TA muscle cross-sections at 10 dpi (*n* = 6). (J) Average CSA of laminin ^+^ myoﬁbers in TA muscle cross-sections at 25 dpi (*n* = 4). (K) CSA distribution of laminin ^+^ myoﬁbers in TA muscle cross-sections at 25 dpi (*n* = 6). (L) qRT-PCR analysis Myh7 and Myh4 expression during skeletal muscle regeneration at 10 and 25 dpi (*n* = 6). (M) Western blot analyzing Myh7 and Myh4 expression during skeletal muscle regeneration (*n* = 3). (N, O) Representative Myh7 immunostaining on mice TA muscles at 10 and 25 dpi and quantification by ImageJ (*n* = 3). Scale bar = 50 μm. (P, Q) Pearson analysis of the correlation between lncRNA-MEG3, Myh7 and Myh4 during human skeletal muscle development. Data are mean ± SD; P-values were calculated using Student's *t*-test. **P* < 0.05, ***P* < 0.01 and ****P* < 0.001.


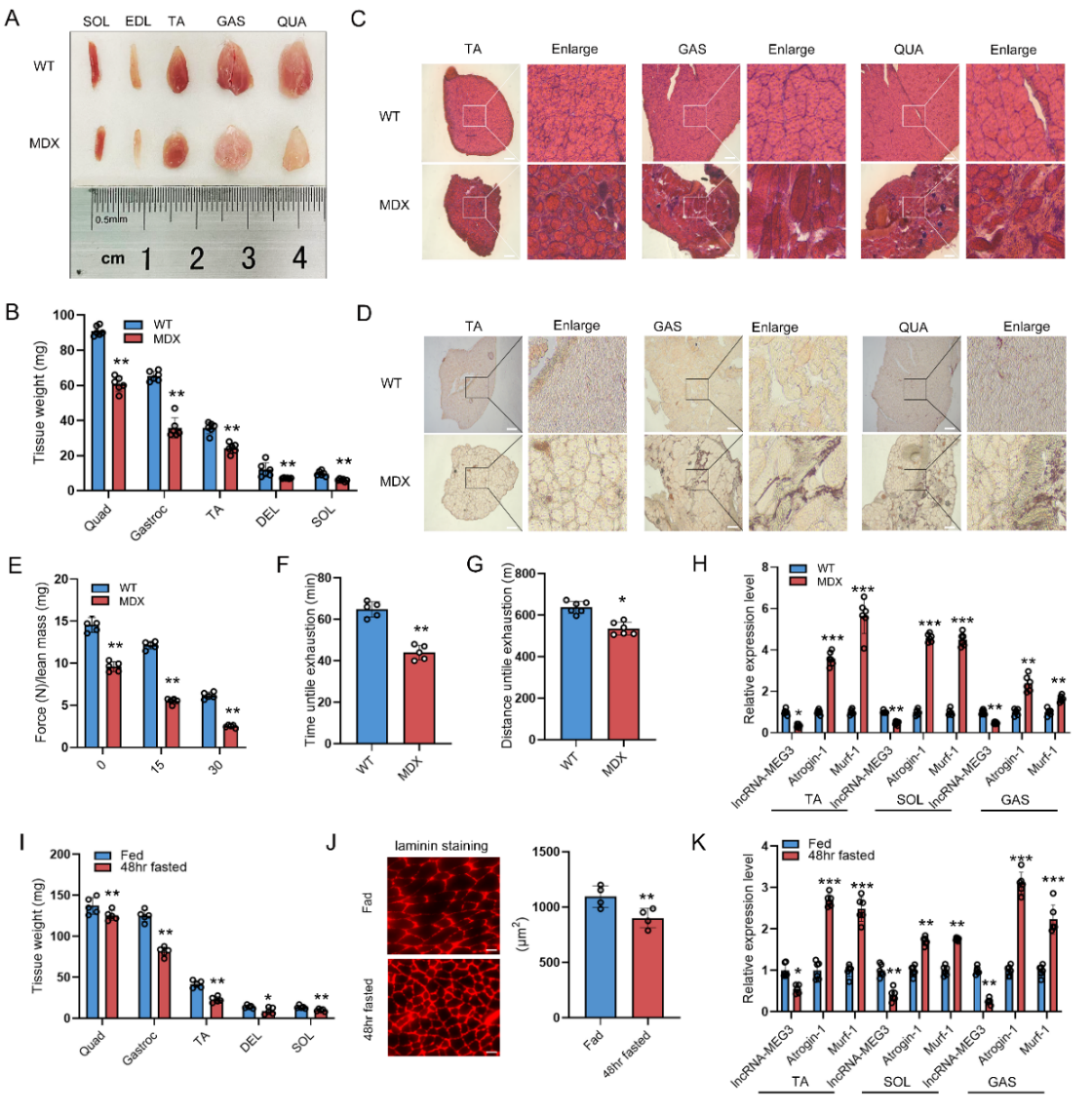


**Figure S7.** LncRNA-MEG3 responds to exercise by regulating SUZ12 LLPS to control the expression of FHL3. (A) Representative images of isolated skeletal muscles (SOL, EDL, TA, QUA, and GAS) from WT and MDX mice (*n* = 7). Scale bar = 0.5 cm. (B) Muscle weights normalized to tibia length in WT and MDX mice (*n* = 5). (C, D) H&E (C) and ORO (D) staining of skeletal muscles from WT and MDX mice, showing increased lipid accumulation in dystrophic muscles (*n* = 3). (E-G) Forelimb grip strength measurements (E) and treadmill endurance performance, including running time (F) and distance (G) in WT and MDX mice (*n* = 6). Forelimb grip strength was measured at 15 min intervals and standardized to lean body weight. (H) qRT-PCR analysis of lncRNA-MEG3 and muscular atrophy gene expression in WT and MDX muscle tissues (*n* = 6). (I) Skeletal muscle weights under fed and 48 h fasting conditions in mice (*n* = 5). (J) Representative immunofluorescence staining of muscle fibers showing changes in fiber size under fasting conditions (*n* = 4). Scale bar = 50 μm. (K) Expression of lncRNA-MEG3 and muscular atrophy genes under fasting conditions (*n* = 6). Data are mean ± SD; P-values were calculated using Student's *t*-test. **P* < 0.05, ***P* < 0.01 and ****P* < 0.001.


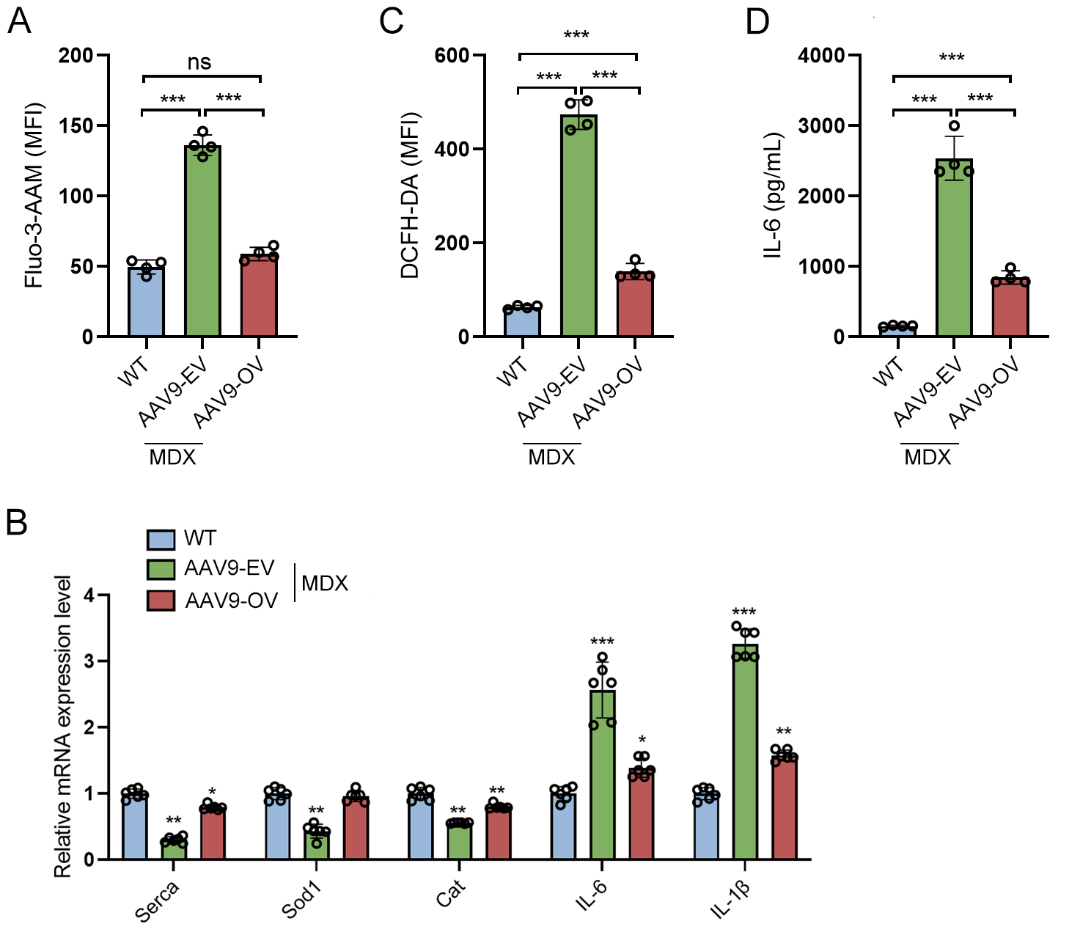


**Figure S8.** Effects of AAV9-mediated gene overexpression on oxidative stress and inflammatory responses in MDX mice. (A) Fluo-3-AM fluorescence intensity (MFI) indicating intracellular calcium levels in primary myoblasts of WT, AAV9-EV, and AAV9-OV MDX mice (*n* = 4). (B) qRT-PCR showing the expression level of Serca, oxidative stress-related genes (Sod1, Cat) and pro-inflammatory cytokines (IL-6, IL-1β) in primary myoblasts of WT, AAV9-EV, and AAV9-OV MDX mice (*n* = 6). (C) DCFH-DA fluorescence intensity (MFI) representing reactive oxygen species (ROS) levels in primary myoblasts of WT, AAV9-EV, and AAV9-OV MDX mice (*n* = 4). (D) Serum IL-6 levels measured by ELISA (*n* = 4). Data are mean ± SD; P-values were calculated using Student's *t*-test. **P* < 0.05, ***P* < 0.01 and ****P* < 0.001.


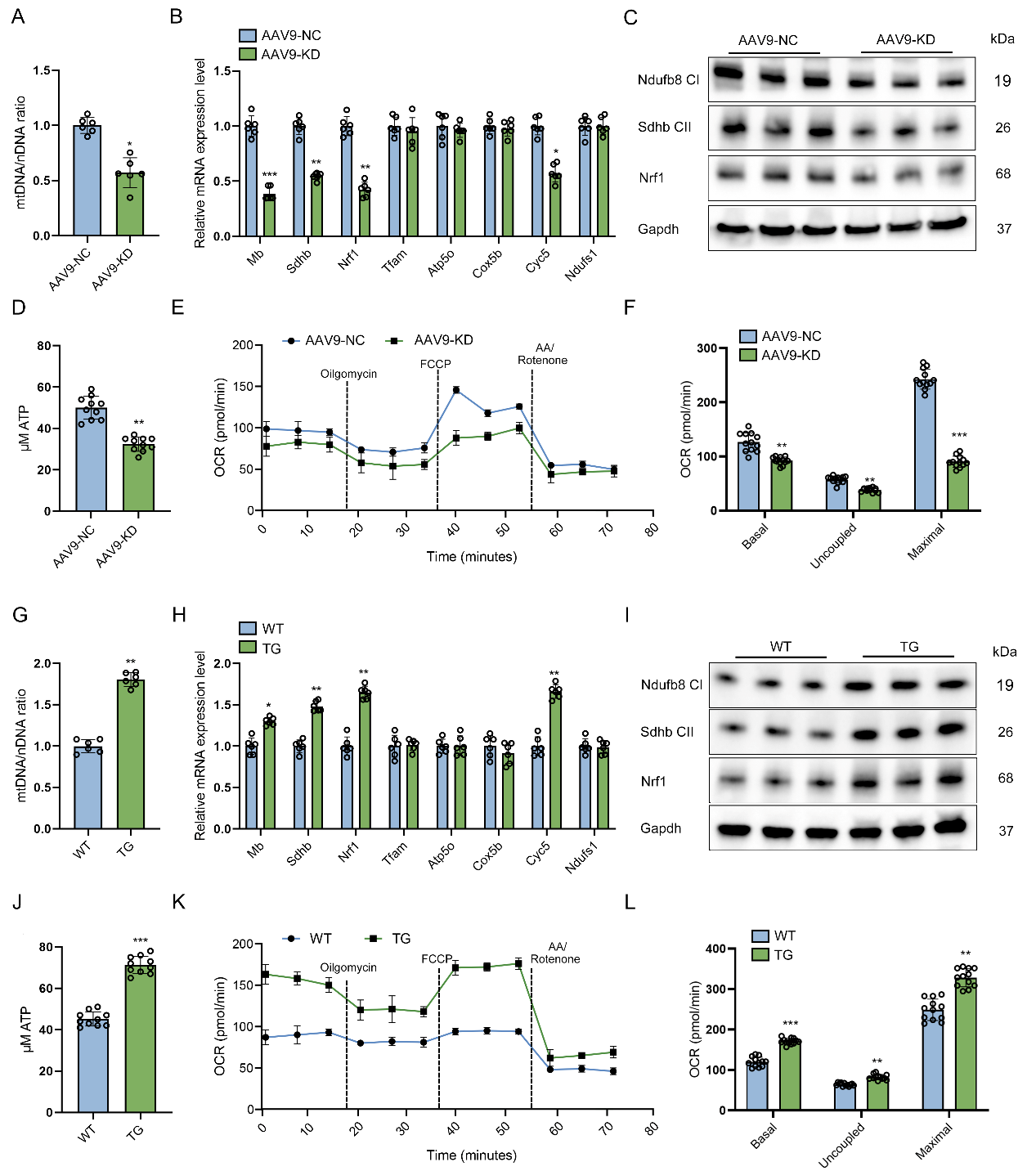


**Figure S9.** Effects of lncRNA-MEG3 knockdown and overexpression on mitochondrial function in skeletal muscle. (A) Mitochondrial DNA (mtDNA) levels quantified by qRT-PCR using nuclear DNA (nDNA) as a reference after lncRNA-MEG3 knockdown (*n* = 6). (B) qRT-PCR analysis of mitochondrial biogenesis- and oxidative phosphorylation (OxPhos)-related genes in the GAS muscle following lncRNA-MEG3 knockdown (*n* = 6). (C) Representative Western blot of OxPhos complex proteins after lncRNA-MEG3 knockdown (*n* = 3). (D) ATP levels measured by the O2K system (*n* = 8). (E-F) Oxygen consumption rate (OCR) in GAS myofibers measured using a O2K system, showing basal respiration, ATP-linked respiration (uncoupled, calculated as the difference before and after oligomycin), and maximal respiration (difference after FCCP and antimycin A (AA)/rotenone) after lncRNA-MEG3 knockdown (*n* = 12). (G) mtDNA levels quantified as in (A) after lncRNA-MEG3 overexpression (*n* = 6). (H) qRT-PCR analysis of mitochondrial biogenesis- and OxPhos-related genes following lncRNA-MEG3 overexpression (*n* = 6). (I) Representative Western blot of OxPhos complex proteins after lncRNA-MEG3 overexpression (*n* = 3). (J) ATP levels measured as in (D) (*n* = 8). (K-L) OCR analysis as in (E-F) after lncRNA-MEG3 overexpression (*n* = 12). Data are mean ± SD; P-values were calculated using Student's *t*-test. **P* < 0.05, ***P* < 0.01 and ****P* < 0.001.


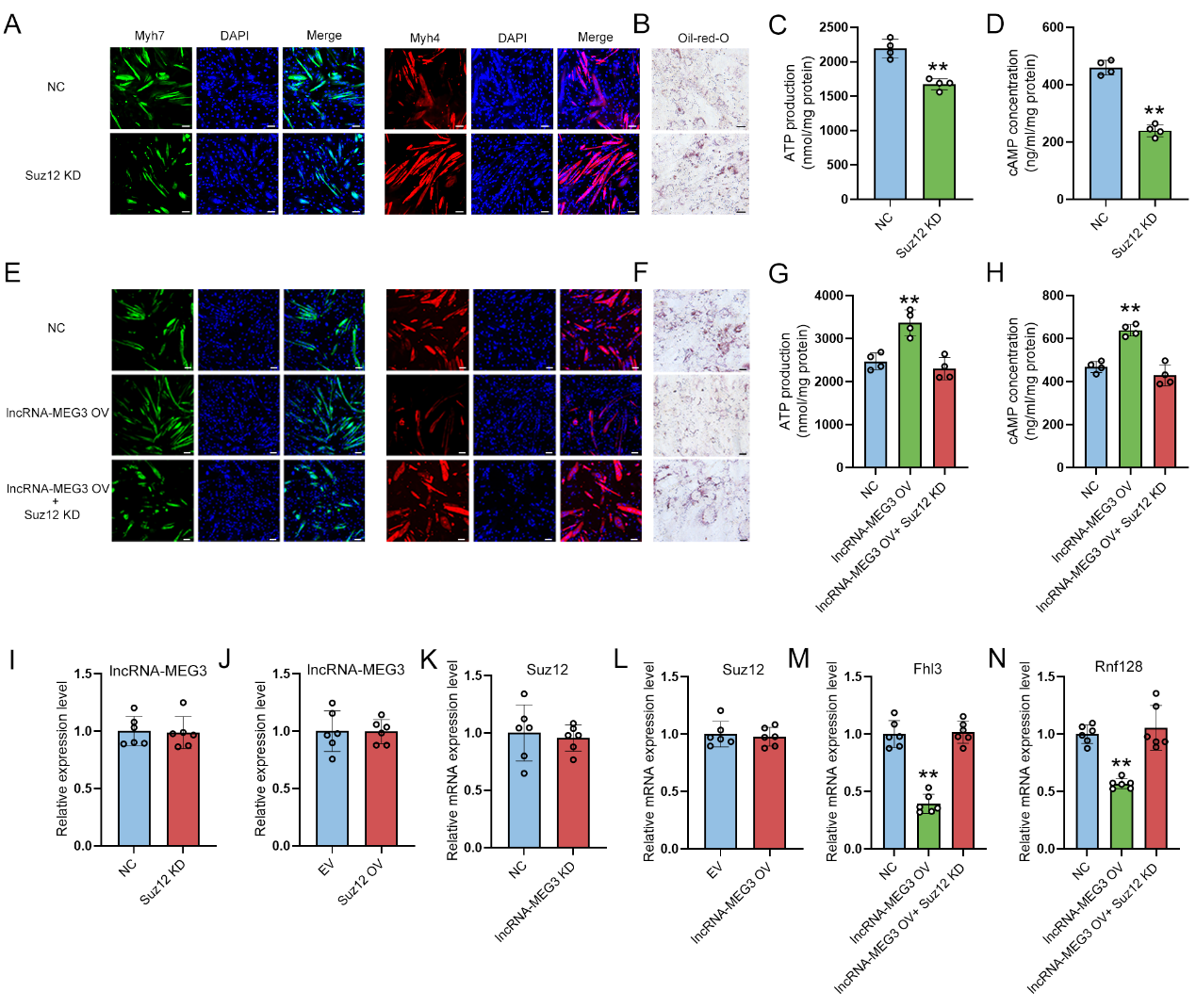


**Figure S10.** LncRNA-MEG3 regulates fiber transformation and lipid droplet formation through binding with SUZ12. (A) Representative Myh7 and Myh4 immunostaining on C2C12 myotube and quantified by ImageJ after Suz12 knockdown (*n* = 3). Scale bar = 100 μm. (B) ORO staining and quantification of lipid droplets in muscle sections after Suz12 knockdown (*n* = 3). Scale bar = 100 μm. (C, D) ATP and cAMP production after Suz12 knockdown (*n* = 4). (E) Representative Myh7 and Myh4 immunostaining after C2C12 myoblasts were transfected with NC, lncRNA-MEG3 overexpression vector (lncRNA-MEG3 OV) and lncRNA-MEG3 OV + Suz12 siRNA (Suz12 KD) (*n* = 3). Scale bar = 100 μm. (F) Representative ORO staining after C2C12 myoblasts were transfected with NC, lncRNA-MEG3 OV and lncRNA-MEG3 OV + Suz12 KD (*n* = 3). Scale bar = 100 μm. (G, H) ATP and cAMP production after C2C12 myoblasts were transfected with NC, lncRNA-MEG3 OV and lncRNA-MEG3 OV + Suz12 KD (*n* = 4). (I, J) qRT-PCR showing the expression of lncRNA-MEG3 after Suz12 knockdown and overexpression (*n* = 6). (K, L) qRT-PCR showing the expression of Suz12 after lncRNA-MEG3 knockdown and overexpression (*n* = 6). (M, N) qRT-PCR showing the expression of Fhl3 and Rnf128 after transfected with NC, lncRNA-MEG3 OV and lncRNA-MEG3 OV + Suz12 KD in C2C12 myoblasts (*n* = 6). Data are mean ± SD; P-values were calculated using Student's *t*-test. **P* < 0.05, ***P* < 0.01.


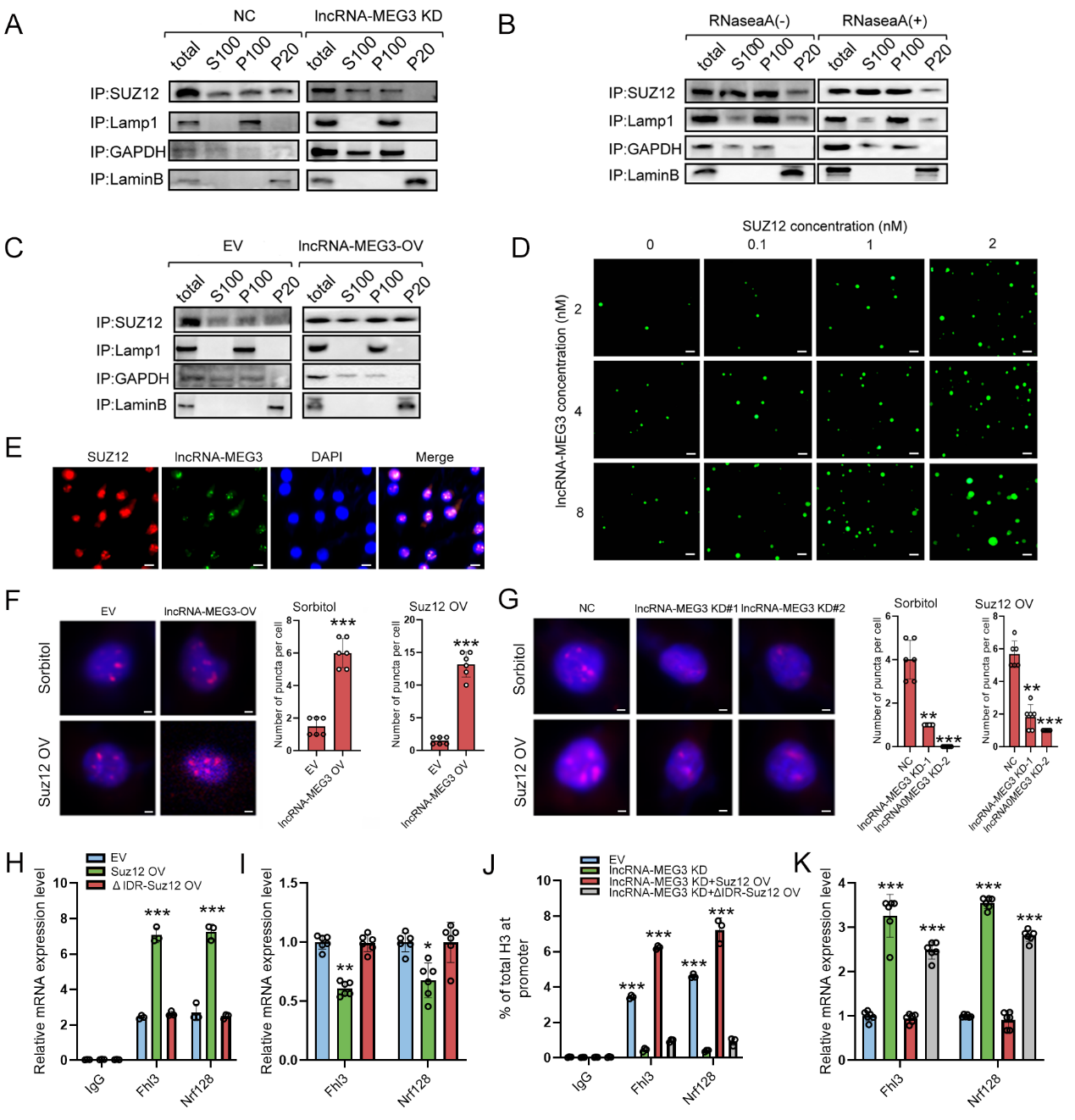


**Figure S11.** Subcellular distribution of SUZ12 is regulated by RNA integrity and lncRNA-MEG3 overexpression. (A-C) Co-immunoprecipitation showing interaction of SUZ12 with nuclear (Lamin B1) and cytoplasmic (GAPDH) markers upon lncRNA-MEG3 shRNA (A), RNAaseaA (B) and lncRNA-MEG3 overexpression vector (C) treated in C2C12 myoblasts (*n* = 3). (D) *In vitro* phase separation of SUZ12 protein in the presence of increasing concentrations of lncRNA-MEG3 RNA. Droplet size and number increase in a concentration-dependent manner (*n* = 3). Scale bar = 10 μm. (E) Immunofluorescence of SUZ12 (red) and lncRNA-MEG3 (green) in C2C12 myoblasts showing co-localization in nuclear puncta (*n* = 3). Nuclei are stained with DAPI (blue). Scale bar = 100 μm. (F-G) The impact of knockdown (F) and overexpression (G) of lncRNA-MEG3 on SUZ12 condensate formation in C2C12 myoblasts, under conditions of Suz12 overexpression vector and sorbitol treatment (*n* = 6). Quantification of SUZ12-positive puncta per cell is shown (right panel). Scale bar = 10 μm. (H) ChIP-qPCR analysis showing the enrichment of H3K27me3 at the promoter regions of Fhl3 and Rnf128 in C2C12 myoblasts transfected with an empty vector (EV), Suz12 overexpression vector (Suz12 OV), or a deletion mutant of Suz12 lacking the IDR domain (ΔIDR-Suz12 OV) (*n* = 3). (I) qRT-PCR analysis showing the expression levels of Fhl3 and Rnf128 in C2C12 myoblasts transfected with EV, Suz12 OV, or ΔIDR-Suz12 OV (*n* = 6). (J) ChIP-qPCR analysis showing the enrichment of H3K27me3 at the promoter regions of Fhl3 and Rnf128 in C2C12 myoblasts transfected with EV, lncRNA-MEG3 shRNA (lncRNA-MEG3 KD), lncRNA-MEG3 KD + Suz12 OV and ΔIDR-Suz12 OV + lncRNA-MEG3 KD (*n* = 3). (K) qRT-PCR analysis showing the expression levels of Fhl3 and Rnf128 in C2C12 myoblasts transfected with EV, lncRNA-MEG3 KD, lncRNA-MEG3 KD + Suz12 OV and ΔIDR-Suz12 OV + lncRNA-MEG3 KD (*n* = 6). Data are mean ± SD; P-values were calculated using Student's *t*-test. **P* < 0.05, ***P* < 0.01 and ****P* < 0.001.


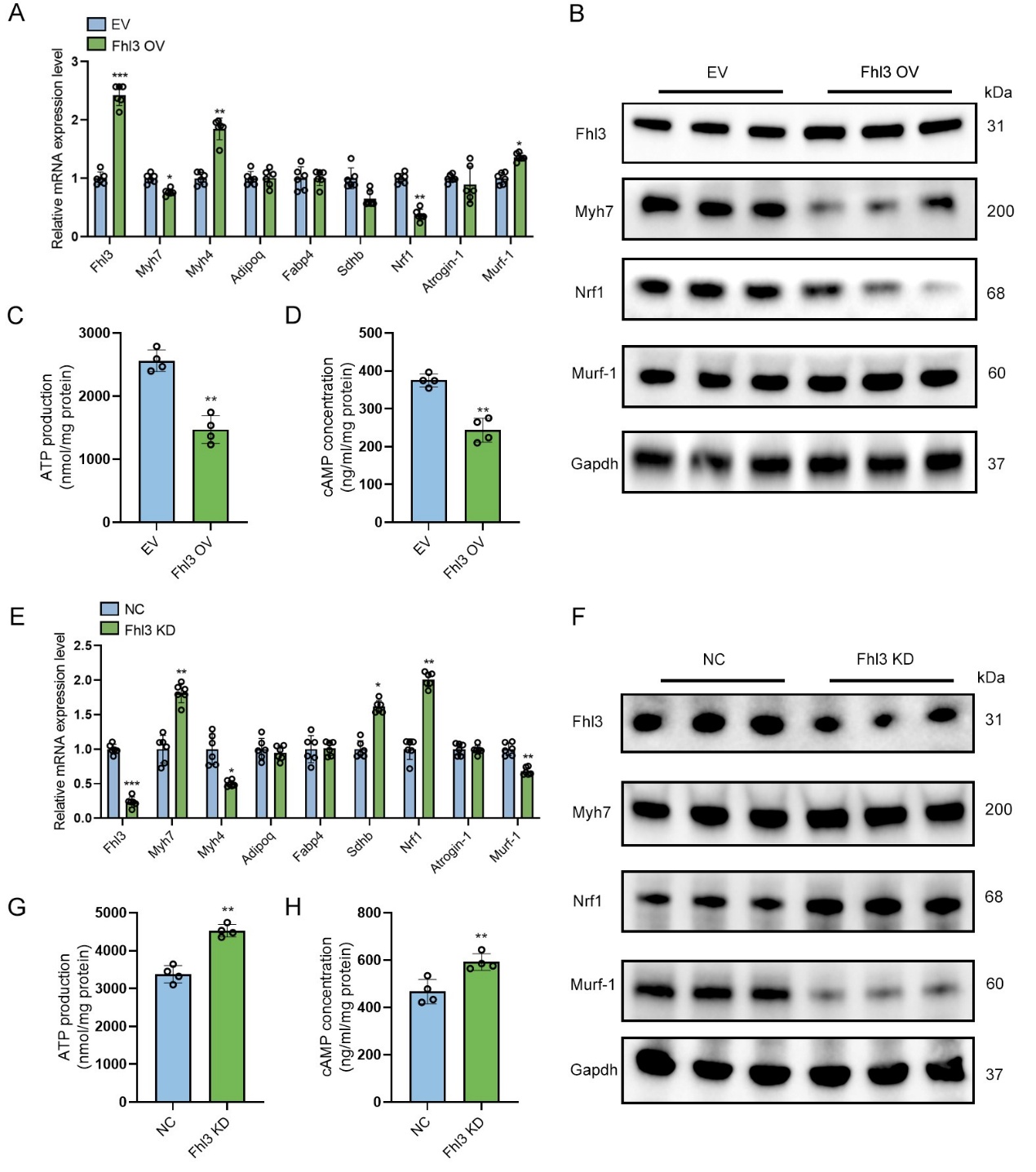


**Figure S12.** Fhl3 regulates muscle fiber type conversion and mitochondrial function. (A, B) qRT-PCR (*n* = 6) and Western blot (*n* = 3) showing the effects of Fhl3 overexpression on the expression of skeletal muscle-specific, muscle atrophy, adipogenesis, and mitochondrial biogenesis markers in C2C12 myoblasts. (C, D) The impact of Fhl3 overexpression on ATP synthesis and cAMP production in C2C12 myoblasts (*n* = 4). (E, F) qRT-PCR (*n* = 6) and Western blot (*n* = 3) showing the effects of Fhl3 knockdown on the expression of skeletal muscle-specific, muscle atrophy, adipogenesis, and mitochondrial biogenesis markers in C2C12 myoblasts. (G, H) The impact of Fhl3 knockdown on ATP synthesis and cAMP production in C2C12 myoblasts (*n* = 4). Data are mean ± SD; P-values were calculated using Student's *t*-test. **P* < 0.05, ***P* < 0.01 and ****P* < 0.001.


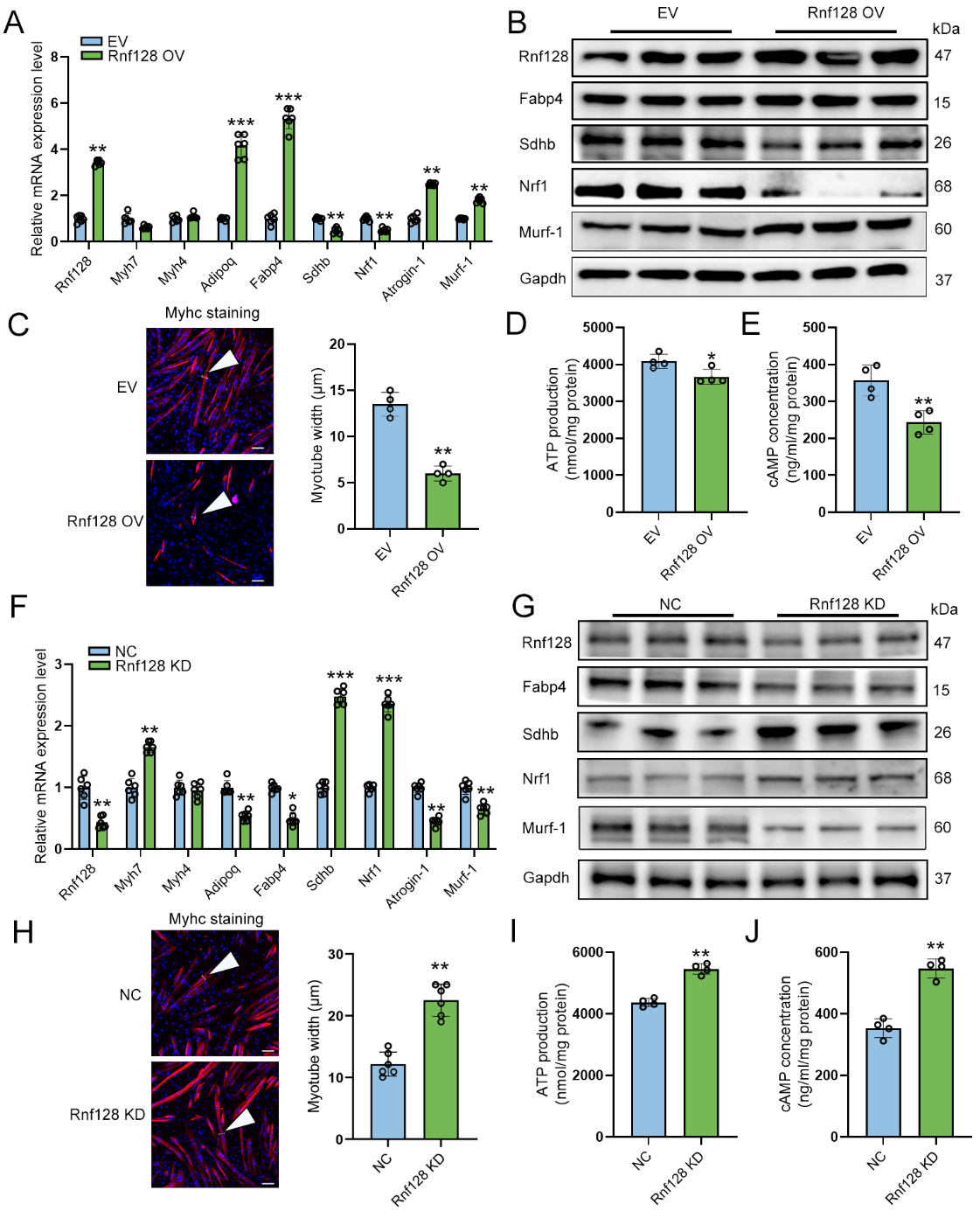


**Figure S13.** Rnf128 regulates muscle atrophy and mitochondrial function. (A, B) qRT-PCR (*n* = 6) and Western blot (*n* = 3) showing the expression of skeletal muscle-specific, muscle atrophy, adipogenesis, and mitochondrial markers in C2C12 myoblasts after Rnf128 overexpression. (C) Representative Myhc staining and quantification of myotube width in C2C12 myoblasts after Rnf128 overexpression (*n* = 4). Scale bar = 100 μm. (D, E) Analysis of the effects of Rnf128 overexpression on ATP synthesis and cAMP production in C2C12 myoblasts (*n* = 4). (F, G) qRT-PCR (*n* = 6) and Western blot (*n* = 3) showing the expression of skeletal muscle-specific, muscle atrophy, adipogenesis, and mitochondrial biogenesis markers in C2C12 myoblasts after Rnf128 knockdown. (H) Representative Myhc staining and quantification of myotube width in C2C12 myoblasts after Rnf128 knockdown (*n* = 6). Scale bar = 100 μm. (I, J) Analysis of the effects of Rnf128 knockdown on ATP synthesis and cAMP production in C2C12 myoblasts (*n* = 4). Data are mean ± SD; P-values were calculated using Student's *t*-test. **P* < 0.05, ***P* < 0.01 and ****P* < 0.001.
